# A comprehensive item bank of internal validity issues of relevance to in vitro toxicology studies

**DOI:** 10.1080/2833373X.2024.2418045

**Published:** 2024-10-31

**Authors:** Gunn E. Vist, Heather M. R. Ames, Gro H. Mathisen, Trine Husøy, Camilla Svendsen, Anna Beronius, Emma Di Consiglio, Ingrid L. Druwe, Thomas Hartung, Sebastian Hoffmann, Carlijn R. Hooijmans, Kyriaki Machera, Pilar Prieto, Joshua F. Robinson, Erwin Roggen, Andrew A. Rooney, Nicolas Roth, Eliana Spilioti, Anastasia Spyropoulou, Olga Tcheremenskaia, Emanuela Testai, Mathieu Vinken, Paul Whaley

**Affiliations:** a Norwegian Scientific Committee for Food and Environment, Norwegian Institute of Public Health, Oslo, Norway; b Division for Health Services, Norwegian Institute of Public Health, Oslo, Norway; c Department of Food Safety, Norwegian Institute of Public Health, Oslo, Norway; d Department of Chemical Toxicology, Norwegian Institute of Public Health, Oslo, Norway; e Institute of Environmental Medicine, Karolinska Institutet, Stockholm, Sweden; f Environment & Health Department, Italian National Institute of Health (ISS), Rome, Italy; g United States Environmental Protection Agency, Office of Research and Development, Center for Public Health and Environmental Assessments, Research Triangle Park, NC, USA; h Center for Alternatives to Animal Testing, Johns Hopkins University, Bloomberg School of Public Health, Baltimore, MD, USA; i CAAT Europe, University of Konstanz, Konstanz, Germany; j Evidence-Based Toxicology Collaboration, Johns Hopkins University Bloomberg School of Public Health, Baltimore, MD, USA; k Seh Consulting + Services, Paderborn, Germany; l Department of Anesthesiology, Pain and Palliative Care, Radboud University Medical Centre, Nijmegen, Netherlands; m Laboratory of Toxicological Control of Pesticides, Scientific Directorate of Pesticides’ Control and Phytopharmacy, Benaki Phytopathological Institute, Kifissia, Greece; n European Commission, Joint Research Centre (JRC), Ispra, Italy; o Department of Obstetrics, Gynecology & Reproductive Sciences, University of California, San Francisco (UCSF), San Francisco, CA, USA; p 3Rs Management and Consulting ApS, Lyngby, Denmark; q Division of Translational Toxicology, National Institute of Environmental Health Sciences, Research Triangle Park, NC, USA; r Department of Pharmaceutical Sciences, University of Basel, Basel, Switzerland; s Swiss Centre for Applied Human Toxicology, Basel, Switzerland; t Department of Pharmaceutical and Pharmacological Sciences, Vrije Universiteit Brussel, Brussels, Belgium; u Lancaster Environment Centre, Lancaster University, Lancaster, UK

**Keywords:** In vitro methods, toxicology, NAMs, risk of bias, internal validity

## Abstract

**Context:**

*In vitro* toxicology studies are increasingly being included as evidence in systematic reviews and chemical risk assessments. INVITES-IN, a tool for assessing the internal validity of *in vitro* studies, is currently under development. The first step in developing INVITES-IN involves the creation of an “item bank,” an overview of study assessment concepts that may be relevant to evaluating the internal validity of *in vitro* toxicology studies. The item bank and methodology for its creation presented in this manuscript are intended to be a general resource for supporting the development of appraisal tools for *in vitro* toxicology studies and potentially other study designs.

**Methods:**

We derived the item bank from seven literature sources (one existing item bank created from a systematic review of assessment criteria for *in vitro* studies, and six purposively sampled study appraisal tools) and the transcripts of three focus groups. Assessment criteria plausibly relating to internal validity were abstracted from the literature sources and focus group transcripts, disaggregated into individual criteria, then normalised to express in the simplest achievable language the core issue in each criterion – an “item bank” of assessment concepts. The items were then mapped onto a set of bias domains. We conducted simple descriptive statistical analyses and visualisations to describe patterns in the dataset and developed recommendations for the use and development of the item bank.

**Results:**

The item bank contains 405 items of potential relevance to evaluating the internal validity of *in vitro* toxicology studies.

**Discussion:**

To our knowledge, this is the second item bank of any kind to have been created for toxicology studies, and the first to use focus groups as a data source alongside literature analysis. The large number of items contributed by focus group discussions suggests this is an efficient method for capturing internal validity issues that are not easily identifiable in the literature. We believe our item bank and methodology for its creation will be a useful resource for supporting the development of appraisal tools. Due to the broad applicability of many items in the item bank, it may be informative for study designs beyond the *in vitro* domain.

## 1. Introduction

Systematic reviews are increasingly being conducted in toxicology, environmental health, and chemical risk assessment (Menon, Struijs, and Whaley 2022). An important part of the review methodology is the evaluation of internal validity, i.e. the extent to which the design, conduct, and reporting of a study are likely to have prevented bias. While many tools have been created for assessing validity of *in vitro* studies, to date there is no consensus as to which, if any, of these tools ought to be used for assessment of the internal validity of *in vitro* studies (Tran et al. 2021). To address this issue, we are conducting the INVITES-IN (IN VITro Experimental Studies INternal validity) project. The objective of INVITES-IN is to select, modify, or develop an appraisal tool for assessing the internal validity of *in vitro* studies, with an initial focus on eukaryotic cell culture studies.

The creation of INVITES-IN consists of four methodological stages, that we refer to as “studies”:

- Study 1. Creating a comprehensive database of issues that may affect the internal validity of *in vitro* studies of any design.
- Study 2. Prioritising issues from the database according to their relevance for the assessment of the internal validity of eukaryotic cell culture systems.
- Study 3. Creating a beta version of an appraisal tool based on analysis of the prioritised issues identified in Study 2.
- Study 4. Creating a final release version of the tool, based on user-testing of the beta version.

Due to the complexity of the INVITES-IN project, we are publishing our methods and results in three manuscripts. The present manuscript describes the methods and results for Study 1. A second manuscript will describe Study 2. The third manuscript will describe Studies 3 and 4. The planned methods for all four INVITES-IN studies are described in two protocols (Mathisen et al. 2023; Svendsen et al. 2023).

## 2. Objectives

The objective of this study is to create a comprehensive database of issues relating to the design, conduct, and reporting of *in vitro* studies that may introduce bias into their results or findings. Following Page et al. (2020) and Whiting et al. (2017), we call such a database an “item bank.”

It should be noted that while INVITES-IN is ultimately aimed at creating a tool for assessing the internal validity of studies of eukaryotic cell culture systems, the item bank includes issues for *in vitro* studies of any design.

## 3. Methods

In their proposed framework for developing quality assessment tools, Whiting and colleagues recommend an “item generation” step be part of tool development methodology (Whiting et al. 2017). However, what constitutes best practice in item generation is not, as far as we are aware, a settled matter. Our item bank development methodology follows Whaley et al. (2024) and consists of two main stages, summarised below then described more fully in the subsequent sections of this manuscript. The full details of the methodology are in our preregistered study protocol (Svendsen et al. 2023).

### 3.1. Stage 1. Issue discovery

The objective of this stage is to identify a comprehensive set of issues relating to the design, conduct, and reporting of *in vitro* studies that may introduce bias into their results or findings.

Our method for this stage is to abstract from (a) a purposive sample of tools designed for assessing the internal validity of scientific studies and (b) focus group transcripts a comprehensive list of issues that researchers believe may impact the internal validity of *in vitro* studies.

We chose this approach because three separate reviews have indicated that none of the existing quality assessment tools for *in vitro* studies individually cover all critical aspects for assessing the internal validity of *in vitro* studies (Paiva Barbosa et al. 2023; Tran et al. 2021; Whaley et al. 2024), and we believed that focus groups with *in vitro* experts would capture important bias issues that have not yet been incorporated into validity assessment tools.

### 3.2. Stage 2. Issue analysis

The objective of this stage is to derive linguistically minimalist expressions of the study assessment concepts identified in stage 1 (i.e. create the database of “items”) and organise these items according to bias themes.

We chose this approach because the way that bias issues are presented in appraisal tools and focus group discussions is heterogeneous and complex. This can make it challenging to discern precisely what issue is being addressed by a criterion in a tool or a point of expert discussion, and to compare criteria or discussion points to determine if they are in fact about the same issue. We therefore disaggregated complex appraisal criteria into their component issues and sought to eliminate irrelevant linguistic differences in how appraisal criteria are expressed in different tools and contexts (a process we refer to as “normalisation”). We also organised items into bias domains to help structure the database and make it easier to use in later stages of the INVITES-IN project.

The process for issue analysis is shown in Supplemental Material 5 (sheet 4) and illustrated in Figure 2.

#### 3.2.1. Issues discovery

We used two sources for creation of the item bank: literature (six appraisal tools and one systematic review, see section 2.1.1 for more details); and experts with extensive experience with *in vitro* studies (called “domain experts”) participating in focus group discussions (see section 2.1.2). We conducted the literature analysis first, then supplemented the literature with the additional items discovered through the focus group discussions.

##### 3.2.1.1. Literature sources.

Items were abstracted from the literature sources as listed in the protocol (Svendsen et al. 2023) with one additional tool (ROBINS-E). The literature sources included a selection of published and widely used validity assessment tools, and an existing item bank covering study appraisal concepts included in 67 *in vitro* appraisal tools (Whaley, Hooijmans, and Wattam 2023).

The literature sources included tools for assessment of *in vitro* studies, human studies, and animal experimental studies. We included assessment tools for non *in vitro* studies based on our experience that assessment concepts are quite generalizable for different types of study, and therefore that newer risk of bias tools even for epidemiological research may include concepts that could be applicable to *in vitro* studies. This way we aimed to capture emerging developments in risk of bias assessment methods and criteria that may not yet have been incorporated into tools within the *in vitro* toxicology literature. The literature sources are listed in Table 1.

##### 3.2.1.2. Focus groups.

###### 3.2.1.2.1. Recruitment of focus group participants.

Eligible focus group discussion participants were scientists with or without systematic review experience that are active in the field of *in vitro* studies in academia, governmental institutions (including risk assessment institutions and research institutes) or private research institutes, at postdoctoral level or higher, and level B1 English speakers. By “active in the field of *in vitro* studies” we mean having practical experience in conducting *in vitro* studies, and/or experience in analysing or evaluating data from such studies.

The focus group discussion participants were nominated by members in the project group and the scientific advisory group (see “project governance”).

Potential participants were invited to participate with the aim of achieving (1) an equal gender distribution, (2) a reasonable demographic and regional distribution, and (3) enough participants to have three groups of six to eight people. Potential participants were contacted via email and sent information about the project, the purpose of the focus group discussions and the method, that the results would be anonymised, the withdrawal procedure, the financial source, and the approximate time for the focus group discussions (Supplemental Material 1). Thirty potentially eligible participants were invited in the period of 16 May to 12 June 2023. All participants who accepted the invitation actively confirmed their consent to participate in the research.

Twenty participants were recruited and participated in the focus group discussions: six in group 1, eight in group 2, and six in group 3. An overview of the characteristics of the participants is given in Supplemental Material 2. The participants were mostly from Europe and North America, and worked in various sectors (e.g. government agencies, academia). Most had extensive expertise with *in vitro* studies and chemical risk assessment. While we sought to have an equal distribution of gender, the individuals who had the expertise along with availability to participate were overwhelmingly female (15 female to 5 males).

###### 3.2.1.2.2. Focus group discussion sessions.

Focus group participants were engaged in open-ended conversation about how, in their opinion, bias can be introduced into *in vitro* studies. Each focus group met twice virtually, for 90 minutes in each meeting, using Microsoft Teams. The focus group discussions were audio-recorded and machine-transcribed using the transcribe function in Microsoft Teams classic (June 2023), with transcription errors corrected only when it was judged to have changed the meaning of the concepts of interest to the item identification process. Transcripts were anonymized and original recordings were deleted after 30 days. The focus group discussions were moderated by one investigator (PW) and assisted by another investigator (GEV). The discussion was structured according to the bias domains used to organise the items extracted from the literature. For each domain, the moderator presented the name of the bias domain, the number of items that had been associated with the bias from the literature-based component of the item bank, and the definition of the bias. Discussion was paced by the moderator to attempt to work through all domains across the two sessions. The order in which domains were presented was different for each group, with priority given to domains that had overall received less discussion in previous focus groups (with the exception that when starting a discussion with a new group, a more intuitive domain would be chosen to get conversation going). Participants were led in discussion of how the domain might be relevant to the *in vitro* research context, and asked for examples from their practical research experience of how systematic error can be introduced into an *in vitro* study. A third investigator (HMA) participated in all sessions, taking back-up notes in case of technical failure and to support any necessary correction of the machine-generated transcripts. The information shown to the participants of each focus group is in Supplemental Material 3.

Participants were given the option to send by email their thoughts and considerations on the relevance of the discussed bias domains and items for *in vitro* studies to the project group within a week after each discussion. One comment was received and added to the analysis.

### 3.3. Issue analysis

The analytical process by which the bias issues abstracted from the literature sources and focus groups are interpreted into items is shown in Figure 2. In the first stage of issue analysis, bias-related issues were abstracted from the included literature sources and the focus group transcripts. For the literature sources, two investigators (PW and GEV) independently abstracted bias-related criteria from each source, focusing on any section of a source that seemed to be presenting a list or table of study quality criteria. They discussed and reconciled inconsistencies in the results of their abstraction process.

The focus group transcripts were independently annotated in Microsoft Word by two investigators (HMA and GEV) for instances of participants indicating specific ways in which bias can be introduced into an *in vitro* study (see Supplemental Material 4). After each transcript was independently annotated, the two copies were combined into a single document. Two investigators (HMA and GEV) went through the combined document to reach a consensus on annotation. After consensus was reached, one investigator (HMA) transferred the data (time stamp, data extract, annotated text) to an Excel file.

The nine hours of focus group discussions yielded a large number of potential issues for normalisation (n = 911). To reduce the size of the task, two investigators (PW and GEV) pre-analysed the transcript annotations, independently answering for each annotated issue “Are you sure this is about bias?” and “Are you sure this is a new item?” Issues for which both investigators answered yes to both questions were advanced directly to issue analysis. When investigators disagreed on either question, they discussed their responses to come to consensus on whether an item ought to be excluded from or advanced to issue analysis.

The next stage of issue analysis was conducted as an iterative process whereby two investigators (GEV and PW) independently proposed their interpretation of the core bias issues represented by an abstracted criterion or discussion point. Sometimes this involved splitting complex criteria or discussion points into multiple individual concepts. The investigators then compared their interpretations and reconciled differences through discussion. This process resulted in a list of draft items for the item bank.

The draft items often had redundant wording or otherwise lacked linguistic precision. These items were therefore reformulated a final time to remove unnecessary phrasing and eliminate as many trivial linguistic differences as was possible. The reformulations were proposed by PW and commented on by GEV, with disagreements about appropriate formulation discussed by the two investigators until consensus was reached. This process resulted in the final “normalised” set of items that constitute the item bank.

#### 3.3.1. Domain mapping

Each item was categorised according to the single best-fitting bias domains relating to the item. Categorisation decisions were made by one investigator (PW), with another (GEV) checking for agreement. Disagreements were resolved by discussion. The bias domains and definitions were taken from the Scientific Evidence Code System (SEVCO) research methods ontology (Alper et al. 2021b; FEvIR Platform Version 0.80.0 0.80.0, 2022).

The list of domains and their definitions and identifiers are shown in Table 2.

We chose the SEVCO bias domains because the grounding and consensus process used for developing their definitions (Alper et al. 2021a) makes them, in our opinion, the most comprehensive and robust available to us at the time of the conduct of our work. We modified one domain (“Timing of Study Termination Bias” SEVCO:00370, originally “Early study termination bias”) to reflect potential for bias from allowing an *in vitro* study to run on longer than originally planned, as well as from being stopped earlier than planned. We added two domains to support classification and analysis (“Metabias” and “Other”) to capture items that did not fit under the other bias domains (“metabias” was used for items that appear to affect multiple bias domains; “other” was for items that did not appear to relate to any bias domain). These additional domains are not intended to represent “true” biases. Four SEVCO domains (Mixed Methods Research Bias; Predictive Model Research Bias; Qualitative Research Bias; Synthesis Bias) ended up with no items and were therefore unused in the item bank.

### 3.4. Deviations from protocol

We made the following changes to the methods described in the original protocol (Svendsen et al. 2023):

- We added the Risk of Bias in Non-Randomized Follow-up Studies of Exposure Effects (ROBINS-E) tool (Higgins et al. 2024) to the set of literature sources, as we judged it could be important in broadening the scope of the issues covered by the item bank.
- We excluded from our literature sources the systematic review conducted by Tran et al. (2021) because it did not present in its results or supplemental materials a list of issues or criteria applied by the appraisal tools.
- The modification of “Early termination bias” and adding of two additional bias domains was not planned.
- We added the first-pass step to analysis of issues from the focus group discussions, to make it feasible to analyse an unexpectedly large number of potential items.

## 4. Results

405 unique bias items were included in the item bank. We abstracted from our included literature sources and focus group discussions 414 appraisal criteria and discussion points of plausible relevance to the internal validity of *in vitro* studies. After normalisation, this yielded 405 unique items in our item bank. Because some items occur in more than one source, our eight sources contributed 497 items in total.

The item bank is available in .xlsx format (Microsoft Excel) in Supplemental Materials 5. This file consists of seven sheets, as follows:

- Sheet 0 “Information.” The information sheet that provides guidance on the contents of the item bank file.
- Sheet 1 “Item Bank.” The item bank, consisting of the reference numbers, unique items (n = 405), and the bias domains under which each item has been categorised. Each item has a unique reference number to facilitate identification and tracking through the analysis process.
- Sheet 2 “Items by Source.” The item bank, including the source and original criterion or discussion point from which each item was derived. Because some items appear in multiple sources, the total number of records in this sheet (n = 497) is higher than the total number of items (n = 405).
- Sheet 3 “Abstracted Criteria.” The full list of criteria and discussion points abstracted from the literature sources and focus group discussions, that were interpreted into the items.
- Sheet 4 “Included Criteria.” The criteria and discussion points containing concepts that ended up being interpreted into items and included in the item bank. This sheet shows how the criteria and discussion points were interpreted into items by the investigators.
- Sheet 5 “Excluded Criteria.” The criteria and discussion points that were not interpreted into items in the item bank.
- Sheet 6 “Lookups.” The source for the drop-down lists of bias items, designed to reduce data input errors when creating the item bank.

Sheets 1 and 2 have been set up with filters to assist with exploring the item bank, allowing e.g. only items associated with analysis bias to be displayed (sheet 1), or only items associated with a specific source (sheet 2). The sheets are locked but not password protected to prevent accidental editing. To filter the data in the sheets, the user should right-click the tab for the sheet they are interested in filtering and select “Pause Protection.”

**Figure 1.**
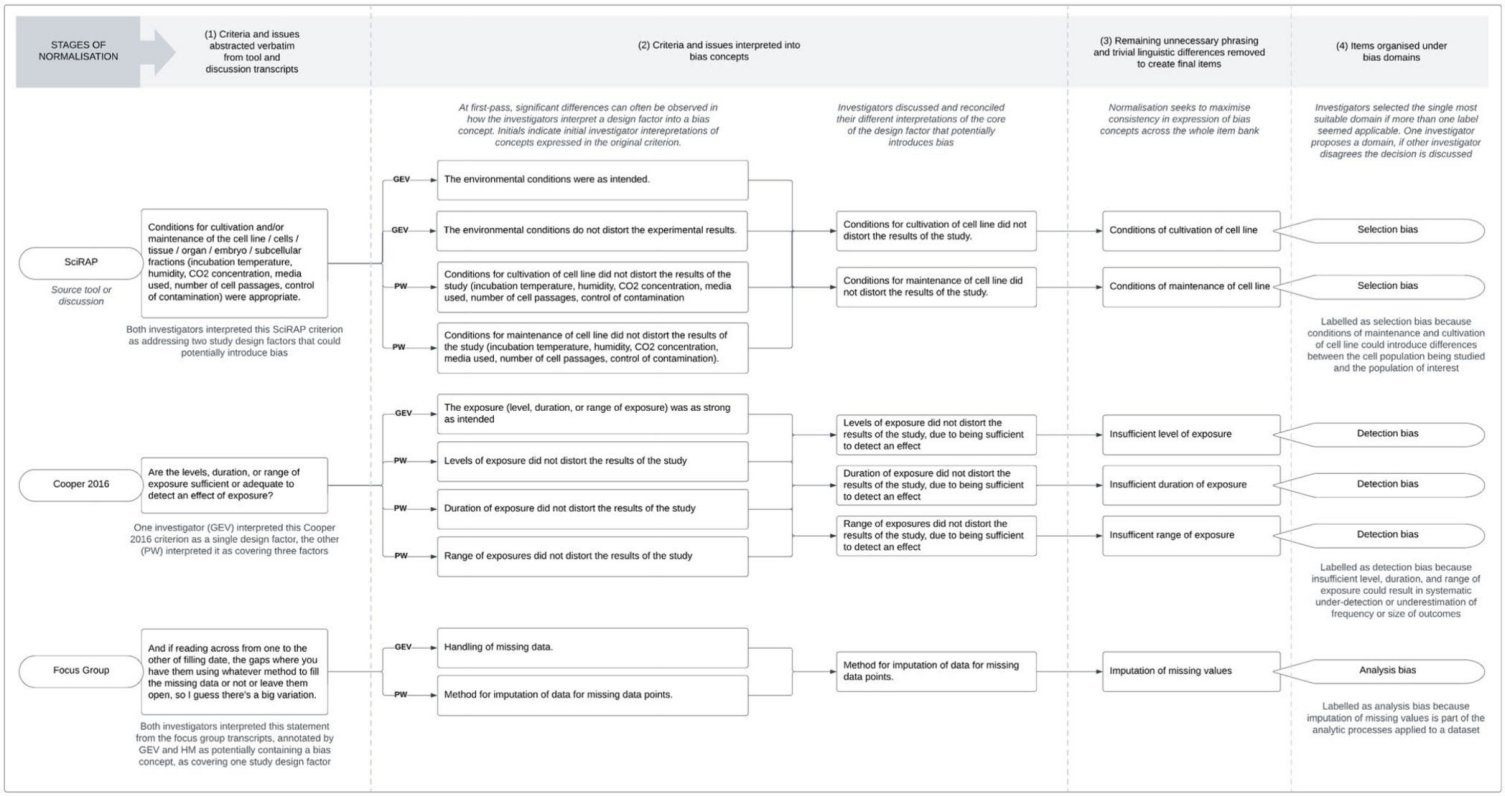
Flow chart of the process for analysing bias issues discovered in appraisal tools and focus group discussions. The flow chart shows how issues and appraisal tool criteria are normalised to create item bank items, with illustrative examples from three criteria sources (two tools and the focus group discussions). The flow chart shows progression from raw criterion, through interpretation into core concepts, normalisation for linguistic consistency, and the classification of each item under the most relevant bias domain.

**Figure 2.**
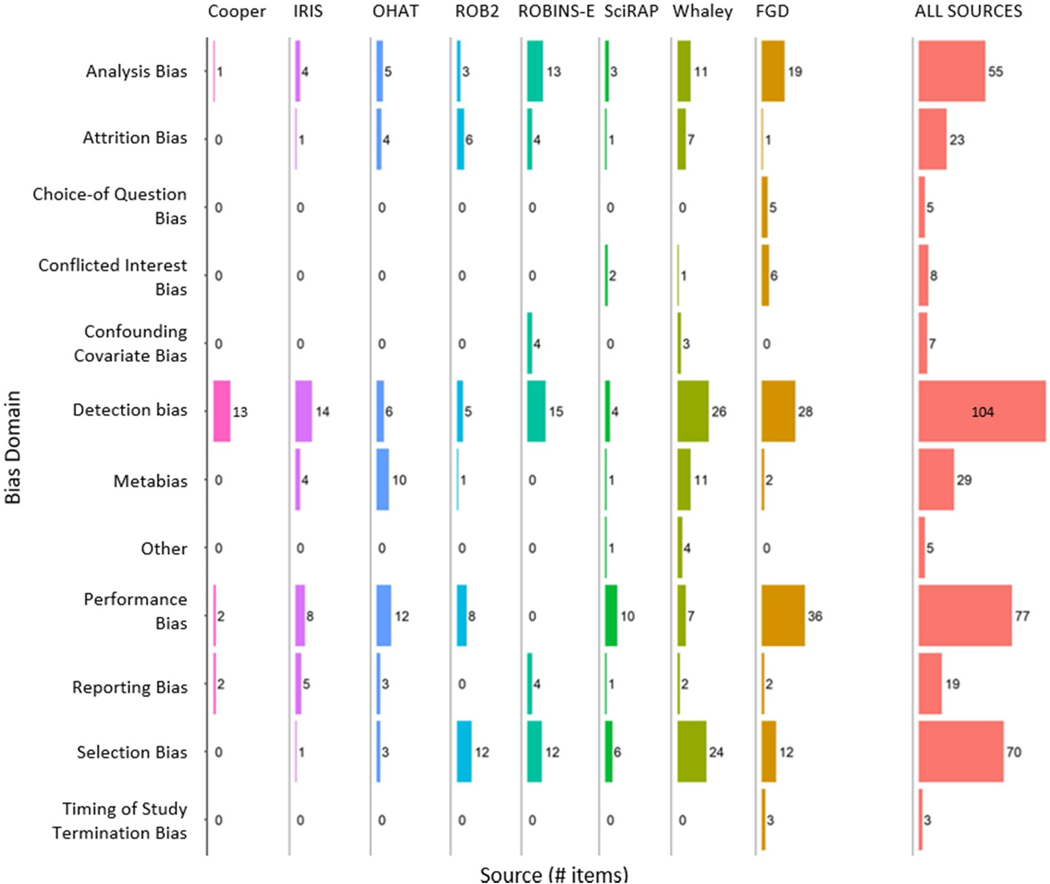
Unique bias items, disaggregated by source and in total from all sources. Note that the total count of items from all sources (column: ALL SOURCES) is lower than the sum of items from individual sources (columns: Cooper, IRIS, etc.) because some items appear in more than one source and are deduplicated in the final count. We used ggplot2 (Wickham 2016), ggpubr (Kassambara, 2023), fitdistrplus (Delignette-Muller and Dutang 2015), openxlsx (Schauberger and Walker 2024), and tidyverse (Wickham et al. 2019), to generate Figure 1. Code for generating the figure is available in Supplemental Material 6.

The relative contribution of items from different sources separated by bias domains is shown in Figure 1.

Detection bias (n = 104), performance bias (n = 77), and selection bias (n =70) were the domains with the most items. Two domains (choice-of-question bias and timing of study termination bias) did not have any related items in the literature sources, with items coming exclusively from focus group discussions (n =5 and n=3 respectively).

Items relating to confounding covariate bias (n = 7) were contributed by tools designed for assessing the internal validity of epidemiological studies. These were considered by the focus groups as potentially important for assessing the internal validity of *in vitro* studies that use primary biological samples; however, as this type of study is outside the scope of INVITES-IN at this time, this issue was not discussed in detail and the focus groups added no further items for this domain.

Only three items relating to conflicted interest bias were derived from the literature sources. The focus group discussions contributed six items, to give eight items in total under this domain after removal of duplicates. Conflicted interest bias was considered important by the focus groups, with several examples presented of where participants were concerned that this had impacted studies; however, the groups found it challenging to identify specific criteria for how conflict of interest could be assessed independently of the impact it has on decisions about how to design, conduct, or report a study.

Metabias is a very heterogeneous domain. It includes issues such as “blinding of operator” (item 299) that are both highly specific study design issues yet impact multiple bias domains. Importantly, the metabias domain includes “inappropriate deviation from protocol” (item 4048). We believe is the only reference in the whole item bank to the potentially important issue of inappropriate exploitation of researcher degrees of freedom (Wicherts et al. 2016) when conducting and reporting research.

The two literature sources we included that were not originally designed for *in vitro* studies (ROB2 for human randomised controlled trials and ROBINS-E for human observational studies of environmental exposures) contributed 24% of the items in the item bank (118 of 497 items before deduplication). The focus group discussions contributed 28% of the items in the item bank (139 of 497 items before deduplication).

## 5. Discussion

We have created a comprehensive bank of 405 items of potential relevance for the assessment of the internal validity of *in vitro* toxicology studies. We hope this provides a valuable dataset for informing the development of tools that include assessment of the internal validity of *in vitro* studies, and a methodology that is useful for appraisal tool development in general. Through our normalisation process we believe we advance on the methods recommended by Whiting et al. (2017) and used by Page et al. (2020). By conducting focus group discussions we believe we identified significantly more issues than we would have done through following an exclusively literature-based approach such as that of Whaley et al. (2024).

To support the interpretation and use of our item bank in tool development, and the reuse of our method, we discuss the following below:

- Our normalisation and exclusion decisions
- The importance of focus group discussions
- Some observations about the tools we included as literature sources
- Guidance on how to use the item bank

### 5.1. Normalisation and exclusion decisions

Our process for analysing criteria from literature sources and discussion points from the focus group discussions into normalised items was naturally subjective and completed in finite time. We expect that additional rounds of analysis would have eliminated further trivial differences between items and reduced the number of items overall (items 202 “exposure misclassification” and 208 “misclassification of exposure” are a particularly obvious example of where we missed conceptual duplicates).

Other researchers could reasonably argue that items such as 105 “storage of test compound” and 2097 “storage of test materials” are functionally equivalent and preserving the difference unhelpfully inflates the number of items in the item bank.

Our counterargument is we felt that preserving even small conceptual differences might be significant to the development of an INVITES-IN appraisal tool in a way we cannot anticipate during the item identification process. We felt that if we normalised away subtle differences, we risked losing important nuance and context that we would not be able to retrieve later. This could have a particular impact on the more detailed prompts, elicitation questions, and guidance that will form part of the eventual INVITES-IN tool. We therefore normalised items in such a way as to preserve detail, and it is in the post-Delphi stage of INVITES-IN (when we create the beta version of our tool) that we will remove detail in the items that is not helpful for our context.

Our exclusion decisions were similarly subjective. Other researchers may not agree with them, and it is possible we excluded concepts that should have been part of our final item bank. A limitation of our method is that we did not document all reasons for our exclusions (see Supplemental Materials 6, sheet 5) as it was too time-intensive to give informative reasons for hundreds of exclusion decisions based on sometimes lengthy discussion. In general, we would note that using a larger team of researchers to make exclusion and normalisation decisions, and conducting more rounds of normalisation, would lead to improved results. However, with hundreds of items and each round taking many person hours (we estimate two people each need 20 hours of discussion for 400 items), a decision must be made as to the cost-benefit trade-off of running each round of analysis.

To compensate for limitations and potential differences of opinion around our approach and resulting items, we have included in our item bank all the uninterpreted source criteria and discussion points, to give other teams all the materials they need to reinterpret our data.

It should also be noted that this study was a discovery exercise with the sole aim of collecting items for the creation of our item bank. Any reinterpretation of original tools and criteria for our specific purposes should not be interpreted as critique of those tools: it is a linguistic process of simplification that removes detail and information about context, to make it easier to identify and analyse as many as possible of the core internal validity concepts being expressed by the tools in aggregate.

### 5.2. The importance of focus groups and including non in vitro tools

In our opinion, including expert opinion in the preparation of the item bank via focus group discussions was very effective. The focus group discussions provided new insight into how participants considered, contextualised, and defined the different aspects of bias, significantly extending the range of issues that would have been covered had we focused exclusively on the literature for our item discovery process. The focus group discussions added a large number of items to analysis, detection, performance, and selection bias, with the practical experience of the participants able to add many considerations to bias domains of which the literature only had partial coverage.

It is important to note that the first focus group contributed 74 new discussion items, the second 30, and the third group 28. The fact that a large proportion of new items were still being discovered in the third focus group discussion suggests that there are more items yet to be discovered. Therefore, coverage of all critical aspects for the assessment of internal validity in *in vitro* studies, even in our item bank, remains incomplete and additional focus groups would still be of high value.

Our assumption that tools not designed for *in vitro* studies would be useful for *in vitro* contexts was also validated by our results, with ROB2 and ROBINS-E contributing many items that we otherwise would have missed had we excluded them.

### 5.3. Observations about included tools

While interpreting assessment criteria into items, we noted three issues that may be informative for the future development of appraisal tools in general.

#### 5.3.1. Multiple issues covered by single criteria

Firstly, there is a tendency for tools to collapse multiple points of evaluation into a single criterion, in a way that may be problematic. For example, the uninterpreted criterion number 126 (see Supplemental Materials 5, sheet 2 – reference numbers are in column B) states: “Was the exposure period, timing (i.e., cell passage number, insufficient culture maturity for the adequate expression of mature cell markers; insufficient treatment or measurement duration for the production of protein above the level of detection), frequency, and duration of exposure sensitive for the assay/model system of interest, particularly in the absence of a positive control?” This combines multiple aspects of exposure and use of positive controls into a single prompt. The uninterpreted criterion number 130, from the same tool, states: “Are there concerns regarding the need for positive controls (e.g., concerns that the effects of interest may be inhibited or otherwise poorly manifest in the test system, for example due to differences from in vivo biology)? If used, was the selected positive test substance (and dose) reasonable and appropriate and was the intended positive response induced?” This criterion covers several additional aspects of the use of positive controls, relating to the ability of a test system to manifest an outcome of interest.

Combining multiple issues into a single evaluation criterion may not always be problematic, for example when a criterion addresses two issues in a similar enough domain that it may not make sense to divide them in a particular study assessment context. However, on some occasions it may be important to separate these issues, (a) if this could affect the transparency or repeatability of the appraisal process, (b) it could affect reuse of data from the assessment when it is ambiguous what specific issue a judgement relates to, or (c) if it leads to double-counting of issues when there is overlap of common issues across two or more compound criteria.

#### 5.3.2. “Study sensitivity” as an assessment domain

Secondly, some study appraisal tools are concerned with the “sensitivity” of study methods (e.g. IRIS 2022, Cooper et al. 2016). However, when mapping items derived from sensitivity concerns onto bias domains, many of these items seemed to fit under the domain of detection bias (e.g. the items “Insufficient level of exposure” (ref 53), “Insufficient duration of exposure” (ref 54) “Insufficient range of doses” (ref 69), “Duration of exposure” (ref 72)). Others seemed to fit under the performance bias domain (e.g. the items “Method of administration of exposure” (ref 73), “Route of administration of exposure” (ref 74)) or the reporting bias domain (e.g. the item “Choice of analyses reported” (ref 76)). Some sensitivity criteria were excluded as relating to the precision of a study (e.g., the uninterpreted criterion “Are the numbers of exposed cases adequate to detect an effect in the exposed population and/or subgroups of the exposed population?” (ref 50)) or the external validity of a study (e.g. the criterion “Are the outcome ascertainment methods reliable and valid; is the outcome under study a sensitive indicator of the health effect of interest?” (ref 51)).

While it is not necessarily important how issues relating to potential systematic error are divided into assessment domains, having the same concept split across two or more separate domains may add unnecessary complexity to an assessment process and potentially increase the risk of double-counting appraisal factors. For example, the criteria “Does the exposure assessment capture the relevant window of exposure?” (ref 57) and “Is the timing and duration of exposure relevant for the endpoints under study? If the sensitive exposure window is unknown, is the exposure period wide enough to cover likely possibilities?” (ref 71) seem closely related but are different questions in the source tool. Similarly, the criteria “Variable Control: Are all introduced variables with the potential to affect the results of interest controlled for and consistent across experimental groups?” (ref 85) and “Are appropriate control groups for the study/assay type included? Was there a need for the assay to include specific controls to reduce potential sources of underlying bias?” (ref 113) appear to be the covering aspects of the same issue twice in the same tool.

This observation should not be interpreted as a critique of the specific assessment tools, as we are only making general observations across a dataset and not analysing specific tools in detail. However, it may be worthwhile conducting a closer inspection and re-evaluation of the principles and structure behind some study assessment processes, and its potential impact on the validity of the results of the evaluation process using these tools.

#### 5.3.3. Existing tools lag expert knowledge

Thirdly, the coverage of all critical aspects for the assessment of internal validity by existing tools may not be optimal. The focus group discussions identified many new issues and were the single-largest contributor of assessment items, including items to some domains that were completely unmentioned in the existing tools that we sampled. *In vitro* study methods are a fast-evolving field and any appraisal tool for the domain will likely need regular updating to keep up with ongoing developments.

### 5.4. Guidance on how to use the item bank

The item bank is a database of concepts and requires further analysis when creating an appraisal tool – it is like an ingredient for a dish which cannot be consumed directly off the shelf. For the beta version of INVITES-IN we are using Computer-Assisted Qualitative Data Analysis Software (CAQDAS) to identify clusters of related concepts under a range of possible appraisal themes that we could potentially use for structuring our appraisal tool. We expect the broad themes to provide structure in the form of domains or sections of the tool; for similarities between clusters of related items to be interpretable into elicitation questions; and for the more granular details of the items to inform specific prompts or detailed guidance on how elicitation questions in the tool should be answered.

In this process, we are finding it helpful to preface an item with a phrase that prompts reflection on how the concept being expressed could result in issues with the internal validity of a study. For example, for the item “Conditions of maintenance of cell line” we might think “Problems with conditions of maintenance of cell line that introduce bias” to keep us focused on interpreting the item for our task. A different team using our item bank to create a reporting checklist might prefer to interpret items for their context as follows: “Problems with reporting the conditions of maintenance of the cell line that compromise the reproducibility of the study.” Our item bank is intended as a general resource, which means the user must interpret the items for their specific context.

## 6. Conclusion

We have created a comprehensive item bank of 405 unique bias items that may be relevant for assessing the internal validity of *in vitro* studies. The inclusion of focus group discussions with *in vitro* experts was of significant value in our method, adding more than a hundred additional bias items and enhancing our understanding of the challenges associated with assessing the internal validity of *in vitro* studies. We believe that both our method used to create the comprehensive item bank, and the item bank itself, will be useful for other researchers creating validity assessment tools. Overall, we believe our item bank to be a valuable dataset of bias concepts that can support the development of tools for the assessment of *in vitro* studies in the field of toxicology, that may also be useful for appraisal tools for other fields and study designs.

### Project governance

This study is part of the project “Next Generation Risk Assessment in Practice” (VKM 2023), included in the European Partnership for the Assessment of Risks from Chemicals (PARC) (PARC 2023). A project group is responsible for the creation of INVITES-IN. A scientific advisory group consisting of experts in methods for tool development, systematic review methods, chemical risk assessment, toxicology, and/or *in vitro* models has been established to provide the project group with strategic guidance and support during the process of creating INVITES-IN (VKM 2023).

## Supplementary Material

Supplements

## Figures and Tables

**Table 1.** Literature sources for item bank.

Source ID	Source name or title	Source type	Source citation
IRIS	IRIS: ORD Staff Handbook for Developing IRIS Assessments	Guidance on study appraisal for IRIS assessments	EPA (2022)
OHAT	OHAT: Protocol to evaluate the evidence for an association between perfluorooctanic acid (PFOA) and perfluorooctane sulfonate (PFOS) exposure and immunotoxicity	Bias assessment tool	NTP OHAT (2015)
ROB2	ROB 2: A revised tool for assessing risk of bias in randomised trials	Bias assessment tool	Sterne et al. (2019)
ROBINS-E	ROBINS-E: Risk Of Bias In Non-randomized Studies - of Exposure	Bias assessment tool	Higgins et al. (2024)
SciRAP	SciRAP: Development of the SciRAP Approach for Evaluating the Reliability and Relevance of *in vitro* Toxicity Data	Study appraisal tool for supporting risk assessment	Roth, Zilliacus, and Beronius (2021)
Cooper	Study sensitivity: Evaluating the ability to detect effects in systematic reviews of chemical exposures	Guidance on assessing study sensitivity	Cooper et al. (2016)
Whaley	Literature-based discovery of assessment criteria for *in vitro* studies: a method and item bank	Item bank	Whaley, Hooijmans, and Wattam (2023)

**Table 2.** Bias domains used for classifying item bank items.

Bias domain	Definition	Source	Identifier
Analysis bias	A bias related to the analytic process applied to the data.	SEVCO	SEVCO:00021
Attrition bias	A bias due to absence of expected participation or data collection after selection for study inclusion.	SEVCO	SEVCO:00019
Choice-of-question bias	A bias in research design in which the research question (that the study is designed to answer) is inappropriate for the context.	SEVCO	SEVCO:00018
Conflicted interests bias	A bias in which decision makers influencing research design, conduct, analysis or reporting have goals or motivations that conflict with scientific research objectives.	SEVCO	SEVCO:00027
Confounding covariate bias	A situation in which the effect or association between an exposure or outcome is distorted by another variable. For confounding covariate bias to occur the distorting variable must be (1) associated with the exposure and the outcome, (2) not in the causal pathway between exposure and outcome, and (3) unequally distributed between the groups being compared.	SEVCO	SEVCO:00016
Detection bias	A bias due to distortions in any process involved in the determination of the recorded values for a variable.	SEVCO	SEVCO:00020
Timing of study termination bias*	A bias due to the decision to end the study earlier or later than planned. *Modified from the original SEVCO term “early study termination bias,” defined as “a bias due to the decision to end the study earlier than planned”	SEVCO	SEVCO:00370*
Performance bias	A bias resulting from differences between the received exposure and the intended exposure.	SEVCO	SEVCO:00017
Reporting bias	A bias due to distortions in the selection of or representation of information in study results or research findings.	SEVCO	SEVCO:00023
Selection bias	A bias resulting from methods used to select subjects or data, factors that influence initial study participation, or differences between the study sample and the population of interest	SEVCO	SEVCO:00002
Metabias	A general distorting influence on investigator decision-making that may bias the results or findings of a study	Added by project team	INVITE:00003
Other	A distortion in results due to factors other than those described in the SEVCO terms	Added by project team	INVITE:00001

## Abbreviations:

FGD: focus group discussion
SEVCO: Scientific Evidence Code System

## Definitions/Glossary

Core term definitions have been updated since publication of our study protocol (Svendsen et al. 2023).

Bias: is a systematic error, or deviation from the truth, in the results or findings of individual studies or across studies. Systematic error may be introduced through limitations in the design, conduct, or reporting of a study
Bias domains: are a conceptual framework for organizing the limitations in design, conduct, or reporting of a study that affect the internal validity of a study. Bias domains in the present work include but are not limited to detection, performance, and selection bias
Criteria: are the conditions that have to be fulfilled for bias to be avoided. In tools for evaluation of risk of bias, the users of a tool will often answer signalling questions in order to determine whether the bias criteria have been met
Internal validity: is the extent to which the design, conduct, and reporting of a study is likely to have prevented bias
Items: are linguistically minimalist expressions of study assessment concepts. In the present work, “items” are derived by a normalisation process from assessment criteria presented in study appraisal tools and described in focus group discussions, that potentially relate to the internal validity of an in vitro study
Item bank: is a database of items
Normalisation: is the process of organizing data in a database. In the present work, this involves identifying and consistently expressing the core bias concepts underlying the assessment criteria applied by study appraisal tools
Risk of bias: is the potential for systematic error in the results or findings of a study or across studies. Tools for assessing the internal validity of studies tend to focus on risk of bias, as bias itself cannot usually be directly measured
Signalling questions: are the questions that the users of a validity assessment tool answer in order to determine whether a given set of assessment criteria have been fulfilled
