# Supplementary material for: A comprehensive item bank of internal validity issues of relevance to in vitro toxicology studies
Source: Evid Based Toxicol. Author manuscript; Available in PMC 2025 Oct 31. (PMC12180937; doi:10.1080/2833373X.2024.2418045)
Supplement: Supplements [file NIHMS2054894-supplement-Supplements.zip › Supplemental Material 1_Item Bank_Invitation letter to focus group participants_R1.docx]

**An internal validity item bank including items of relevance for *in vitro* studies**

# Supplementary Materials 1

## Invitation to participate in the focus group study

Dear X,

We are writing to invite you to take part in a Focus Group study, which is the first of three studies that will be performed to create a tool for evaluation of internal validity of *in vitro* studies.

Before you decide whether or not you would like to take part, it is important for you to consider why the research is being done and what it will involve. Please read the following information carefully.

**What is the purpose of the project?**

*In vitro* studies are becoming an increasingly important source of data in risk assessment of chemicals, in the process to reduce the use of animals in toxicity testing. As part of the gradual incorporation and transition toward the use of new approach methodologies (NAMs), a framework for evidence-based use of NAMs in toxicological research and chemical risk assessment is required. With our project, we aim to contribute to the development of such a framework for evidence-based use of NAMs in human health hazard identification and characterisation. The first step is the development of a tool for evaluation of internal validity of *in vitro* studies. Internal validity is the extent to which the design and conduct of a study are likely to have prevented bias. Bias are systematic errors resulting in deviations from the truth in results or inference, and for *in vitro* studies such errors may be introduced in the study design, conduction, and/or analysis, and cause the result to be an overestimate or underestimate.

The tool will be useful for inclusion of NAMs in systematic literature reviews and risk assessments.

**What is the purpose of the focus group study?**

The objective of the focus group study is to have expert interpretation of the relevance of different characteristics of study design, conduct, and analysis for introduction of bias into the results or inference of *in vitro* studies.

**How are the Focus Group meetings organised?**

We will have three different focus groups with a group size of six to eight participants.

All participants in a focus group will be selected from different institutions in an attempt to achieve variation in input, and they should be working with a variety of *in vitro* models to cover a wide range of experimental systems. We also aim to include participants affiliated in the academia, governmental, and private research institutions, that are from a range of countries, and to have a gender balance.

Dr Paul Whaley will act as a focus group moderator and lead the discussions in the meeting, and Dr Gunn Vist will act as assistant moderator. In the meeting, focus group participants will be led into discussion of how different bias might be of relevance for the *in vitro* context.

Each focus group will have one meeting lasting about 90 minutes.

**Why have you been invited to take part?**

We are looking to recruit 18-24 participants with a balanced mix of experience. To be eligible to participate all invited participants must confirm that they fulfil the following criteria:

- Active in the field of *in vitro* research in academia, governmental institution (including risk assessment institutions and research institutes) or private research institution
- At post-doctoral level or higher
- Fluent English speaker

As an established expert who fulfils the above criteria, we are keen to gain your views about the relevance of different characteristics of study design, conduct, and analysis for introduction of bias into the results or findings of *in vitro* studies.

**What will you be asked to do if you take part?**

If you agree to be a participant in this Focus Group study, you will need to tell us:

- That you fulfil the eligibility criteria
- That you are available and willing to participate in one Focus Group meeting
- If you believe your involvement in this project might present any potential conflicts of interest

As an accepted participant in this Focus Group study, you must not discuss your involvement, or any of the information shared with you during the project with anyone other than the research team.

The Focus Group meeting will be arranged in March this year.

**Withdrawal procedure**

Participants are free to request their withdrawal from the Focus Group study at any point. Participants are asked to communicate their withdrawal request via email to Dr Gro Haarklou Mathisen. The request should state whether the withdrawal is because of:

- A personal issue
- A professional conflict with the study

No further information regarding reasons for the withdrawal will be requested.

**Who is organizing and funding the research?**

The project is led by the Norwegian Institute of Public Health represented by the Norwegian Scientific Committee for Food and Environment, and the project lead is Dr Gro Haarklou Mathisen. The project is part of the work in the European Partnership for the Assessment of Risks from Chemicals (PARC) project “Next Generation Risk Assessment in Practice”.

The project receives funding from PARC HORIZON-HLTH-2021-ENVHLTH-03, Grant [101057014].

**Confidentiality**

The name and contact email address of Focus Group participants will be known only by the core research team involved in this project (five persons) and used for the purpose of facilitating direct communication regarding the Focus Group. The Focus Group will not require personal information to be entered and therefore no personal information will be collected. All responses received will be strictly confidential, and only anonymised summary data will be shared.

**Data protection**

The Focus Group discussion will be carried out as an online meeting and will be recorded. Transcripts of the focus group discussions will be machine-generated. Anonymised transcripts will be shared as raw data.

Data will be stored for the duration of the research project only and then deleted. Anonymised summary data will be shared with the project team and participants after the Focus Group discussions. Following completion of this study, only anonymised summary data will be kept and shared via an Open Science Framework platform on a journal website in accordance with the journals publication policy requirements.

**Research ethics**

The proposed Focus Group study abides by the ethical requirements of the Norwegian Institute of Public Health, approved February 2023.

**What do you do now?**

Thank you for reading this information and for considering taking part in this research. Please let us know whether or not you would like to be a participant by replying to this email. If you wish to participate, we also require you to complete and return the attached consent form. If you have any questions or concerns, please do not hesitate to contact me.

Best Wishes,

Gro Haarklou Mathisen, on behalf of the research team
