# Supplementary material for: A comprehensive item bank of internal validity issues of relevance to in vitro toxicology studies
Source: Evid Based Toxicol. Author manuscript; Available in PMC 2025 Oct 31. (PMC12180937; doi:10.1080/2833373X.2024.2418045)
Supplement: Supplements [file NIHMS2054894-supplement-Supplements.zip › Supplemental Material 2_Item Bank_Focus group participant characteristics _R1.docx]

**An internal validity item bank including items of relevance for *in vitro* studies**

# Supplementary Materials 2

Focus group participant characteristics

| **Country of residence** | Belgium; Canada; Germany; Italy; Netherlands; Norway; Sweden; United States of America |
| --- | --- |
| **Main employer** | Center for Alternatives to Animal Testing - Europe  European Commission Joint Research Centre  Freelance medical writer and consultant  German Federal Institute for Risk Assessment  Institute for In Vitro Sciences  Institute of Marine Research  Konstanz University  Karolinska Institutet  Leibniz Research Institute for Environmental Medicine  National Institute of Occupational Health  Norwegian Institute of Public Health  Procter & Gamble Services NV  National Institute for Health and the Environment  Self-employed  TEAM mastery S.r.l.  The Climate and Environmental Research Institute  United States Environmental Protection Agency  University of California Irvine  University of Ottawa |
| **Gender** | Female: 15; Male: 5 |
| **Years of experience with *in vitro* studies** | No experience: 1*  1-3 years: 0  4-6 years: 2  More than 6 years: 17 |
| **Years of experience with chemical risk assessment** | No experience: 2  1-3 years: 3  4-6 years: 0  More than 6 years: 15 |
| **Years of experience with systematic reviews** | No experience: 5  Some experience (*e.g. been involved in one systematic review or peer-reviewed several systematic reviews*): 8  Moderate experience (*conducted study appraisal in at least one systematic review*): 4  Extensive experience (*have designed the methods, including selection, modification, or developed study assessment methods, for at least one systematic review*): 3 |

*One person didn't have hands on experience doing *in vitro* research and reported 0 years of experience. However, this person is, and have been, involved in much relevant work on *in vitro* studies such as e.g. development of adverse outcome pathways, so we still found that this person had valuable *in vitro* expertise.
