# Supplementary material for: A comprehensive item bank of internal validity issues of relevance to in vitro toxicology studies
Source: Evid Based Toxicol. Author manuscript; Available in PMC 2025 Oct 31. (PMC12180937; doi:10.1080/2833373X.2024.2418045)
Supplement: Supplements [file NIHMS2054894-supplement-Supplements.zip › Supplemental Material 3_Item Bank_Slides used in the focus group discussion meetings_R1.pdf]

INVITES-IN Focus Group

Group 1, Meeting 1 :: 12 June 2023

What we are doing and how

We are developing INVITES-IN, a tool for assessing the internal validity (potential for systematic error in results or findings) of a study. The tool will be used in systematic reviews.

We are collecting potential bias criteria from existing study assessment tools (literature review) and expert opinion (focus groups)

- Goal of focus groups is to discover more bias items than we captured from the literature (n≈250, 72 assessment tools)
- Objective of focus groups is to gather more data, not secure agreement (agreement process is separate)
- No ideas are wrong: brainstorming sessions where we will try to move along discussion on 10 bias domains

Bias domains

1. Analysis Bias
2. Attrition Bias
3. Choice-of-Question Bias
4. Conflicted Interests Bias
5. Confounding Covariate Bias
6. Detection Bias
7. Early Study Termination Bias
8. Performance Bias
9. Reporting Bias
10. Selection Bias

Structure of the discussion

1. Show you the domain and the number of criteria we have discovered
2. Show you the definition of the domain, discuss what it means to you for the in vitro context
3. Provide some examples of criteria we have discovered, to help discussion
4. Provide some examples of criteria that we have found challenging to interpret, for you to comment on specifically

We have chosen which domains and terms are discussed based on previous focus group discussions. Aiming for comprehensiveness.

Domain choices are to help structure discussion. We are not discussing whether the domains are an appropriate or complete classification scheme for biases

Administrative information

- This session is being recorded and machine-transcribed
- Data will be anonymised. Participants should not at a later date attribute comments to specific individuals.
- Facilitators are Paul Whaley (lead) and Gunn Vist (steer)
- Note takers is Heather Ames
- Gro Mathison is the project supervisor and lead (not present)
- If you have any questions or concerns about process, please raise them now

Analysis Bias ($n \approx 32$)

Analysis Bias: Definition

A bias related to the analytic process applied to the data

Analysis Bias: Examples

A bias related to the analytic process applied to the data

- Masking analysis
- Correcting for or imputing missing data
- Data reduction, normalisation, standardisation, noise reduction
- Prior knowledge of data before developing analysis plan

Analysis Bias: Specific checks

A bias related to the analytic process applied to the data

- Absence from analysis of predictors of missing data
- Correcting for e.g. selection factors
- Controlling for baseline differences, confounders
- Controlling for time-varying factors
- Use of control data
- Use of software

Attrition Bias (n≈16)

Attrition Bias: Definition

A bias due to absence of expected **participation** or **data collection after selection** for study inclusion

Attrition Bias: Examples

A bias due to absence of expected **participation** or **data collection after selection** for study inclusion

- Exclusion of samples from analysis
- Incomplete data on exposure, outcome, confounders, missing data
- Loss of samples

Attrition Bias: Specific checks

A bias due to absence of expected **participation** or **data collection after selection** for study inclusion

- Cytotoxicity of test compound

Choice-of-Question Bias (n=0)

Choice-of-Question Bias

A bias in research design in which the research question (that the study is designed to answer) is inappropriate for the context.

- No examples, open discussion

Conflicted Interests Bias (n=2)

Conflicted Interests Bias: Definition

A bias in which decision makers influencing research design, conduct, analysis or reporting have goals or motivations that conflict with scientific research objectives

Conflicted Interests Bias: Examples

A bias in which decision makers influencing research design, conduct, analysis or reporting have goals or motivations that conflict with scientific research objectives

- Sources of funding
- Conflicts of interest

Conflicted Interests Bias: Specific checks

A bias in which decision makers influencing research design, conduct, analysis or reporting have goals or motivations that conflict with scientific research objectives

- **Mismanagement** of interests in decision-making

Confounding Covariate Bias (n≈24)

Confounding Covariate Bias

A situation in which the effect or association between an exposure or outcome is distorted by another variable

For confounding covariate bias to occur the distorting variable must be

1. associated with the exposure and the outcome,
2. not in the causal pathway between exposure and outcome, and
3. unequally distributed between the groups being compared.

Confounding Covariate Bias: Examples

A situation in which the effect or association between an exposure or outcome is distorted by another variable

- Method (if any) for random allocation to exposure groups
- Baseline differences between exposure groups

Confounding Covariate Bias: Specific checks

A situation in which the effect or association between an exposure or outcome is distorted by another variable

- Time-varying confounding (confounders that change value over time)
- Other ways in which there can be baseline differences between groups?

Detection Bias ($n \approx 80$)

Detection Bias: Definition

A bias due to distortions in any process involved in the determination of the recorded values for a variable

Detection Bias: Examples

A bias due to distortions in any process involved in the determination of the recorded values for a variable

- Detection of exposure
- Detection of outcome
- Use of appropriate comparison groups (e.g. controls, doses)
- Masking of investigators (outcome assessors)

Detection Bias: Specific checks

A bias due to distortions in any process involved in the determination of the recorded values for a variable

- Detection of confounders
- Timing of exposure: time-point, window, duration, latency periods
- Timing of outcome measurement
- Use of image analysis and data visualisation
- Equipment issues additional to levels of detection
- Marker cut-off points

Early Study Termination Bias (n=0)

Early Study Termination Bias: Definition

A bias due to the decision to end the study earlier than planned

- Ending a study early or late to generate more favourable results
- No examples

Performance Bias ($n \approx 44$)

Performance Bias: Definition

A bias resulting from differences between the received exposure and the intended exposure

Performance Bias: Examples

A bias resulting from differences between the received exposure and the intended exposure

- Culture conditions e.g. medium, maintenance
- Investigator knowledge of exposure group (exposure, maintenance)
- Error in test substance
- Impurities in test substance
- Solubility of test substance

Performance Bias: Specific checks

A bias resulting from differences between the received exposure and the intended exposure

- Breaking masking
  - failure of allocation concealment due to recognising patterns in numbering
  - visual differences between groups
- Different methods of exposure administration between groups
- Influence of vehicle
- Use of robotic test systems

Reporting Bias ($n \approx 15$)

Reporting Bias: Definition

A bias due to distortions in the selection of or representation of information in study results or research findings

Reporting Bias: Examples

A bias due to distortions in the selection of or representation of information in study results or research findings

- Choice of reported exposures
- Choice of reported outcomes
- Choice of reported analyses
- Choice of desired result from multiple analyses, subgroups

Reporting Bias: Specific checks

A bias due to distortions in the selection of or representation of information in study results or research findings

- Selective emphasis on post-hoc analysis

Selection Bias (n≈20)

Selection Bias: Definition

A bias resulting from: methods used to select subjects or data; factors that influence initial study participation; or differences between the study sample and the population of interest

Selection Bias: Examples

A bias resulting from: methods used to select subjects or data; factors that influence initial study participation; or differences between the study sample and the population of interest

Selection Bias: Specific focus

A bias resulting from: methods used to select subjects or data; factors that influence initial study participation; or differences between the study sample and the population of interest

Predictive Model Research Bias

Predictive Model Research Bias

A bias specific to the design, conduct, analysis or reporting of research about predictive modelling.

“Other”

Other: Definition

A distortion in results due to factors other than those described above.

Other: Examples

A distortion in results due to factors other than those described above.

- Choice of source of activation system, given test article
- Inherent physicochemical properties of test substance

Examples of “other”

Test methods (not specific, what does it mean in addition to the above factors?)

INVITES-IN Focus Group

Group 1, Meeting 2 :: 13 June 2023

Mic checks for everyone

What we are doing and how

We are developing INVITES-IN, a tool for assessing the internal validity (potential for systematic error in results or findings) of a study. The tool will be used in systematic reviews.

We are collecting potential bias criteria from existing study assessment tools (literature review) and expert opinion (focus groups)

- Goal of focus groups is to discover more bias items than we captured from the literature (n≈250, 72 assessment tools)
- Objective of focus groups is to gather more data, not secure agreement (agreement process is separate)
- No ideas are wrong: brainstorming sessions where we will try to move along discussion on 10 bias domains

Bias domains

1. ~~Analysis Bias~~
2. ~~Attrition Bias~~
3. ~~Choice-of-Question Bias~~
4. ~~Conflicted Interests Bias~~
5. ~~Confounding Covariate Bias~~
6. Detection Bias
7. Early Study Termination Bias
8. Performance Bias
9. **Predictive Model Research Bias**
10. Reporting Bias
11. Selection Bias
12. **“Other”**

Structure of the discussion

1. Show you the domain and the number of criteria we have discovered
2. Show you the definition of the domain, discuss what it means to you for the in vitro context
3. Provide some examples of criteria we have discovered, to help discussion
4. Provide some examples of criteria that we have found challenging to interpret, for you to comment on specifically

We have chosen which domains and terms are discussed based on previous focus group discussions. Aiming for comprehensiveness.

Domain choices are to help structure discussion. We are not discussing whether the domains are an appropriate or complete classification scheme for biases

Administrative information

- This session is being recorded and machine-transcribed
- Data will be anonymised. Participants should not at a later date attribute comments to specific individuals.
- Facilitators are Paul Whaley (lead) and Gunn Vist (steer)
- Note taker is Heather Ames
- Gro Mathison is the project supervisor and lead (not present)
- If you have any questions or concerns about process, please raise them now

Recording check

Detection Bias (n≈80)

Detection Bias: Definition

A bias due to distortions in any process involved in the determination of the recorded values for a variable

Detection Bias: Examples

A bias due to distortions in any process involved in the determination of the recorded values for a variable

- Detection of exposure
- Detection of outcome
- Use of appropriate comparison groups (e.g. controls, doses)
- Masking of investigators (outcome assessors)

Detection Bias: Specific checks

A bias due to distortions in any process involved in the determination of the recorded values for a variable

- Detection of confounders
- Timing of exposure: time-point, window, duration, latency periods
- Timing of outcome measurement
- Use of image analysis and data visualisation
- Equipment issues additional to levels of detection
- Marker cut-off points

Early Study Termination Bias (n=0)

Early Study Termination Bias: Definition

A bias due to the decision to end the study earlier than planned

- Ending a study early or late to generate more favourable results
- No examples

Performance Bias ($n \approx 44$)

Performance Bias: Definition

A bias resulting from differences between the received exposure and the intended exposure

Performance Bias: Examples

A bias resulting from differences between the received exposure and the intended exposure

- Culture conditions e.g. medium, maintenance
- Investigator knowledge of exposure group (exposure, maintenance)
- Error in test substance
- Impurities in test substance
- Solubility of test substance

Performance Bias: Specific checks

A bias resulting from differences between the received exposure and the intended exposure

- Breaking masking
    - failure of allocation concealment due to recognising patterns in numbering
    - visual differences between groups
- Different methods of exposure administration between groups
- Influence of vehicle
- Use of robotic test systems

Predictive Model Research Bias (n=0)

Predictive Model Research Bias

A bias specific to the design, conduct, analysis or reporting of research about predictive modelling

- No examples, open discussion

Reporting Bias (n≈15)

Reporting Bias: Definition

A bias due to distortions in the selection of or representation of information in study results or research findings

Reporting Bias: Examples

A bias due to distortions in the selection of or representation of information in study results or research findings

- Choice of reported exposures
- Choice of reported outcomes
- Choice of reported analyses
- Choice of desired result from multiple analyses, subgroups

Reporting Bias: Specific checks

A bias due to distortions in the selection of or representation of information in study results or research findings

- Selective emphasis on post-hoc analysis

Selection Bias ($n \approx 20$)

Selection Bias: Definition

A bias resulting from: methods used to select subjects or data; factors that influence initial study participation; or differences between the study sample and the population of interest

Selection Bias: Examples

A bias resulting from: methods used to select subjects or data; factors that influence initial study participation; or differences between the study sample and the population of interest

Selection Bias: Specific focus

A bias resulting from: methods used to select subjects or data; factors that influence initial study participation; or differences between the study sample and the population of interest

“Other”

Other: Definition

A distortion in results due to factors other than those described above.

Other: Examples

A distortion in results due to factors other than those described above.

- Choice of source of activation system, given test article
- Inherent physicochemical properties of test substance

INVITES-IN Focus Group

Group 2, Meeting 1 :: 19 June 2023

Mic checks for everyone

What we are doing and how

We are developing INVITES-IN, a tool for assessing the internal validity (potential for systematic error in results or findings) of a study. The tool will be used in systematic reviews.

We are collecting potential bias criteria from existing study assessment tools (literature review) and expert opinion (focus groups)

- Goal of focus groups is to discover more bias items than we captured from the literature (n≈250, 72 assessment tools)
- Objective of focus groups is to gather more data, not secure agreement (agreement process is separate)
- No ideas are wrong: brainstorming sessions where we will try to move along discussion on 12 bias domains

Bias domains (alphabetical)

1. Analysis Bias
2. Attrition Bias
3. Choice-of-Question Bias
4. Conflicted Interests Bias
5. Confounding Covariate Bias
6. Detection Bias
7. Early Study Termination Bias
8. Performance Bias
9. Predictive Model Research Bias
10. Reporting Bias
11. Selection Bias
12. “Other”

Structure of the discussion

1. Show you the domain being discussed and the number of criteria we have discovered
2. Show you the definition of the domain, ask what it means to you for the in vitro context
3. Provide some examples of criteria we have discovered, to help discussion
4. Provide some examples of criteria that we have found challenging to interpret, for you to comment on specifically

Notes on structure

- We have chosen which domains and terms are discussed based on previous focus group discussions. Aiming for comprehensiveness.
- Domain choices are to help structure discussion. We are not discussing whether the domains are an appropriate or complete classification scheme for biases.
- I will say some things that may sound naive or obvious. Do not be thrown by this, I will be trying to surface concepts without leading your responses.

Administrative information

- This session is being recorded and machine-transcribed
- Data will be anonymised. Participants should not at a later date attribute comments to specific individuals.
- Facilitators are Paul Whaley (lead) and Gunn Vist (steer)
- Note taker is Heather Ames
- Gro Mathison is the project supervisor and lead, and point of contact for any concerns about today’s facilitation process
- If you have any questions or concerns about process, please raise them now (**any questions?**)

Recording check (on, transcribing)

Detection Bias (n≈80)

Detection Bias: Definition

A bias due to distortions in any process involved in the determination of the recorded values for a variable

Detection Bias: Examples

A bias due to distortions in any process involved in the determination of the recorded values for a variable

- Detection of exposure
- Detection of outcome
- Use of appropriate comparison groups (e.g. controls, doses)
- Masking of investigators (outcome assessors)

Detection Bias: Specific checks

A bias due to distortions in any process involved in the determination of the recorded values for a variable

- Detection of confounders
- Timing of exposure: time-point, window, duration, latency periods
- Timing of outcome measurement
- Use of image analysis and data visualisation
- Equipment issues additional to levels of detection
- Marker cut-off points

Reporting Bias ($n \approx 15$)

Reporting Bias: Definition

A bias due to distortions in the selection of or representation of information in study results or research findings

Reporting Bias: Examples

A bias due to distortions in the selection of or representation of information in study results or research findings

- Choice of reported exposures
- Choice of reported outcomes
- Choice of reported analyses
- Choice of desired result from multiple analyses, subgroups

Reporting Bias: Specific checks

A bias due to distortions in the selection of or representation of information in study results or research findings

- Selective emphasis on post-hoc analysis

Selection Bias ($n \approx 20$)

Selection Bias: Definition

A bias resulting from: methods used to select subjects or data; factors that influence initial study participation; or differences between the study sample and the population of interest

Selection Bias: Examples

A bias resulting from: methods used to select subjects or data; factors that influence initial study participation; or differences between the study sample and the population of interest

- Cell line authentication
- Cell line contamination
- Conditions of cultivation or maintenance
- Exclusion of units from analysis due to missing data about e.g. exposure

Selection Bias: Specific focus

A bias resulting from: methods used to select subjects or data; factors that influence initial study participation; or differences between the study sample and the population of interest

- Cell density
- “Representative” sample

Analysis Bias ($n \approx 32$)

Analysis Bias: Definition

A bias related to the analytic process applied to the data

Analysis Bias: Examples

A bias related to the analytic process applied to the data

- Masking analysis
- Correcting for or imputing missing data
- Data reduction, normalisation, standardisation, noise reduction
- Prior knowledge of data before developing analysis plan

Analysis Bias: Specific checks

A bias related to the analytic process applied to the data

- Absence from analysis of predictors of missing data
- Correcting for e.g. selection factors
- Controlling for baseline differences, confounders
- Controlling for time-varying factors
- Use of control data
- Use of software

Attrition Bias (n≈16)

Attrition Bias: Definition

A bias due to absence of expected **participation** or **data collection after selection** for study inclusion

Attrition Bias: Examples

A bias due to absence of expected **participation** or **data collection after selection** for study inclusion

- Exclusion of samples from analysis
- Incomplete data on exposure, outcome, confounders, missing data
- Loss of samples

Attrition Bias: Specific checks

A bias due to absence of expected **participation** or **data collection after selection** for study inclusion

- Cytotoxicity of test compound

Choice-of-Question Bias (n=0)

Choice-of-Question Bias

A bias in research design in which the research question (that the study is designed to answer) is inappropriate for the context.

- No examples, open discussion

Conflicted Interests Bias (n=2)

Conflicted Interests Bias: Definition

A bias in which decision makers influencing research design, conduct, analysis or reporting have goals or motivations that conflict with scientific research objectives

Conflicted Interests Bias: Examples

A bias in which decision makers influencing research design, conduct, analysis or reporting have goals or motivations that conflict with scientific research objectives

- Sources of funding
- Conflicts of interest

Conflicted Interests Bias: Specific checks

A bias in which decision makers influencing research design, conduct, analysis or reporting have goals or motivations that conflict with scientific research objectives

- **Mismanagement** of interests in decision-making

Predictive Model Research Bias (n=0)

Predictive Model Research Bias

A bias specific to the design, conduct, analysis or reporting of research about predictive modelling

- No examples, open discussion

Confounding Covariate Bias (n≈24)

Confounding Covariate Bias

A situation in which the effect or association between an exposure or outcome is distorted by another variable

For confounding covariate bias to occur the distorting variable must be

1. associated with the exposure and the outcome,
2. not in the causal pathway between exposure and outcome, and
3. unequally distributed between the groups being compared.

Confounding Covariate Bias: Examples

A situation in which the effect or association between an exposure or outcome is distorted by another variable

- Method (if any) for random allocation to exposure groups
- Baseline differences between exposure groups

Confounding Covariate Bias: Specific checks

A situation in which the effect or association between an exposure or outcome is distorted by another variable

- Time-varying confounding (confounders that change value over time)
- Other ways in which there can be baseline differences between groups?

Early Study Termination Bias (n=0)

Early Study Termination Bias: Definition

A bias due to the decision to end the study earlier than planned

- Ending a study early or late to generate more favourable results
- No examples

Performance Bias ($n \approx 44$)

Performance Bias: Definition

A bias resulting from differences between the received exposure and the intended exposure

Performance Bias: Examples

A bias resulting from differences between the received exposure and the intended exposure

- Culture conditions e.g. medium, maintenance
- Investigator knowledge of exposure group (exposure, maintenance)
- Error in test substance
- Impurities in test substance
- Solubility of test substance

Performance Bias: Specific checks

A bias resulting from differences between the received exposure and the intended exposure

- Breaking masking
  - failure of allocation concealment due to recognising patterns in numbering
  - visual differences between groups
- Different methods of exposure administration between groups
- Influence of vehicle
- Use of robotic test systems

“Other”

Other: Definition

A distortion in results due to factors other than those described above.

Other: Examples

A distortion in results due to factors other than those described above.

- Choice of source of activation system, given test article
- Inherent physicochemical properties of test substance

INVITES-IN Focus Group

Group 2, Meeting 2 :: 26 June 2023

Mic checks for everyone

What we are doing and how

We are developing INVITES-IN, a tool for assessing the internal validity (potential for systematic error in results or findings) of a study. The tool will be used in systematic reviews.

We are collecting potential bias criteria from existing study assessment tools (literature review) and expert opinion (focus groups)

- Goal of focus groups is to discover more bias items than we captured from the literature (n≈250, 72 assessment tools)
- Objective of focus groups is to gather more data, not secure agreement (agreement process is separate)
- No ideas are wrong: brainstorming sessions where we will try to move along discussion on 12 bias domains

Bias domains (alphabetical)

1. ~~Analysis Bias~~
2. Attrition Bias
3. Choice-of-Question Bias
4. Conflicted Interests Bias
5. Confounding Covariate Bias
6. ~~Detection Bias~~
7. Early Study Termination Bias
8. Performance Bias
9. Predictive Model Research Bias
10. ~~Reporting Bias~~
11. ~~Selection Bias~~
12. “Other”

Structure of the discussion

1. Show you the domain being discussed and the number of criteria we have discovered
2. Show you the definition of the domain, ask what it means to you for the in vitro context
3. Provide some examples of criteria we have discovered, to help discussion
4. Provide some examples of criteria that we have found challenging to interpret, for you to comment on specifically

Notes on structure

- We have chosen which domains and terms are discussed based on previous focus group discussions. Aiming for comprehensiveness.
- Domain choices are to help structure discussion. We are not discussing whether the domains are an appropriate or complete classification scheme for biases.
- I will say some things that may sound naive or obvious. Do not be thrown by this, I will be trying to surface concepts without leading your responses.

Administrative information

- This session is being recorded and machine-transcribed
- Data will be anonymised. Participants should not at a later date attribute comments to specific individuals.
- Facilitators are Paul Whaley (lead) and Gunn Vist (steer)
- Note taker is Heather Ames
- Gro Mathison is the project supervisor and lead, and point of contact for any concerns about today’s facilitation process
- If you have any questions or concerns about process, please raise them now (**any questions?**)

Recording check (on, transcribing)

Attrition Bias ($n \approx 16$)

Attrition Bias: Definition

A bias due to absence of expected **participation** or **data collection after selection** for study inclusion

Attrition Bias: Examples

A bias due to absence of expected **participation** or **data collection after selection** for study inclusion

- Exclusion of samples from analysis
- Incomplete data on exposure, outcome, confounders, missing data
- Loss of samples

Attrition Bias: Specific checks

A bias due to absence of expected **participation** or **data collection after selection** for study inclusion

- Cytotoxicity of test compound

Choice-of-Question Bias (n=0)

Choice-of-Question Bias

A bias in research design in which the research question (that the study is designed to answer) is inappropriate for the context.

- No examples, open discussion

Conflicted Interests Bias (n=2)

Conflicted Interests Bias: Definition

A bias in which decision makers influencing research design, conduct, analysis or reporting have goals or motivations that conflict with scientific research objectives

Conflicted Interests Bias: Examples

A bias in which decision makers influencing research design, conduct, analysis or reporting have goals or motivations that conflict with scientific research objectives

- Sources of funding
- Conflicts of interest

Conflicted Interests Bias: Specific checks

A bias in which decision makers influencing research design, conduct, analysis or reporting have goals or motivations that conflict with scientific research objectives

- **Mismanagement** of interests in decision-making

Predictive Model Research Bias (n=0)

Predictive Model Research Bias

A bias specific to the design, conduct, analysis or reporting of research about predictive modelling

- No examples, open discussion

Confounding Covariate Bias (n≈24)

Confounding Covariate Bias

A situation in which the effect or association between an exposure or outcome is distorted by another variable

For confounding covariate bias to occur the distorting variable must be

1. associated with the exposure and the outcome,
2. not in the causal pathway between exposure and outcome, and
3. unequally distributed between the groups being compared.

Confounding Covariate Bias: Examples

A situation in which the effect or association between an exposure or outcome is distorted by another variable

- Method (if any) for random allocation to exposure groups
- Baseline differences between exposure groups

Confounding Covariate Bias: Specific checks

A situation in which the effect or association between an exposure or outcome is distorted by another variable

- Time-varying confounding (confounders that change value over time)
- Other ways in which there can be baseline differences between groups?

Early Study Termination Bias (n=0)

Early Study Termination Bias: Definition

A bias due to the decision to end the study earlier than planned

- Ending a study early or late to generate more favourable results
- No examples

Performance Bias (n≈44)

Performance Bias: Definition

A bias resulting from differences between the received exposure and the intended exposure

Performance Bias: Examples

A bias resulting from differences between the received exposure and the intended exposure

- Culture conditions e.g. medium, maintenance
- Investigator knowledge of exposure group (exposure, maintenance)
- Error in test substance
- Impurities in test substance
- Solubility of test substance

Performance Bias: Specific checks

A bias resulting from differences between the received exposure and the intended exposure

- Breaking masking
  - failure of allocation concealment due to recognising patterns in numbering
  - visual differences between groups
- Different methods of exposure administration between groups
- Influence of vehicle
- Use of robotic test systems

“Other”

Other: Definition

A distortion in results due to factors other than those described above.

Other: Examples

A distortion in results due to factors other than those described above.

- Choice of source of activation system, given test article
- Inherent physicochemical properties of test substance

INVITES-IN Focus Group

Group 3, Meeting 1 :: 27 June 2023

Mic check & brief intro for everyone

What we are doing and how

We are developing INVITES-IN, a tool for assessing the internal validity (potential for systematic error in results or findings) of a study. The tool will be used in systematic reviews.

We are collecting potential bias criteria from existing study assessment tools (literature review) and expert opinion (focus groups)

- Goal of focus groups is to discover more bias items than we captured from the literature (n≈250, 72 assessment tools)
- Objective of focus groups is to gather more data, not secure agreement (agreement process is separate)
- No ideas are wrong: brainstorming sessions where we will try to move along discussion on 12 bias domains

Bias domains (alphabetical)

1. Analysis Bias
2. Attrition Bias
3. Choice-of-Question Bias
4. Conflicted Interests Bias
5. Confounding Covariate Bias
6. Detection Bias
7. Early Study Termination Bias
8. Performance Bias
9. Predictive Model Research Bias
10. Reporting Bias
11. Selection Bias
12. “Other”

Structure of the discussion

1. Show you the domain being discussed and the number of criteria we have discovered
2. Show you the definition of the domain, ask what it means to you for the in vitro context
3. Provide some examples of criteria we have discovered, to help discussion
4. Provide some examples of criteria that we have found challenging to interpret, for you to comment on specifically

Notes on structure

- We have chosen which domains and terms are discussed based on previous focus group discussions. Aiming for comprehensiveness.
- Domain choices are to help structure discussion. We are not discussing whether the domains are an appropriate or complete classification scheme for biases.
- I will say some things that may sound naive or obvious. Do not be thrown by this, I will be trying to surface concepts without leading your responses.

Administrative information

- This session is being recorded and machine-transcribed
- Data will be anonymised. Participants should not at a later date attribute comments to specific individuals.
- Facilitators are Paul Whaley (lead) and Gunn Vist (steer)
- Note taker is Heather Ames
- Gro Mathison is the project supervisor and lead, and point of contact for any concerns about today’s facilitation process
- If you have any questions or concerns about process, please raise them now (**any questions?**)

Recording check (on, transcribing)

Performance Bias (n≈44)

Performance Bias: Definition

A bias resulting from differences between the received exposure and the intended exposure

Performance Bias: Examples

A bias resulting from differences between the received exposure and the intended exposure

- Culture conditions e.g. medium, maintenance
- Investigator knowledge of exposure group (exposure, maintenance)
- Error in test substance
- Impurities in test substance
- Solubility of test substance

Performance Bias: Specific checks

A bias resulting from differences between the received exposure and the intended exposure

- Breaking masking
  - failure of allocation concealment due to recognising patterns in numbering
  - visual differences between groups
- Different methods of exposure administration between groups
- Influence of vehicle
- Use of robotic test systems

Attrition Bias (n≈16)

Attrition Bias: Definition

A bias due to absence of expected **participation** or **data collection after selection** for study inclusion

Attrition Bias: Examples

A bias due to absence of expected **participation** or **data collection after selection** for study inclusion

- Exclusion of samples from analysis
- Incomplete data on exposure, outcome, confounders, missing data
- Loss of samples

Attrition Bias: Specific checks

A bias due to absence of expected **participation** or **data collection after selection** for study inclusion

- Cytotoxicity of test compound

Detection Bias (n≈80)

Detection Bias: Definition

A bias due to distortions in any process involved in the determination of the recorded values for a variable

Detection Bias: Examples

A bias due to distortions in any process involved in the determination of the recorded values for a variable

- Detection of exposure
- Detection of outcome
- Use of appropriate comparison groups (e.g. controls, doses)
- Masking of investigators (outcome assessors)

Detection Bias: Specific checks

A bias due to distortions in any process involved in the determination of the recorded values for a variable

- Detection of confounders
- Timing of exposure: time-point, window, duration, latency periods
- Timing of outcome measurement
- Use of image analysis and data visualisation
- Equipment issues additional to levels of detection
- Marker cut-off points

Predictive Model Research Bias (n=0)

Predictive Model Research Bias

A bias specific to the design, conduct, analysis or reporting of research about predictive modelling

- No examples, open discussion

Reporting Bias ($n \approx 15$)

Reporting Bias: Definition

A bias due to distortions in the selection of or representation of information in study results or research findings

Reporting Bias: Examples

A bias due to distortions in the selection of or representation of information in study results or research findings

- Choice of reported exposures
- Choice of reported outcomes
- Choice of reported analyses
- Choice of desired result from multiple analyses, subgroups

Reporting Bias: Specific checks

A bias due to distortions in the selection of or representation of information in study results or research findings

- Selective emphasis on post-hoc analysis

Early Study Termination Bias (n=0)

Early Study Termination Bias: Definition

A bias due to the decision to end the study earlier than planned

- Ending a study early or late to generate more favourable results
- No examples

Selection Bias (n≈20)

Selection Bias: Definition

A bias resulting from: methods used to select subjects or data; factors that influence initial study participation; or differences between the study sample and the population of interest

Selection Bias: Examples

A bias resulting from: methods used to select subjects or data; factors that influence initial study participation; or differences between the study sample and the population of interest

- Cell line authentication
- Cell line contamination
- Conditions of cultivation or maintenance
- Exclusion of units from analysis due to missing data about e.g. exposure

Selection Bias: Specific focus

A bias resulting from: methods used to select subjects or data; factors that influence initial study participation; or differences between the study sample and the population of interest

- Cell density
- “Representative” sample

Choice-of-Question Bias (n=0)

Choice-of-Question Bias

A bias in research design in which the research question (that the study is designed to answer) is inappropriate for the context.

- No examples, open discussion

Analysis Bias (n≈32)

Analysis Bias: Definition

A bias related to the analytic process applied to the data

Analysis Bias: Examples

A bias related to the analytic process applied to the data

- Masking analysis
- Correcting for or imputing missing data
- Data reduction, normalisation, standardisation, noise reduction
- Prior knowledge of data before developing analysis plan

Analysis Bias: Specific checks

A bias related to the analytic process applied to the data

- Absence from analysis of predictors of missing data
- Correcting for e.g. selection factors
- Controlling for baseline differences, confounders
- Controlling for time-varying factors
- Use of control data
- Use of software

Conflicted Interests Bias (n=2)

Conflicted Interests Bias: Definition

A bias in which decision makers influencing research design, conduct, analysis or reporting have goals or motivations that conflict with scientific research objectives

Conflicted Interests Bias: Examples

A bias in which decision makers influencing research design, conduct, analysis or reporting have goals or motivations that conflict with scientific research objectives

- Sources of funding
- Conflicts of interest

Conflicted Interests Bias: Specific checks

A bias in which decision makers influencing research design, conduct, analysis or reporting have goals or motivations that conflict with scientific research objectives

- **Mismanagement** of interests in decision-making

Confounding Covariate Bias (n≈24)

Confounding Covariate Bias

A situation in which the effect or association between an exposure and outcome is distorted by another variable

For confounding covariate bias to occur the distorting variable must be

1. associated with the exposure and the outcome,
2. not in the causal pathway between exposure and outcome, and
3. unequally distributed between the groups being compared.

Confounding Covariate Bias: Examples

A situation in which the effect or association between an exposure or outcome is distorted by another variable

- Method (if any) for random allocation to exposure groups
- Baseline differences between exposure groups

Confounding Covariate Bias: Specific checks

A situation in which the effect or association between an exposure or outcome is distorted by another variable

- Time-varying confounding (confounders that change value over time)
- Other ways in which there can be baseline differences between groups?

“Other”

Other: Definition

A distortion in results due to factors other than those described above.

Other: Examples

A distortion in results due to factors other than those described above.

- Choice of source of activation system, given test article
- Inherent physicochemical properties of test substance

INVITES-IN Focus Group

Group 3, Meeting 2 :: 28 June 2023

Mic check & brief intro for everyone

What we are doing and how

We are developing INVITES-IN, a tool for assessing the internal validity (potential for systematic error in results or findings) of a study. The tool will be used in systematic reviews.

We are collecting potential bias criteria from existing study assessment tools (literature review) and expert opinion (focus groups)

- Goal of focus groups is to discover more bias items than we captured from the literature (n≈250, 72 assessment tools)
- Objective of focus groups is to gather more data, not secure agreement (agreement process is separate)
- No ideas are wrong: brainstorming sessions where we will try to move along discussion on 12 bias domains

Bias domains (alphabetical)

1. Analysis Bias
2. ~~Attrition Bias~~
3. Choice-of-Question Bias
4. Conflicted Interests Bias
5. Confounding Covariate Bias
6. ~~Detection Bias~~
7. Early Study Termination Bias
8. ~~Performance Bias~~
9. ~~Predictive Model Research Bias~~
10. ~~Reporting Bias~~
11. Selection Bias
12. “Other”

Structure of the discussion

1. Show you the domain being discussed and the number of criteria we have discovered
2. Show you the definition of the domain, ask what it means to you for the in vitro context
3. Provide some examples of criteria we have discovered, to help discussion
4. Provide some examples of criteria that we have found challenging to interpret, for you to comment on specifically

Notes on structure

- We have chosen which domains and terms are discussed based on previous focus group discussions. Aiming for comprehensiveness.
- Domain choices are to help structure discussion. We are not discussing whether the domains are an appropriate or complete classification scheme for biases.
- I will say some things that may sound naive or obvious. Do not be thrown by this, I will be trying to surface concepts without leading your responses.

Administrative information

- This session is being recorded and machine-transcribed
- Data will be anonymised. Participants should not at a later date attribute comments to specific individuals.
- Facilitators are Paul Whaley (lead) and Gunn Vist (steer)
- Note taker is Heather Ames
- Gro Mathison is the project supervisor and lead, and point of contact for any concerns about today’s facilitation process
- If you have any questions or concerns about process, please raise them now (**any questions?**)

Recording check (on, transcribing)

Selection Bias ($n \approx 20$)

Selection Bias: Definition

A bias resulting from: methods used to select subjects or data; factors that influence initial study participation; or differences between the study sample and the population of interest

Selection Bias: Examples

A bias resulting from: methods used to select subjects or data; factors that influence initial study participation; or differences between the study sample and the population of interest

- Cell line authentication
- Cell line contamination
- Conditions of cultivation or maintenance
- Exclusion of units from analysis due to missing data about e.g. exposure

Selection Bias: Specific focus

A bias resulting from: methods used to select subjects or data; factors that influence initial study participation; or differences between the study sample and the population of interest

- Cell density
- “Representative” sample

Early Study Termination Bias (n=0)

Early Study Termination Bias: Definition

A bias due to the decision to end the study earlier than planned

- Ending a study early or late to generate more favourable results
- No examples

Choice-of-Question Bias (n=0)

Choice-of-Question Bias

A bias in research design in which the research question (that the study is designed to answer) is inappropriate for the context.

- No examples, open discussion

Analysis Bias (n≈32)

Analysis Bias: Definition

A bias related to the analytic process applied to the data

Analysis Bias: Examples

A bias related to the analytic process applied to the data

- Masking analysis
- Correcting for or imputing missing data
- Data reduction, normalisation, standardisation, noise reduction
- Prior knowledge of data before developing analysis plan

Analysis Bias: Specific checks

A bias related to the analytic process applied to the data

- Absence from analysis of predictors of missing data
- Correcting for e.g. selection factors
- Controlling for baseline differences, confounders
- Controlling for time-varying factors
- Use of control data
- Use of software

Confounding Covariate Bias (n≈24)

Confounding Covariate Bias

A situation in which the effect or association between an exposure and outcome is distorted by another variable

For confounding covariate bias to occur the distorting variable must be

1. associated with the exposure and the outcome,
2. not in the causal pathway between exposure and outcome, and
3. unequally distributed between the groups being compared.

Confounding Covariate Bias: Examples

A situation in which the effect or association between an exposure or outcome is distorted by another variable

- Method (if any) for random allocation to exposure groups
- Baseline differences between exposure groups

Confounding Covariate Bias: Specific checks

A situation in which the effect or association between an exposure or outcome is distorted by another variable

- Time-varying confounding (confounders that change value over time)
- Other ways in which there can be baseline differences between groups?

Conflicted Interests Bias (n=2)

Conflicted Interests Bias: Definition

A bias in which decision makers influencing research design, conduct, analysis or reporting have goals or motivations that conflict with scientific research objectives

Conflicted Interests Bias: Examples

A bias in which decision makers influencing research design, conduct, analysis or reporting have goals or motivations that conflict with scientific research objectives

- Sources of funding
- Conflicts of interest

Conflicted Interests Bias: Specific checks

A bias in which decision makers influencing research design, conduct, analysis or reporting have goals or motivations that conflict with scientific research objectives

- **Mismanagement** of interests in decision-making

“Other”

Other: Definition

A distortion in results due to factors other than those described above.

Other: Examples

A distortion in results due to factors other than those described above.

- Choice of source of activation system, given test article
- Inherent physicochemical properties of test substance
