# Supplementary material for: A comprehensive item bank of internal validity issues of relevance to in vitro toxicology studies
Source: Evid Based Toxicol. Author manuscript; Available in PMC 2025 Oct 31. (PMC12180937; doi:10.1080/2833373X.2024.2418045)
Supplement: Supplements [file NIHMS2054894-supplement-Supplements.zip › Supplemental Material 4_Item Bank_Focus group transcripts_R1.docx]

# **A comprehensive item bank of internal validity issues of relevance to in vitro toxicology studies**

# Supplementary Materials 4

## The focus group discussion transcripts

### Focus group 1, meeting 1

Paul Whaley
So what I'm going to do is we're now going to start with analysis bias.
So we found about 32 items. So we're going through normalising sometimes a little bit unclear when one item is a duplicate of another, but we reckon we found 32 unique criteria in the 72 tools that we abstracted criteria from.
So for our purposes here, analysis bias is a bias related to the analytic process applied to the data.
So the data is the information that is generated by study.
The analytic process is the operations you apply to analyse that data.
And they bias would be when those analytic processes distort the results of the study.

Participant 2
Yeah.

Paul Whaley
So first question is, does that make a little bit of sense to people? My second question is.
When you're doing in vitro studies, um, if you're reading them, performing them.
In what ways do you become concerned that the analytic processes so the ways that the data is analysed?
Could introduce error or bias into the findings of that study.
Give me a second to think about it and then I will start putting people on the spot.

Participant 4
Okay perfect.

Paul Whaley
So Participant 4, please just cheque and don't worry about putting hands up and things if it becomes too much of A free for all, we'll go back to hand raising. But to start off with just just talk.

Participant 4
OK, first thing is if they don't disclose how they actually did it, if they didn't disclose any details, so I'm not sure if we should point out how it should be done correctly or.

Paul Whaley
It doesn't matter. It's up to you if you feel like it's important to say. Then say it.

Participant 4
And in which details should we go like at the analysis. So for instance, if they the data to the control or if they re normalizer. So I would start with this one.

Paul Whaley
Very good.
So normalisation did come up and our criteria a lot. So it's interesting that you raise that. So ohh Participant 6.

Participant 6
Yeah, and uh, just to follow up on that, uh, a lot of times when I review papers, I miss Miss Data, so data are not not accidentally or they are accidentally left out. So the crew did are missing or yeah, the steps in between are missing. So either the final result is only giving but you cannot trace back how was processed. So any intermediate steps are often missing in the data analysis. So that makes it hard to reproduce. I'm not sure if it leads to bias. That's another thing, but it's definitely hard to reproduce what and to cheque what someone is doing and if there is a stop missing.

Participant 2
Yeah.

Paul Whaley
So you say it makes it hard to reproduce, but if you see what do you often like? If you have the data available and the analysis process has been described, what kind of things would make you worried about potential for bias that error? If you could see it right?
That makes sense to us.

Participant 6
But yeah.

I have to think about it and the problem is that well, I want to control the whole unfollow and there's no worry that it leads to bias. But you cannot cheque if mistakes have been made during the analysis. So if you.
Yeah, misinformation in of intermediate steps, and only the final results is given. You just cannot cheque. And if if people make mistakes so the buyers might be, yeah. Concerned for mistakes which often happen unit conversions that are not correct. That's the most of the times. Millimolar conversions and if you don't, cannot follow these intermediate step, you cannot see the. Yeah that it happens.

Paul Whaley
Umm OK.

Participant 1 you put your hand up.

Participant 1
Yes, um, for example, when there is there dose response curves and they fitted curves are involved. I think it would be good to know which model for example was chosen and for what reason.
Because sometimes these details are not given, so we are not sure whether this field curve model that was chosen was the right one or not. So I think it's good, be good, it could be good to be justification below.

Paul Whaley
So it's an issue of the choice of model not being made clear.
So then, presumably the role model would result in a error and result.

Participant 1
Yeah.

Participant 2
Hmm.

Paul Whaley
Yeah, I asked really stupid questions, some facilitating. So. So what sort of. So when it comes to people like, you know, have an analysis model.
What sort of red flags maybe would be raised when you when you look, if someone does report the model that they used, what kind of errors do you think people could be making a model selection?

Participant 1

For example, R square for example should be reported.
Or slow or several care parameters.

Participant 4
If I may relate it to that, So what we do, we have a pipeline that creates multiple models fitting different types of curves and the best fitting model is then automatically picked. So this would also allow to, I don't know, prevent that they won't take the best model to best suit their their results. I mean the result they wanted to see and not the model they should use..

Participant 4
But of course we can't do I think multiple models in every study.

But it could be a solution and I thought about another one if they checked for how the data are distributed. So the normality of data. Yet it's amazing thing, but that should be definitely there.

Paul Whaley
I guess.

Participant 2
With regard to the to the distribution of data, it might also be important to take into account that if definition of borderline ranges.
At least this is an issue that is often discussed when it comes to their development of test guidelines. When you have done in certain yeah, certain model to derive it, a decision that the question arises how, yeah, how to deal with bot liner if there are borderline ranges and how to deal with the results in this area.

Paul Whaley
Very good. Excellent. So.
Okay, you might want to put aside around that one, because I think we'll be coming back to that in several places.

Participant 6

Participant 6
Never said respected this, um, statistical distribution. I'm not sure if it relates to that, but you see sometimes use of log scale and non lock skills log scales and also I see a misuse of it sometimes that people want to show nonlinearity and put it on log scale. That that's that's just due to the the log scale and not because there is actually no non linearity so.

But that might not relate to the distribution. I'm not sure, but how to it relates to the the dose response curve, it's non log scale or not?

And, but you need a statistician and statistician, maybe to to correctly.
Formally that what what will be good? I mean, that's not the goal of now. Today of course, what will be good or not good, but at least you see see errors and BIOS body use of statistics. Them and the use of log scale.

Paul Whaley
I'm good. Thank you. Yes, so. In terms of some examples that we did see.
One thing we haven't really talked about much yet, but I at least you also wanted to revert to you about the normalisation issues because in some of the tools that we're abstracting from, there was talk about masking the data to the people analysing it so that they wouldn't necessarily know if the data was associated with the which exposure group, say the data was related was related to you, so they can make it harder, I suppose, for people's expectations about the results, they should get to influence how they perform analysis.

We haven't talked much about correction for imputation of missing data, so it would be interesting to your thoughts on that. Then there are data reduction, normalisation, standardisation, noise reduction issues that I was hoping maybe we could get into a little.
And then there's also been some issues or concerns raised about prior knowledge of data before developing an analysis plan. So again, we know what the data is, develop the analysis plan in response to the data, and then you obviously is kind of like an overfit problem, right?
Does anyone have any thoughts about maybe the first instance masking the analysis?
And that's quite similar to the prior knowledge of data, so. Any ideas or thoughts or experience in that space?

Participant 5
I think this is very important aspect actually to have this mask on my system, blind evaluation or whatever you call it, and unless you have a I mean, if you have some. And just played reading or whatever. Then it doesn't matter because it's automatic.
.
Yeah, measurements or or the values, but if there is manual work involved, of course. It's important that it's blind to to avoid that your Ohh so to influence by knowing what to expect maybe or what you want. To hope to to get from your analysis.

Paul Whaley
Okay, thank you. So that's seventy thoughts or comments.

Participant 1
Can't.

Paul Whaley
Yep.

Participant 1
Sorry about the last point. Prior knowledge of data before developing analysis plan I think is what Participant 4 said before about that. We should know them.
And for example, what that we should follow parametric and non parametric test so the normality of data Is really important.

Paul Whaley
Very good.

Participant 1
If it's what is meant by the this 4th bullet.

Paul Whaley
It in a way, it doesn't matter what the 4th will, it means it's just the these points are all good points so.

Participant 5
I think I think it's also important to to have some acceptance criteria for the the assay that you using.

Participant 5
Based on historical control data in the lab for the method and for the cell line applied so that you you know what to expect and then if the controls are working as they should, so that sort of thing.

Experiment. Can be accepted ohh or not not just to include controls but also to control the values of the controls included in experiment.

Paul Whaley
For the record, this is actually one of you touched on one of the things that we were not sure what it meant when we were abstracted from the tool. So by acceptance criteria, can you just expand on that concept a little?

Participant 5
Yeah, I I think I mean, for instance, when we are doing cytotoxicity testing. Hmm, we have this acceptance criteria that the positive control should be like within at least I think it's. And at least 80%. So I built you, let's say 20% reduction on and so that's the sort of minimum data policy control should give, but preferably further down and same with them negative controls. And I mean for.
Yeah, we are doing a lot of genotoxicity testing DNA strand backs for instance. And there will always be some background and strand breaks in the cells. So it's important and to know and and this depends on the cell line applied, some cell lines can have low background levels and others can have high. So it's important to know this and they know the cell and that you're working with even though you are working with the same method as long as we do it across cell lines that can be huge differences when it comes to negative control then so.

Paul Whaley
Ohh.

Participant 5
MMM. And then and if you then go about this and you have to sort of and and that can be defined in the lab, then you have to set some criteria. So if it if the data to get for the negative or positive control are deviating too much from what you expect based on the historical control data, then it's not sort of valid experiment then because it will not fulfil the acceptance criteria. That you have defined.

Paul Whaley
Very good. Thank you.

Participant 2
Um, there's another thing that came into my mind when regarding the last bullet point, the prior knowledge.

Participant 2

Um might also go into the direction that it is important to have an independent. Training and test set with with regarding to your method. So that's. Um the the model or yeah, the prediction model. Um has been derived within really independent. Um training set and that that the compounds of the training set are not part of the test set. Then later on to be applied. Since this would indicate a better performance than, yeah.

It then there is an in practise I I don't. I don't know if it was clear enough, I hope.

Paul Whaley
So this is a is this specific so which. So I guess that's a very interesting point you make. So which sort of research, Council study design context that we're talking about here for having like a training set with compounds in it? There's this for.

Paul Whaley
What? What type of study design?

Participant 2
Yeah, for for, for in vitro studies before setting up a prediction model, you would, you would have to select an appropriate set of reference chemicals. Covering. All all ranges of responses you might expect.
And then you would set up your prediction model based on the training set and you you would verify it in test set with additional chemicals and you would yeah, to really properly define the productivity of your model. You would claim this is a basic of basis also of the OECD validation process. You would claim that training set and then test set are really independent. So that there's no no buyers resulting in in in a better prediction.

Paul Whaley
Good. OK.

Participant 3
I I wonder, I think I have a bit of difficulty understanding because that really relates to the what the, what, what what the data are going to be used for. So I think I always have difficulty trying to separate this internal validity and external validity so so. So I think because we both work in the area of application, I think the same thing but on the other hand you could be doing the in vitro studies for a different purpose. So it wouldn't necessarily be a prediction model that so.

And again, it's a bit difficult to kind of separate them.

OK.

0:33:4.650 --> 0:33:5.200
Participant 2
OK.

Paul Whaley
That's totally fine. We actually have. There is a prediction underneath all of this. There is a prediction model element that I didn't introduce on that first slide, but I think we're going now going to build into a future one of the follow up focus groups because.
It wasn't something we're expecting to come up, but now has done so.
Yes, that was actually very helpful. So before we move on to the next slide, I'm just going to put Participant 4 on the spots to talk a little bit about data reduction and normalisation and noise reduction and things. Just so we get a few more concepts around that.

Participant 4
Yeah. So yeah, actually to the, to the noise reduction. So we are so in our test methods. So we we are working with the DNT in vitro battery and we consider a noise, the lowest concentration of any chemical tested that is not supposed to cause any effect. And we consider the slowest concentration as a noise that we measure across all the essays just to have this noise in there. And but I don't really see what you mean by noise reduction. Like if people try to manipulate the data and change it.

Paul Whaley
We're not really sure why what they meant either, but it did seem that way from the tools. There's like there's some noise in there, so we analyse it in such a way that we squash the noise and that makes the signal more apparent. But.

Participant 4

So no, no, we are just like checking if it's in the normal normal range. And of course if it's not then either the concentrations are wrongly picked and we should test it again or we just yeah, it's wrong test. We cannot use the data.

And this applies also, as was mentioned to positive and negative controls. So for every historical data we have, we have coefficients of variation for both positive and negative chemicals. But.

Yeah, and the normalisation well, again, we tried multiple multiple possibilities and what fits best DSA and the purpose should be used. We shouldn't pick anything that will just magically make the data the best looking possible.

Paul Whaley
Hmm. So ohh Participant 6

Participant 6
Yeah, no, maybe I I like you approach of the the best fit by by computer and because that's less biassed. But what I've also seen and that's that's maybe good to mention or not, but that people tend to rely on the computer simulation completely and do not look at the results. So you can have a complete just because your data are or that there is a mismatch that none of your data points are actually fitted, but they still accept the results.
Just because the computer just said that it fitted so there's a bias. It's good that there are now so many computer models that can simulate nice dose response curves, but there can be also bias if people don't look at it and the results. And then it can lead to very strange, yeah, interpretation in the end as well.

Paul Whaley
We're good.

Participant 4

Now agreed.

Paul Whaley
The specific cheques that we wanted to do, we just covered software which is nice because I think what software must mean it's going to do with the software and it's. Models and system wasn't that provides. I think we've covered because we were talking about that.
Um, there's something that came up which was originally developed kind of like in an observational study context, but looked like it might have application in vitro context. As it appeared the time posting exposure and then maybe taking measurements at different times place the exposure and then you will have time varying factors that need adjusting for in the analysis.

Could maybe one or two of you. Kiss my thoughts as to whether or that is a meaningful idea in this space in some way. Right. It's not meaningful.

Participant 5
Well, I think this controlling for baseline differences something was quite in the same line as, but I was talking about it is.

Participant 2
No, no.

Participant 5
And historical control data and and things. And and I don't know what you mean by this selecting factors and, but of course it's important to. Select a cell line that you know. That you're able to to to detect the endpoint that you you're measuring. And for instance if. If the chemical needs metabolic activation, for instance, for to give toxicity, you have to ensure that the the cell and you're using is metabolic has the metabolic capacity then to to metabolise it, otherwise you need to add external factors.

Paul Whaley
Okay.

Participant 5

So I think it it's not like all sidelines is suitable for any endpoint..

0:38:37.830 --> 0:38:51.720
Paul Whaley
Yeah, I mean, I would just seem that's the case. And yeah, we were just tightening criteria from tools and some of them were like that looks a bit strange. So these are just some cheques on some things that we want to make sure that we aren't missing anything. So that's good.

Participant 4
And if I might maybe to the time varying factors. So again, I'm not not really sure what what they mean, but I think it's important to know if you want to measure the the effect of a chemical or if you want to include as well possible feedback loops or that that the cells are reacting to it and adapting and so measuring in a in a later later time after exposure. So this should be also taken into consideration.

Participant 2
Hmm.

Participant 5
Here. Yeah, you shouldn't ensure that exposure time that you're using or the post exposure time before measuring and it's it's correct. And let's say for very few if we go back then to DNA strand breaks, this is an reversible effect. So you need to to measure at early and late time point and to see if it's being repaired or if it's. Then not been manifested yet also so. Yeah, so this time varying factors are very important, I think.

Participant 3

Get that? Sick.

Participant 5

Hmm.

Participant 2
Yeah, but it additional this time varying factors are again also related to the historical control data, right, that you have really a database available over time to make sure how reproducible your essay is.

Participant 5

Yeah.

Paul Whaley
OK, Participant 3, you were just saying something.

Participant 3
Yeah. Yeah, just at that time course data really critical in terms of if you're using in vitro data in a mechanistic construct like if you want to apply it in that. So that time course is really critical. So you really want to be sure that they've measured at the right time point. No.

Paul Whaley
Yep. Good. Excellent. Right. So we've done 22 minutes on this first domain. So we should move on.

So on next to domain is attrition bias. So we had 16 approximately. Brighton have been put forward across the 72 tools for this type of bias. Define attrition bias as bias due to absence of expected participation. Or data collection after selection for study inclusion. So the place these definitions are taken from is tries to be kind of topic agnostic, so the participant in this case would be the either cells in. Ohh the sample or like a maybe a plate or something. Just whatever your experimental unit is. Um, thanks. Then you've got so participation can. Would cover loss of participants, right? So the analogy from a animal study would be kind of rats dying and then dropping out study from humans. It would be humans in a clinical trial either not participating because they didn't get on with the treatment or because they passed away. Or attrition can refer to when you're expecting to collect data and it doesn't happen, right? So you've got data that's just kind of disappearing out of the system and we're not quite sure why, right. And this can introduce bias into a study, because that can be systematic differences between the things you collect data on and the things that you don't. So how might attrition bias apply in the in vitro context? So Participant 6

Participant 6

Nothing. Most of the attrition bias is taken into account already. For example, cited took this cytotoxicity tests are performed that you don't lose yourselves due to cytotoxicity and that you should not perform them at side to toxicity levels to measure specific effect.

Yeah, that's the yeah. So I think. And it's it's more controllable than that. For example, with participation studies with humans, with cells. Yeah, it's you see, when you lose and you probably know the reason why you lose data points.

Paul Whaley
But if you were.

Participant 6

And it can be taken account.

Paul Whaley
Yeah, but if you were reading a study and you were like, do I trust the results of this study or not? Would there be anything that you'd expect to see in that study that would make you more confident that there weren't any attrition issues? Would there be things you could potentially see that would make you think that attrition might actually be a problem there?

Participant 6

Oh, absolutely. I think people don't report it always and they they just ignore problems and like they they they take the best. . Yeah. That that if they got a loss of cells then it's not always reported normalisation to DNA levels for example you can also do as an instant way of standardisation or loss of mass but that's not always done that's true. And there can be a bias due to that. But I do think in general people don't. Don't. Yeah. Are willing to to correct for anything or any loss. And it's a common practise nowadays, but I I guess there are still publications out there where they.

Do you see? Yeah, inhibition of growth, for example, that that you have cells in one essay are still multiplying in another not or in the control. Not and and there's no correcting for the difference in numbers cells for example, so that it can still happen.

Paul Whaley
Hmm Participant 4.

Participant 4
There I think what would increase the trust in the results would be if they used some specific markers for the specific cell types so that I don't know that they don't know changed into changed, reversed into some, I don't know IPS state or if they developed into some other cell type or if they usually so fax analysis or I don't know standing using some markers to prove that the cells are still what they are supposed to be.

Paul Whaley
Thank you. Thank you.

Paul Whaley
So. Some of the things that we saw.

And the included assessment tools were concerns about samples being excluded from analysis.
So the samples kind of they don't die off or anything, but for some reason they're awkward, so they're not being kind of incorporated in the final analysis. Incomplete data that we've talked about on either. Exposure or outcome or compounders that's an issue. Or you can also have missing data about missing data. In fact, where people haven't reported how much missing data they've got, and that's kind of a weird matter attrition, but surprisingly common. And then also just the loss of physical samples. To this mic, anything spring into anyone's mind about things people can get wrong when they're doing and in vitro study that would introduce that into results.

Participant 4
So I'm in the mean of verification. Instead, if they publish raw data so that we know that nothing disappeared.

Paul Whaley
Hmm. Okay. So is the feeling that I get, I get the feeling that this is fairly complete and fairly. Uncontroversial. Would you say would anyone? Have a comment in that direction. So yeah, the only thing that we want to specifically cheque was this issue of cytotoxicity of the test compound because we weren't really. Sure of the specifics of that in relation to the tools that we were looking at. So Would you one or more of you be able to explain kind of what what goes on with? You know, is this is this a dose level thing is it specific to certain study designs like would you kind of visit a different issue and say genotoxic studies versus some other like endpoint that you're looking at? Um, because we're just trying to think of ways of, I suppose, operationalizing our understanding of what goes wrong with cytotoxicity, how it might not be noticed or taken akin to account properly in the study and then distort the findings of the study.

Participant 6
Ohh, generally it represents an overdose from my in most cases of course and all. Yeah, processes are the 3rd and DNA damage. I'm not sure there was one. I'm working on DNA damage but I I guess it cited toxicity levels. You will see more double strength breaks. So we do you think it's double strength breaks but it's just slightly toxicity. Just general leaking of cells, so you you lose protecting.

Paul Whaley
Ohh.

Participant 6
But cytotoxicity can also be part of the mechanism. So yeah, it's it's not, it's both. But it's a test to make sure that that that you're not overdose in the cells. So that's yeah, generally it happens at high concentrations. Ohh but if it decides the toxicity, I happens already at lower concentrations in the at. I don't know what is low. Yeah, it's 10 to 20 micromolar then then maybe it might also be a part of the effect.

Participant 3
I mean for many.

Participant 1
I think also that the. I think gossip have been several instances. It's good to to try more than once to toxicity methods, because maybe. Once at the toxicity method is not enough, so we need like 2 or or more. And also I wanted to say that, um. And it's good also to to to measure this, it's accessity ideally um, either in parallel with then and point you are actually measuring or even better. And if we are talking about, for example for. Experiments performed in multiple plates. It's the ideal situation is to measure in the same way. . So. Hmm are the same misses and the same experiment the same, well, same cells. To measure both the endpoint of measurement and this toxicity..

Participant 2
Yeah. And there are also quite a number of different endpoints for which OECD test guidelines exists that also specify a range of site toxicity. For example, like skin or irritation.

Participant 4
And they should measure it every time at every well played, and not just once. And then said Ohh it's fine.

Paul Whaley
OK, so that's interesting. So there's a potential then that someone could be measuring cytotoxicity, but it's the maturing in some worlds. We're not all worlds and there will be a difference between the worlds in which the measuring it and the wells in which they're not measuring it and that could distort results. Am I correct in that salary, or at least not completely wrong? Is that you need to speak cause you won't. Nodding won't work on the transcript.

Participant 2
Yeah.

Paul Whaley
Very good.

OK. So we'll move on to the next domain. So this is an interesting one choice of question bias because I think this will impinge a little bit on externality discussions. But there are ways in which choice of question can distort the findings of the immediate study.
So choice of question bias is defined as a bias in research design in which the research question the study itself designed to answer is inappropriate for the context. Didn't come up at all, and any of the the assessment tools that we reviewed. But it is in the choice of question bias and does appear quite regularly in upper literature. So I have no examples to offer you at all, so this will be a completely open discussion of how you think. Choice of question bias might apply, if at all in the in vitro context.

Participant 2
Might be quite important I think, because. Um, we already discussed about the selection of the self system. That that has to be related, of course, to your research question. For example, when when you require some kind of metabolic activation or. Yeah, yeah. That that you would don't should not use liver cells when you want to address some don't know issues related to breast cancer or things like that. So that so that you really have to be careful regarding the selection of the appropriate style system to address your specific question.

Paul Whaley
Participant 4

Participant 4
And even if you are in the in the organ or in the target organ you want, you need to be sure that all the cell types are represented there. That might influence each other and how they behave and also the cells might be coming from different individuals from different sexes. So having both sexes covered, that's super important. The right developmental time point or adult time point depending on what is your question. And yeah, what what strikes me every time when reviewing papers is when they wanted to. Assess and neurotoxic effects on a normal yeah on a normal healthy adult and they choose a cancer cell line for it.

Paul Whaley
Interesting. So it becomes an issue of in the feels like it's an issue of interpretation I guess. Ohh so you you'll be understating or overstating it like I guess in the case of neurotoxicity, if you using cancer cells cell lines then do you think you'd be at risk of overstating your toxicity or understating it?

Participant 4
Now both are possible depending on what you are. Yeah, studying, but definitely if you want to know effects on the healthy, yeah, healthy human then using cancer cell line that will behave probably differently. It's not the best idea.

Paul Whaley
Ohh, let's have any thoughts or comments.

Participant 6
Now I agree that that sometimes the model of choice might not be appropriate. Indeed to the question. So that often happens. I'm not sure if I agree that well, we don't have to agree here, but they also always need the most perfect and and most look like tissue. You can answer questions with healthy or unhealthy and cancer tissues. So there are questions, but the questions should be appropriate to the design of the yeah or in vitro methods should be able to capture what you want to look for metabolism. If you need that, it should contain metabolism. I agree with that.

Um, there was another question of choice of question by is. When I read the title only I was thinking about. That a lot of times I see or not a lot of times bias in hypothesis already, so people are. Um already not open to you and and yes, no answer now. It should only be and yes answer. This is a very problematic chemical. Ohh yeah. And then all the methods are there designed to answer that. It's yeah indeed the gives them correct time. I'm exaggerating a little bit but.
And it's it's a bias in in the question that is being asked in the 1st place. Don't wanting to hear that there might also be no coming out of the the assays or another result. In a way.

Participant 5
I think it's also the the plate design is important also where you put your your samples and your controls because you you can have influence from the positive control on on the other groups, especially if it's volatile compounds. So you you have to ensure that. And the affected your mushroom is not due to contamination from the poster control.

Paul Whaley
I'm good. Next month.

Participant 1
Ohh also there.

Participant 5
Because this this is so on several locations actually that if you put them too close together with no with not too many wells in between, you can actually have a. And the fact. Will you treat themselves? For me.

Paul Whaley
Yes, but I think. Thank you. Yes, I think I heard Participant 2 just interjecting there as well. Participant 2, was that you?

Participant 2
No, no.

Paul Whaley
OK.

Participant 1
Yeah. So, uh, apart from the test systems, which is very, very important, and to assess whether it's. Fit for purpose. Let's say for the question that you want to answer. I think also the exposure is really important and to ohh. Especially if you if we want to relate it to human relevance or to wildlife relevance, the exposure should be physiologically relevant.

Paul Whaley
Umm. Thank God.

Excellent. Right. I will leave his on to the next domain because there are some that are getting interesting and complicated. So the next domain is conflicted. Interest bias came up quite a lot, but there are only two distinct criteria that have a proposed with kind of interesting for something that obviously is of concern to people.

So the conflicted interests bias the definition they were working with is a bias in which decision makers influencing research design, conduct analysis and reporting have goals and motivations that conflict with scientific research objectives. So the idea here. Is that the people who have to are not necessarily advising on a project, but are making a decision about how the research is going to be designed so the study design. Who are involved in the conduct of the study? Who are doing things? He will analysing data or making decisions about how the study's going to be written up. They have goals and motivations that conflicts with the objectives of the study. So they can be pulled in two directions if you like. Typically, these cover sources of funding and conflicts of interest. And I'm just wondering how you think that. Conflicts of interest. Might. Be at play when you're looking at a study and you're trying to understand whether or not the results might have been distorted.

Participant 3
I I think this has interplay of all with how you pose the question.

So I I think that. So again, how you post that question is absolutely critical and sometimes it. Drives you to the answer, and if you're influential in that. You know, designing that research question or. And the protocol. And it can sometimes relate to it. I think of it more as. Bias relative to experience, not so much sources of funding and conflicts of interest, but it's just you're you're accumulating experience. And interest. That can kind of. By is how the questions asked particularly.

Paul Whaley
Does anyone else have any comments?

Participant 6
And the example that it just gave, indeed I think that relates to what he says sometimes also that the people are in the field for a long time, maybe I do you mean that that, that they tend to follow up in their own biases or their own research, right? Yeah.

Now.

Participant 3
Right, right. It's it's not, it's not just necessarily sources of funding or, you know, a conflict of interest is just.

Participant 6
Yep.

Participant 3
Accumulated experience and interest. Yeah, that they may be biassed simply because of. That the rate they maybe they may be entrenched.

Participant 2
Yeah. But.

Paul Whaley
So is it something around having an under obviously like? As you gain experience, you gain understanding and knowledge of the space. You've not necessarily seen any data, but you've seen a lot of other similar data I guess. So then you can build experiments and things in such a way that you know you're going to have a good chance of a positive result. Kind of fits in with your understanding how this stuff works together cause it's a known system and in some way that operates as a distorting effect. Is it that they're finding? Positive results were there shouldn't be any because that would be a distortion. Or is it more of researchers going down a particular track? I'm just wondering.

Participant 3
Yeah, I I think you're not fully testing the hypothesis. I think if that makes sense. Ohh and how you framing that hypothesis derives from your prior experience, so that's pushing it in a certain direction.

Paul Whaley
So is it? It's like A cause. I'm just one of the things we try to understand is that if it's like an additional factor on top of the other biases. So we worry about how the analysis was conducted. We worry about how. Like a. Detection methods and things using the study, we worry about other things. Is it the the interests of the researchers are? Going to be manifest through those. Choices they make as it relates to the steps of planning and doing and reporting a study. Or is it something that's additional?

Participant 3
Yeah, I think it's the interests of the researchers are reviewers. Actually, it's not just the the folks that are funding it, you know, or it's not necessarily that the reviewers have the, you know, declared conflict of interest because at this point, you know, those conflicts, it's it's, it's really an experience bias. That's what I consider it to be.

Paul Whaley
So I'll just wondering, what would you? Do if you are reading a study that would make you concerned about. I suppose distortion in the results. And what would what do you think would be done to mitigate that distortion potentially?

Participant 3
I I think it relates particularly to how the question is posed and looking at how that question is posed and you know, I think we talked about that previously that you can tell by the way the question is posed that you're looking for a certain outcome and that can be influenced by a lot of parties. Single and a lot of factors in my view actually so. And not just sources of funding.

Participant 2
Okay.

Paul Whaley
Yeah, yeah.

Participant 2
But.

Paul Whaley
So we've not got non financial. Conflicts of interest on this, so we can. Probably at that was, it didn't come up in the tools, which surprised me a bit. And then there's an interplay between choice of question and conflicted interests, which we should look into as well and.

Participant 6
Yes, so one um, conflict of interest that I have seen is the pressure that is put upon researchers. And so they need to score high quality journals, high quality papers. And I think that that point is even the pressure is bigger than than sources of funding. I mean, people are always pointing in industrial funded research is is different or it's it's it's always heading towards the best result for industry does not. Well there might be examples. But it's it's not. Yeah, people are discussing these conflicts of interest always in that relation, whereas I think out of, yeah, the, the the pressure that is on researchers that influences their choices. Is not yet taken that much into account because yeah, it's. It's all about, yeah. Um. Scoring high? Yeah. Quoted papers and. And obtaining budget and that can influence the way they results are presented and interpreted the perverted cheap, sorry and analyse that in a way.

Paul Whaley
At least you go.

Participant 4
Yeah, it was a very good point.
I'm I don't agree with it and I have another another thing that I thought about about the conflict of interest. So what would make we make me be worried about the data or the paper is when the authors send it to their own journal like when you are on the scientific board of the journal or I don't know your close friend and then yeah, it's more probable that it will be accepted or that the reviewers will be nicer. That's a problem.

Paul Whaley
I can definitely happen.

Participant 3
That's really common too. Ohh.

Participant 2
May I may I add one thing?

Um, yeah. With regard to pressure, I think there's also. Pressure is on on evaluators of of studies, not only on on researchers carrying out the studies. Um for example. Um, even if you agree, then on on on the the way, how will the question is posed, the interpretation of results can be rather different when it comes to the assessment by an evaluator. For example, on the OECD level, we have the IATA case studies project. They are quite often. Crazy case studies what might not quite often, but sometimes there are case studies submitted by industry whether certain weight of evidence approach for as a final conclusion for the outcome of the classification. Um, office substance and um. Yeah, quite often it happened already that. The regulators. Counter checking these interpretation of results do not agree on this. Some on the the hypothesis made by by the industry.

Paul Whaley
Interesting.

So I just moving on to the specific cheques we wanted to do on conflicted interests. So I guess one of the specific shows what it was, which we now have actually covered and I missed was the non financial. But then one of the things that we also didn't really see discussed or mentioned was.

How is one thing for people to have interests? So it's difficult to do a study when you're not interested in it. And quite a lot of it is about mismanagement of interests, in fact. So if you've got people with. Interests that conflict with the object of the study and you don't manage them. That's why they can have impact. So sometimes interesting managed by keeping people advisory rather decision making roles. Um, I'm just wondering if you have any experience or have witnessed any instances of maybe the way in which interest and managed which might shed some light. On understanding how to evaluate conflicts of interest by advice or understand how interests can distort the findings of the study, in addition to what we were just talking about.

Participant 6
I'm not sure if you're. Yeah. Yeah, understand it correctly, but what I see a lot is that it's very difficult to judge. Ohh, conflicts of interests And did sometimes she did appear as in conflicts, but they are not actually conflicts and. It's very difficult to, yeah. And to really get a a good control over these these. And. Interest, but I'm not sure if that's what you mean. OK.

Paul Whaley
I don't mean anything, this is just an elicitation of ideas though.

Participant 3
Yeah, I I think the issue is not so much mismanagement. I think it is defining conflict of interest in the context of of the study or application and it varies greatly, you know, among different agencies. I think everybody takes a conflict of interest into account. And so it's defining it, but it's also the process for ensuring that you know it's the it's, it's the adequacy of the cheques, it's you know all of that. So it's it's a pretty complex process and itself actually.

Paul Whaley
I know about that.

Participant 3
Yeah.

Paul Whaley
So does anyone else have any final thoughts or comments on conflicted and trespass?

Participant 1
When are we saying decision making doing mean, for example, risk assessment or risk management because.

Paul Whaley
Ohh, it's in the decision making in the conduct. So the design, conduct and reporting of the study itself, not the people who then use the study afterwards.

Participant 1
OK.

Paul Whaley
Have been developing analysis plan, conducting analysis, administering the. Exposures and things like that. And the thing where someone's having to. Do something right and make a decision about you know how they do it, what they do.
Okay. So we move on to the next one, so.

Participant 2
Fire. Fire.

Paul Whaley
This came up a lot because. What we saw 20-4 different examples approximately of how can the founding covariate bias is. Understood. In the tools that we were assessment tools that were abstracting. So compounding covariate bias is a situation in which the effect or association. Between an exposure or outcome is distorted by another variable. So if a compounding covariate bias to occur, the distorting variable will just be one associated with the exposure and the outcome. 2 lost in the causal pathway between the exposure and the outcome, and three unequally distributed between the groups being compared. So. It seems in vitro studies are kind of interesting because sometimes they use human tissue. And they're not necessarily randomised. So there are interesting ways in which you can have confounders potentially at play. In an invitro study. But we wanted to ask you. You know again. Your understanding of compounding covariates and how they may be at play in in vitro study designs. We would like to start. That's a tricky 1. I would worry.

Participant 6
I mean, does it relate to false positives? Um, So what I've seen is that you have chemicals that are colouring or can interfere with an detection methods. So you seeing that the camel glass doing the effect, but it's actually the interference with the detection method. Fluorescence for example often used as detection that that would lead to yeah, it's not an effect by the chemical, but it's just a confounding. But you will not call it confounded. It will be a false positive. Would say maybe.

Paul Whaley
So I guess anything that distorts the study is technically confounded, as that's one of the slightly awkward things about language in this space.

Participant 6
Yeah.

Paul Whaley
But I think it would be. What's happening is that there's something about. The Administration of the exposure. And the. Effect that has so the outcome in the. The the study. Where you've got some sort of distorting other variable at play, so you could have. So maybe what I'll do is I'll just move on to this specific examples. So the typical way that we try to get rid of confounding and research is to have random allocation of the study units. Um to the exposure groups because what that does is it means that the baseline differences between the experimental units, his averaged out, right. So you're the same across space. Ohh so if you've got multiple explanations across each exposure group, right? And that gets rid of the baseline differences issue. So maybe the question is, is with confounders or confounding covariates in in vitro studies? Are there ways in which you can have baseline differences between your study groups in an in vitro? Context.

Participant 4
Typical error would be having not the same concentration of the solvent and all the tested concentrations and negative control, for example. And also, um, somebody already mentioned how it is. Ohh, how the concentrations are plighted in in the well played if if it's randomised or not and sometimes I have the experience that some older machines they measured a little bit more on the right side or on the left side or at the borders, I think it was either absorbance or fluorescent so. Ohh yeah. Paying attention to this as well and of course putting it in the paper. So how how it looked like on the wall plate.

Participant 5
And then, yeah, when they're talking about this plate design, also, of course the edge defects is important to take into consideration.

Paul Whaley
Hmm.

Participant 5
And what what what's mentioned about interference also it's not only false positives but also false negatives that can occur then especially with nanomaterials, which quite often interfere with fluorimetric. As is and can also mask um fluorescence. So like a false negative then? So in deference, controls are very important to include and especially when working with the particles.

Participant 2
But.

Paul Whaley
So is there ways so one of the things that comes up in some of the notes around the tools that we have included? Is that they talk about kind of homogeneous. So I will solve this basically, so the say confounding at least for differences based on differences in the cell populations, right? Don't matter because the cell preparation is not some homogeneous makes the sales so. Don't have to worry about it, but all their ways in which you can have baseline differences between yourself populations, either I guess between Wales or between plates or something. Whereby you'll have a systematic difference between. Say that the cell population you were exposure group versus your control group, or between exposure groups and some way can that happen?

Participant 4
I think if the time of exposure is crucial. So for instance, I was doing some experiment um, where it really changed. If it was 10 minutes later or not. So you need to randomise. If you first expose first concentration or the highest concentration or not doing first the 1st and the 2nd then the 3rd and the 4th and then have 10 minutes. The difference between negative control and positive control on the other side. So this could definitely in use bias.

Participant 2
With regard to anonym materials, it's, it's also quite important that you have a thorough characterization of quite a number of physical chemical parameters. Um, be before doing your. Yeah, in vitro essay, for example, with regard to surface area or surface composition or also aggregation. So things like that. Might also interfere with with your final read out. And of course, also colorimetric interference is also an issue, which is quite important to take into account in a number of inventories.

Paul Whaley
Let me just.

Participant 6
But the number of cells should be equally distributed in deeds. I mean, if you are experienced, I think that would not be a problem anymore. A student might indeed have half the plate half Ethel with cells and the rest of the place completely full, maybe, but or a lot of differences in number of cells but well, but I think if you that that should not be an issue that much anymore. And if there is normalisation against number of cells then. My turn reduce the confining. Go for it.

Paul Whaley
So the ohh specials we actually have started talk about sorry, so otherwise there can be different in price differences and then we had the time varying issue again. So you can potentially have confounders that change over time. The thoroughly difficult to deal with. We talked a little bit about how the time at which the exposure occurs can be a confounder. All of their other ways other than the time at which. The exposure is administered can potentially distort the results of an invitro study.

Participant 6
I think it was mentioned already the evaporation from one well to the. Other. Just mentioned again.

Paul Whaley
Could you have differential rates of evaporation between? Like different high doses would or lower doses. And would that be something that could distort? So distortion because of a change in exposure over time over the course of the. Experiment.

Participant 6
Yes, because evaporation will be in in those from high to low concentration. So you would say that the higher the highest concentration and the lower the lowest growth strategy in the next. Now it doesn't matter. Now I think it will go just in this space. So I don't think it matters that much on the scale of a plate. I don't know. I just thinking out loud but.

Participant 1
Yeah.

Paul Whaley
Can you get? Is there anything like so like weird that happened so some of the things that. Have been observed happening in animal studies that are very surprising and strange, but end up being. Kind of distorting effects in the studies that. Animals nearer the ceiling so in. Housing. End up being slightly warmer because they close to light than animals on a bottom shelf, so if they're not randomised to even pose, so you have to randomise them to position in housing in order to ensure that you don't have like differential environmental effects. A play particularly very sensitive like assays is that something that can potentially happen within vitro like in, I know if you keeping things in incubators or fridges or whatever temperature control environment. All I controlled or?

Participant 1
And then I think it's one, the one you mentioned about the edge of it, for example in some assets, it's important not to fill in with cells and the test items, the edge of the plate because it evaporates quicker and then. Also, I think randomization is really important, so it should be important to, especially when you are testing many test items, when you repeat, when you do the. The replicates it's. It's important to to randomise its time, so to have its test time terminate different position in every experiment.

Paul Whaley
What sort of randomization techniques do you see being used? And in vitro studies. This is it. Do you randomise across the well?

Participant 1
Yes, yes, yes.

Paul Whaley
And if you do, how do you keep track of which?

Participant 1
I think it's important to randomise between the worlds. We go most of the carnival. You can avoid several problems like for example if there is. The contamination, for example, from the positive control. It's important to. To avoid. Consistently. Reproducing the same mistake, let's say it's important that you. Of different wells in its test item. So you are sure that what you're getting is. It's not because of the position of the this chemical, but it's a true response.

Paul Whaley
Hmm.

Participant 6
But is not generally applied. I think it's not the default to randomise I guess, or is it?

Participant 5
No, I don't think so.

Participant 6
That shook maybe become a default, but uh. But.

Paul Whaley
So might be interesting just because we've got 10 minutes left, it's going to be difficult to do another domain meaningfully, but maybe we can just concentrate on this concept of randomization and how that might work. Because I cause I don't. You know I'm not. I'm a methodologist. I don't do experimental research. It's probably obvious. If you have like a. Multi wild plates. How would you randomise the wells? Like you'd be some labelling system that you'd use that would it make professing a challenge? But I have to return from a practical perspective, what you'd be looking for in effective randomization. When it comes to invitro study design.

Participant 1
I think there are auto automatized procedures through Excel I think, or I don't know which other programmes, but I think I think it's an automated. Ohh function of Excel for example.

Paul Whaley
But how would when you actually try to do the? So you have the randomization, but then when it comes to an investigator actually professing out. Like dices and things. Would that I just wondering cause to my knowledge it's not very common for randomization to be done in in vitro study designs, but like I've never surveyed it so I wouldn't know exactly how common it is. But it was drawing. It might be quite challenging if you've got randomization on and multi world play. And then you're trying to pet out different doses in these different wells. And then maybe there's potential for error. How would you know which well to put it in? I guess you'd have a grid arrangement or something, but?

Participant 5
I think it's. I think it's more to avoid cross contamination than actually to have a. Randomised setup.

Participant 6
But how would you pipe at it? And and and buy with a robot. It works, but indeed 96 well plates. Bye bye by pitting randomly. I have not been in a lab for a long time, but I'm not sure. It sounds like a. Yeah.

Participant 5
Ohh I think I think I think that can create a lot of mistakes. Also if you putting it two randomised you will do mistakes when when you're doing exposure so. But to to have um. The controls in separate plates. I think that's most important. And if you and if you work with different compounds, then in the same experiments to ensure that you avoid cross contamination between the worlds.

But there's another time varying confounding factor and and that. Especially when you work with particles then that they can and on the particles that they can aggregate and and then sediment or so you you should ensure also that actually the test compounds gets in contact with the cells. Um particles, for instance, are nano particles taken. They can also flow just on top. Or the media not actually reaching the cells if you're working with the tyrant cells, then on the bottom so. So this is also important then and they cannot change size over time. By aggregating so and then change toxicity.

Participant 4
And to follow up on that, I think that the quantitative individual interview extrapolation is really crucial. So to have your cell model properly characterised, to know the protein and lipid content, so that the modellers can then predict how the chemical will behave if it will really go into cells or if you have the full composition of your media, if it will, then I don't know, aggregate in the media or then add soap on some serum proteins or liquids or if then you need to know. From which material is you're well played. If the chemical will adhere on the on the borders or on the walls of the of the well plate or anything. So this is crucial as well.

Paul Whaley
Very good.

Participant 6
Maybe one last one to add to mixing And yeah, so a lot of, yeah, encountered in the past, a lot of mixing challenges some person. Don't mix enough or. Then they know just later on you get strange results. You you correct for it, but it can can lead to strange results if you don't for tax your chemical before you. Start your delusions or yeah, I don't know if the the storing can make a difference. Stirring first for nano particles that will make a difference. Of course, if you still are not stir if they can float or not float. So any mixing challenges that can be a lot that influence your results.

Paul Whaley
Excellent. Thank you.

Right. So 25 past the hour was an excellent discussion. Really appreciate your energy and enthusiasm. Everybody spoke a lot, which is tremendous. Um, we will be reconvening again. What time is the call tomorrow?

### Focus group 1, meeting 2

Paul Whaley
And that would be great. So I've done the recording cheque. We can get straight into detection bias. So this was the most populous domain we found approximately 80 different criteria for detection bias being put forward in the assessment tools that we have reviewed.

Detection bus we define as a bias due to distortions in any process involved. The determination of the recorded values for a variable. Quite broad. Might explain why we have so many criteria for it. So detection bias would relate to things like recording the value for an outcome, recording the value for an exposure or. Really recording the value for any battery more and the ways in which detection bias can be in play can be quite varied. But what I first want to do is ask you. What this means to you and how? Distortions can be introduced into the results of a study through the way in which variables are measured.

So, shall I put someone on the spot? Anyone like to jump in with a comment? Remember, there are no incorrect comments. It's just a discovery process. If you have a question about the definition, just ask.

Participant 4
So do you mean if somebody incorrectly recorded the values, meaning that they made a mistake in the Excel file and put the result in a wrong cell for example, which would be prevented by having an Excel templates for instance.

Paul Whaley
That could be one way in which. Detection bias could occur, I would think, yeah, because that's about how the data are recorded, right? It can have to do with how things are detected as well, so I think like misunderstanding limits of detection and things in a. Study can result in detection bias issues as well. For example, see that with censoring of data in adversely, so continue to detection of our exposure. So you could be mismeasuring or researchers could be mismeasuring. The amount of exposure that is present, for example, you could be mismeasuring the. Say cellular response to the exposure in some way. I think control groups also seem to be very important for detection bias, because you need to. There are positive and negative controls or essential for actually determining whether an effect is present at all, right. Without a comparison group, you don't know what you're detecting. And then I'm asking it outcome assesses. Also seems to be something that comes up a lot in the tools that we have been reviewing. Whereby an investigator's perception of what they ought to be saying can sometimes affect what they do. See, for example.

So have any thoughts or comments on this?

Participant 6

Yeah, maybe. Well, just some examples. The range in which you can detect. So sometimes, particularly else you mess, you can. U MB NA range that that that actually had the plateau at the maximum level of the machine and people not always noticed that and yeah the influence of other chemicals on your detection. And can we an issue but as already mentioned I think by you. Yeah, so interactions and yesterday we mentioned it already, the interactions of chemicals by themselves with the analysis that people think it's a positive response, but it's just well fluorescence of the chemical or quenching of fluorescence or any other interference.

Paul Whaley
Participant 2 you look like you want to say something.

Participant 2

I was wondering if you would also take into account the solubility. Which it's also important. Um, yeah, or might change the the available concentration in in your in vitro essay. And if this is not properly assessed can result in false negatives. But I'm not exactly sure of this really fits here.

Paul Whaley
It doesn't matter if it's somewhere else, we'll figure it out. Say.

Participant 6

And the same with solubility is the same as sticking to everything to the plastic state. You don't detect the concentration that you want to, or you're thinking you're detecting. Same artefacts there, I think, yeah.

Participant 5

And the same with the small particles, them with them actually settlement on on the cells. If you work with on the other cells so that. Don't reach the cells.

Paul Whaley
So at least I could you just say that again very quickly or just you just didn't come up very clearly on the mic.

Participant 5

Yeah. No, no, I mean in the same. Many in, along with the same role. Actually, it's if you're working with the non the particles for instance, they might not sediment. On top of this, I mean if you put them to the media, they may not sediment down. If you work with the Terrence, sell some of them. So you need to ensure that they get in contact with the cells then too. Be able too mushroom affect. Same with the solubility or if it's sticking to the walls and distance. And and also as I said with doses, of course, it's important to to measure or to test high enough those system. If you test too low doses, you might not see any toxic effect. So it should be sorting. Management, those range.

Paul Whaley
Are there any issues specific to detection of outcome? Cause I as I understand it in vitro research it can be quite complex in terms of outcome detection. So what opportunities are there potentially for error in outcome detection in in vitro environment?

Participant 1

Maybe. Maybe also could be for example a the the solvent. The selection of the solvent. If for example the solvent has an effect on the endpoint that we measure. And for example, ethanol has a if if used as a solvent. For example, in MCF 7 sells it have estrogenic effect and if you are measuring for example a proliferation in MCF 7 cells that shows like estrogenic activity, then and then you compare with this control with which involves the.If you if you compare with the solubility control, let's say.And with the solvent control, sorry, then the result will not be what it should be, so interference of the of the solvent basically.

Participant 4

And I wanted to mention interferences with media components like fennel, red for example, or many, many others, and also somebody mentioned the range of concentrations of even not preaching the plateau, but also being in the middle, ideally in the middle of if you have a standard curve with some standard compound then you should be in the middle of the range and not too high or too low, because then it wouldn't be so accurate the measurement. . Of you have to come.

Paul Whaley
Interesting. OK, good. I'll make sure we capture that. What role would masking of outcome assessors potentially play in in vitro research? Because it can be quite important then say. Histopathology slides like if we're looking at, you know, slicing and organ up, but it would obviously work differently with invitro research. Do you have any sense of how the? The investigator's perceptions of what's going on in the play could affect the. Results that are recorded.

Participant 6

I think that this lesson less I mean nowadays. Most um results in vital results are screens by computer or machine and not by individual manual scoring. Although there are some OCD protocols, it's scoring manual scores. That's Yeah. So there are a few, but yeah, I think the the new method generally tend to. To automatic scoring. Which has the less of influence of the investigators on the. On the result. But if people still work with manual scoring, would you like to hear that as well? If that still happens?

Paul Whaley
Interesting. So didn't want to respond to that.

Participant 3

You doesn't it really relate to whether there's any borderline calls, either by a computer or you know if you're manually scoring? If it's a clear, you know it's just, it's a question of whether clear positive is distinct from a clear negative. That that's when it. That's when it would impact I think. Great.

Participant 6
But in case of a human, and if the person knows that what research is about and you want to see your certain effect, you might be a little bit more biassed than a computer towards, but then, on the other hand, you can also set the limits of sensitivity in a computer lower so that you can still see an effect, so you can still building computer analysis. You also, if you want an effect, you can still. Look for it by setting it more sensitive, so I agree with you, Participant 3. That's in the end, it doesn't matter. Maybe if it's a human or. Computer it's, it's these. Yeah, the the the problem lies in these border line. Ohh I should call the responses that you don't know. If it's true or falls or not, yeah.

Paul Whaley
Interesting. So maybe so this is something that didn't really come up in the included instrument included assessment tools, but would it be possible to expand on this idea of like these borderline judgments? So if someone could explain. What's happening with the borderline judgement, and then how it's significant and the implications that can have the results or distortion the results of a study? This could be important, then, for us picking up ideas that we can put into the assessment, all that we create.

Participant 6
Well, there have been. I don't know if that's that's an example of a borderline discussion, but there have been a very any discussions on on non monotonic dosethose response curves or people see effects in. Background I would say ohh that's my opinion. Maybe you're ready. But I have not looked into the subject, but are people claiming to see effects in borderline cases where others say, well, that's just background and it should not be considered an effect? And I think yeah, this called the non monotonic. Totally don't. More no more now. Can someone help me on non mono?

Participant 3
Nonmonotonic.

Participant 6
So like, yeah, that's it. Those response care um where you can have an effect on conclusions using back, yeah. Uncertainties in in in responses.

Participant 2
Yeah, maybe I can just add an example from the OECD test guideline programme when they were these, the defined approaches for skin sensitization. There you have a combination of three different invites, chemical and in vitro assays and. They are for each of the three essays. There was really a huge discussion on defining these borderline ranges in which each. Now in which one of the essays? Where result in non conclusive. Ohh statements.

Paul Whaley
So just listening, so it sounds like some of this is to do with differentiation of signal from background noise. If I understand correctly. I'm getting some nods, so I'll take that as a yes. It's some of this to do with the conversion of continuous. Variables into categorical because it sounds a little bit like. Sometimes you're saying, well, there we've got some numbers here and then this is a signal and this is not a signal.

Participant 2
Yeah.

Paul Whaley
So. Is it potentially problematic this this this process of interpreting continuous data into character the coracle? Data. Back presents some problems, presumably.
Because you're turning kind of subtle signals into yes or no kind of values. Again, I would just say you probably shouldn't not because we can't pick that up on the audio.

Participant 3
Paul, I think it's the fundamental problem with epidemiological studies. You know in, in different exposure categories actually. So it's it's having to divide. Put it into yes, no categories. That I that's that's. That's where bias, you know, impacts. It's on these borderline calls, so it's it's. I think it's a combination of things, it's it's biassed, but it's also. And this having to take continuous data and put it in discrete categories. Which is the fundamental basis of most of the results.

Participant 6
But ideally, most in vitro assays are not or should not provide categories as outcome, but an easy and affect those. So and and and fit of the dose response curve and then. And easy 10, that's results in less bias. You still can talk about the background and when is the response above the background, but it's less of a yes no answer that you get a bit more of a continuous outcome that you get. Although maybe that will be put in categories as well. In the end I don't know. That's the point.

Participant 2
Yeah, that's the point. And that's really the point. At the end, you have to put it in categories and especially when you have to differentiate between. Uh, no medium and strong effects. It's often very critical to critical to define the right borders, , especially for the middle category, because otherwise you either would have. Yeah, 5. Faults assignment. Yeah, that that the low category of falsely assigned as medium or that the medium are indicated as higher. So it's always. Along discussion to really define the borders for their for, especially for the medium category, and is also important when you set up your model that you have the right range of reference chemicals addressing all the different categories you want to assign later on..

Participant 1
Yeah, I agree very much with what the participant 2 said. They think that they in this is really part of the the data interpretation procedure, which should be really clear. The data interpretation procedure for its asset, let's say. And then it depends also very much on them on the reference chemical. So it's really important that source we said yesterday that it's really important to have like week agonist, medium potency agonist, strong agonist so it's. Helps to define them. Transplant let's say between categories.

Paul Whaley
OK. So then when we're talking about defining thresholds, thing is this, is this a applicable to particular subcategory of like in vitro research likes kind of like risk assessment relevant or kind of like applied if you like versus what might be more typical lab based work where there's less pressure maybe to categorise like is there a different need to categorise in some circumstances versus others? I guess is what I'm trying to say. You shouldn't know.

Participant 6
I think you said you know challenge with the use of invitro data. Now that is generally used for, I don't know hazard characterization or some some yeah, put them into boxes kind of. Whereas I do think that if you. But that in vitro data is not used for that yet. Um. For risk assessment that you would also extrapolate these in vitro concentrations, then you will get into more the continuous data. But yeah, it's just that. At this point, we are only. Using it to categorise. So there might be different, yeah. Uses for in vitro data that that or other uses of in vitro data that may less depend on categorization and more on. I don't know relative comparisons or high versus low, but. But yeah, they're putting it into boxes. I don't know. Apparently what I hear, there's a lot of use of that, that that immature data used to do that whereas yeah, this problem will last appear if you would use it more. That the continuous data more..

Paul Whaley
There were some issues that came up for more detailed discussion. So we did quite all discussion of confounding covariates yesterday. Though she's of detection of confounders. I'm just wondering. If anyone has any thoughts on that in addition to what we were talking about. So this would be about. It's very hard to adjust for a confounder if if you don't know. But if you don't measure the compounding variable in some way so their detection bias issues around confounding covariate measuring that then have implications for how you adjust for confounders. Does that ring any bells for anybody? Okay. Then there's time of exposure being a detection bias issue, so there's a lot of different language around. Timing in each of the across the tools that we. Included in our review. So people talk about time points, they talk about Windows, they talk about duration of exposure. They talk about latency periods. They talk about periods of exposure. The language isn't particularly consistent or particularly easy to understand. So I'm just wondering if. If this means anything in terms of like you know there's a wave like talking about specific. Time points of exposure or like a window exposure that's relevant in a study and just what sort of language that you think is most applicable here.

Participant 1
Maybe here also for example I passed the number of passengers of the sales is relevant because I think it should be. It is important to use if you want to have reproducible results to have. To use for example, the same 222 define the rains of. Alpha. Passing that you can use for certain cell line. For example, from passwords up to five passes, 20 for example.

Paul Whaley
OK, that sounds good. In terms of. Let's say image analysis and data visualisation techniques. I wonder because it sounds like a lot of this is automated. But could there be issues with machine calibration and things that we should be aware of? As potential distorting factors in.
In the. Whether in vitro studies being done.

Participant 4
Jumping um about 3D cell models, or organoids. So if you are using image analysis, sometimes it's really difficult to focus since it's 3D. It's not a monolayer as with monolayer cell culture. And so yeah, it's harder and some.

Participant 4
People can well may not focus. Right. Sometimes it's even not not possible, yeah.

Participant 2
Yeah. Also to add on this for such type of analysis and not only three, but also um other cell models. For example, when you're just looking at a typical morphological changes as outcome of your say you need a training of your analysis software. So and yeah, this can also introduce a bias then if if it's not appropriately trained.

Paul Whaley
Okay so this is an issue where the move away from manual methods to machine methods. There's still potential for a systematic error to creep in, so whilst you. You don't necessarily need to mask people to the sample. The machine itself is still plenty of room for. Liking stakes, and we need to understand what's up. OK, that's very good. The other thing that came up which we didn't understand what it was referring to, was this concept of market cut off points. There was mentioned in several assessment tools, but it wasn't clear what they meant. Does anyone have any thoughts or comments on what this could be? Hit this relay.

Participant 6
Do you know your phone text? Why? Why?

Paul Whaley
Not really, it was variable. I think it might relate to the discussion we just had of when you've got some sort of. Level of response and you're saying that this is a positive response and this isn't so you kind of converting continuous data to categorical probably. I suspect that that might be set too low or too high, so you end up with false positives and false negatives consequently, but.
Okay delong detection bias. We will spare you anymore of that. We can move on to early study termination bias, which had 0 criteria and. We know the substantial an issue, so we define early study termination bias as a bias due to the decision to end the study earlier than planned. So it's a little bit like a data censoring exercise, so you can so in in like the classic would be to a clinical trial in humans, you give them a drug, the drug looks like it's working. So you stop the trial whilst it still looks like it's working because you're worried that if you continue the trial until it's complete, it'll stop looking quite so good, right? So I think in our context you could have either an early study termination or a late study termination by. So you keep doing the study until you see something that it looks like it's good again. But I'm just wondering if this has any meaning in the in vitro context or not.

Participant 1
Yeah. For example, if they wellssales are contaminated, you stop the experiment. If the dog is that you are using are tools at the toxic, then you stop the experiment.

Participant 6
But then you wouldn't not publish the OR report you. Yeah, I don't know. It's not. You will not show the results and say, well, I stopped it just because then I gotta terminate contamination. And I showed the results up to that contamination. You generally start over again without the contamination. Contamination and. Yeah, but yeah, so you repeat the experiment. It's not like you showed results. You might note down that. It was an issue and that's why you repeated the experiment, but it's not that you showed results up to the level that you see sided toxicity or. Suddenly you had a contamination and you sure everything up to that point. As with the example of an human study.

Participant 4
I think it can be also related to.

Participant 4
When you do some do a first independent repetition of a test and you see that the chemical just doesn't causing any effect and you think that it's so. So good looking that you won't continue doing the other two repetitions and then we just stop it, which doesn't make sense because obviously anything might have happened and you might see results in the next independent repetition. So always doing 3 at least.

Paul Whaley
So I was going to ask if there are any kind of in vitro studies that maybe imagine. That I know what I'm talking about, but imagine you have like 20 plates or something, or multiwell plates and you kind of do the 1st 15 and you like, who've got a result now and it'll be another week's work to do the last five so. I got it. We're done. And then you stop and you publish. Is that something that could happen? Obviously not in your laps, but could happen.

Participant 1
I think it could happen. For example if the if the positive control is working. If the negative control is working, if all the relevant controls are working and your test items have found two times to be completely negative, then. Maybe there is no need to proceed to a third one. Of course the correct practise is to do to to perform a third independent, but I think that if all the controls are working properly then. And you still have negative results in two rounds then I think then that. Many people would stop in the two rounds.

Paul Whaley
So it's 3A magic number here as well. Is that based on a thing or is that just practise?

Participant 5
I think that's the standard thing.

Paul Whaley
Standard.

Participant 6
But it might be too much.

Participant 5
Yeah, you you do, you do to repeat some experiments or in total three independent experiments. And. That that's sort of. Tambuwal for in vitro studies.

Participant 2
But normally you have that also technical replicates and and you have biological replicates which both often or should be 3.

Paul Whaley
And.

Participant 6
But the earth can be arguments. I mean it's it's considered the holy grilled 3, but I think 2 can be just as valid in a way statistically as three, because often variation is not that high that you would need three. So there might also be an historic background on that. It's good to have three, but it should not always be. Ignored if the results are too, they can just be as good as well, but indeed, as some people have different definitions. So I I remember people doing 3 experiments or one experiment and analyse it three times. That's the analytical repetition. I'm like, yeah, but that's not. Well, then you have your three, but it's another 3 then. Then it's meant with the three.

Paul Whaley
So how much is one of the things that we worry about with particular animal studies? Is is powering a study right? So. Quite often one of the issues with Preclinical Research is that it's done in sort of half a dozen or dozen experimental units, and that's probably not enough experimental units to power study for the effect size that is being looked for, like you need a much larger. Number of units in order to to reliably to take effect and trying to see is how much power calculation work is typical in in vitro studies. I mean, and I guess also what's the is there much analogy between say having 20 experimental units, maybe litters or individuals in an animal study and then having, you know, whatever the equivalent of experimental units is an in vitro study? And though we talked about biological technical replicates, but. I'm just wondering.

Participant 4
No, this really depends on the essay. This really depends on the essay and how DSA is work and how the cell model is reacting, because for some it may be fine to have 5 replicates and for some and points we need to have 20 of them because the variability is higher because some experiments can take one month instead of five days. And then of course the sales have more time also to behave differently, so it really needs to be. And you're fit for purpose with your historical data. But the minimum there is a minimum that needs to be there. Yeah, sorry.

Participant 6
Ohh yeah, I was just thinking if you need 20 replicate biological replicates, let's say for one data point that sounds. Um, well, not a good essay, but maybe there are examples that it's still a good essay, but it just sounds very friable and and what what uses the outcome then anyway, if you need 20 replicas because people using replicates also to make their data look better, just the more replicates the yeah, the higher chance they will. Have to confirm our to masks on some variation or I don't know. So on the other side, too many replicates is also a sign of hey, what's going on here? Why so many replicates were needed to to see an effect?

Participant 1
Exactly.

Paul Whaley
So is there something about the mode or type of research there then that's significant because to my naive understanding, if you have a test system where there's more variance than you do more replicates so you get a, you know you get a clearer sense of where the average is and then the ranges across that average, it will be normal with, say people, animal studies that you'd expect quite a lot of variants. So you'd do enough replicates hopefully accordingly, but within vitro. Studies. Is it always important that the essay is kind of like, you know, good enough that you only need to run it three times, or could there be experimental contexts in which? Someone. Has say it has got a fair degree of variance for whatever reason and therefore should. Um, do more replicates and that's okay and that would be just good research, right? Does that make sense?

Participant 4
Only if the test doesn't fulfil your criteria. So if you're positive control is not in the correct range or negative control is behaving wrong, then you need to discard this data because they didn't fulfil the criteria. But otherwise you cannot just do more experiments to fit to get better data.

Paul Whaley
No, of course. I mean, that's not really what we're like. Yeah, okay good. So. There is a sense to then of early study termination or particularly late. Repeating and repeating in order to get the result that you're looking for. That's interesting okay. Good. Does anyone else have any final?

Participant 1
And and then and could. Could I add that maybe there is also an for example in? Bias. For example, if you are overpowering. Your your your data, so if you have so many. And replicates in so many.
Ah, data, then you might, for example, if you're in, for example is 1000 or you're in is 20, then there is this danger of overpowering, then very small differences might seem significant, might seem. Music and might. I am trying out to be so statistically significant if you are. If you're N is high. Really high where in other cases where the end is. A smaller number then this small difference is are not detectable through your statistical analysis.

Participant 4
While then then you should lower the N if the variance is not well. If there is no reason for having more and. Yeah.

Paul Whaley
OK. It. Thank you. Right. Let's move on to performance bias. So we had this is quite a again some of the came up quite often. 44 different criteria across the 72 tools that we included on a review. Define performance bias as a biassed resulting from differences between the received exposure and the intended exposure. It's quite complex. And there's a lot under the hood in this definition, so the exposure isn't just the test substance that is administered. It is the all the things that are happening to the. Um experimental unit in the experiment, right? So the received exposure includes like the temperature which is kept. So they. Read that it's bad. You know, it's a rap meeting that it's kept in the vehicle and all these other things, right? So that all the stuff that is done that are the variables that are supposed to be kept consistent between the experimental groups, including then also the variables of interest that are changed between those groups. That makes sense. So you have the received exposure. That's what they actually receive and the intended exposure, which was the conditions are intended to be in. Says only have thoughts or comments on what this means to them and the in vitro context when it comes to distorting potentially the results of a study.

Participant 1
If I may, I think that usually in in vitro studies, we don't measure them. We don't, we don't do analytical measurements to see actually what is the concentration. In the medium, for example, this is most common in the ECHO eco toxicological studies. I think it's not the case in in vitro studies.

Participant 5
I think it's the case.

Participant 6
But as mentioned, okay yeah, go ahead.

Participant 5
And for nanomaterials, it's important also for in metro studies. Actually matter of taking selfies?

Participant 6
I think you'll agree with the nano particles, but also for all the chemicals, I mean there are discussions about thatthat. It's not use and normally the exposure is not tested, but we have already mentioned in the other topics that there are many.

Participant 2
No.

Participant 6
Factors that can influence your concentration and.

Participant 2
And.

Participant 6
It would be good.

Participant 2
And also the solubility issue would then know fit here as well.

Participant 5
You.

Paul Whaley
Yeah. So it's interesting you say that so. Some of the specific examples that come through the tools that we've been looking at include the culture conditions, so medium and maintenance and other things. Some of the kind of things I really want to get into is like investigators. So investigate knowledge of exposure groups. So how that might affect, say, the way in which the investigators maintain the sales, for example cell cultures. So we know from. Animal studies, for example, that if the investigators know that this particular. Group of rats and this cage have received like the carcinogen or something. Then I'm going to feel quite sympathetic to those rats. And then look after them better. But then the there's a difference between the received exposure and the intended exposure because the received exposure suddenly includes improved animal husbandry. Ohh, so then the rats are better treated and then like attenuates the effect of the carcinogen in those rats and you get an underestimate of effect size. Are there any ways in which investigates and knowledge of exposure group in in vitro context could result in? I guess some some way in which the the cell cultures are handled systematically differently to maybe the unexposed or the more exposed. They're obviously not as key to the rats, so.

Participant 4
Since all the concentrations are usually on the same plate, you cannot really treat them different like I would say, but there must be some some possibilities. How to induce bias but.

Participant 6
Must be really hard. Yeah, you retro.

Participant 4
I have an idea. Maybe when you are when you. When you. So if, let's imagine that you have multiple well plates and you need to take them out of the incubator to measure them. And you always keep the highest exposure as the last well played to measure. But if you take the mall outside of the incubator at the same time, the highest exposure will be at lower temperature outside for a longer time. So this could induce bias. But. You normally you shouldn't. You should take them one by one for example, but this could be another, yeah. Source of BIOS maybe?

Paul Whaley
That's interesting. So this is set in conversation to me a little while ago that. Racking up things in.
Thank you. You always put, you know, always put this trail on the top and this trail on the bottom. And there are subtle differences in temperature, and then that can potentially cause effects that you weren't intending to happen. Okay, that's interesting, I hope I. So there's a huge amount of concern in the tools that we have had in our review about error in the test substance itself. So there's lots of like, you know, right down the cast number, this kind of thing. How does it actually come to pass that someone had ministers completely the wrong chemical? And and then vitro essay. And how would they prove that they hadn't done? Just out of interest. You not get imagine getting it wrong because I'm useless but. Pick a room.

Participant 2
Yeah, there was one example also for over an OECD test guideline on, I think it was in the aims test that was the name and the cast number didn't fit. So they had to cheque quite a lot of years afterwards. If the chemical name was the correct one, or if the cast number. What was the correct one? Which? Yeah. So it was hard to assign the correct positive control again to the essay.

Paul Whaley
But besides, like you know, inconsistencies and reporting of a name for something, right, so CAS is a name and the way that words are a name. What would the researcher need to do to reassure you that they had applied the correct substance?

Participant 6
I have an example where they didn't apply the correct some substance, but they reported everything so it does not that you need so they reported an Mr. They reported Lcms everything matched but the Ohh group was on the wrong side so they should have had a yellow liquid but they reported a white. Powder and there from that I could see that they had the wrong one. Very subtle and yeah, they could have not known based on their data because yeah, everything was very different. But I only noticed because they reported a white powder. But yeah, it's hardly happening. I mean you you order your chemicals and this was a metabolite, so it would happen more if you synthesise your own chemicals, then you have to report the NMR and everything and do the proper tests. And in this case, apparently there was not done properly. So they got the wrong substance. But normally order substances, substances, so that should provide sufficient. Quality assurance and their yeah, you you probably have to report where you know you at least have to report where you bought it. But you don't have to report always the quality cheques. That was done by the suppliers, but. Yeah, the supplier needs to do this. And yeah, if there's. I don't think there are any reports of a mismatch in that system. Only when you start synthesising yourself chemicals then errors can happen.

Paul Whaley
So it's reporting the source of the chemical, the kind of provenance, I guess, of it. If you bought it, if you synthesise it, then you report the I guess first Cam. Prophecies and then that allows the reader of the study. Can cross cheque the. Discount properties with the state of identity, substance and that helps people detect if there's an issue. Is that right?

Participant 6
Yeah. Tickler and Mrs. So the NMR scan should provide information, but I don't know many people I can read, and a Mars can. So in the end, you can publish, but it's very difficult to cheque. Yeah. So.

Paul Whaley
It's still important to know, I think. Yeah. So that's very interesting. Thank you. Yeah. OK, that's really good. But sorry.

Participant 4
Um, I have another idea. So sometimes it's also good to write down on the storage of the desktop substance if it was frozen or anything else. . And also I had an idea about some unintentional bias. If you think that you buy a specific inhibitor to something and it's not really specific and you don't know about it and there might be some crosstalk between different signalling pathways, etcetera. So we picked the wrong substance or don't know that there might be some hidden effects. And something else.

Paul Whaley
So impurities in test substance. Obviously these can be an issue. Most of the tools that we had. Just sort of said the the substance that should be 98% pure and that was kind of their criteria. I'm just wondering. You know, if that's really sufficient. Ohh, and what's actually going on with impurities that. You would want to investigate and understand or have reported to you or something and being related to being confident that the. Test articles pure enough. That you're not saying it's like significant bias and the results of the study?

Participant 6
But generally accept the impurities, but depending on how sensitive I mean chemical analysts domal impurities, they want 99 common 9% purity. But for many talks essays well. 80% will be OK as well. I I don't know. Maybe some others have examples of the 20% of impurity could interfere. Maybe it works. Speaks starts more problematic. OK, sorry. Yeah.

Participant 2
Yeah, definitely. Um, 80% purity. Now you will not be sure that when you're observe an effect in your essay that it's really. It introduced by the mater compound and not by the impurities, but the most important thing is that you report your impurities. Or or purities of the reference chemicals, um in your analysis report so that. You you have the chance to be aware of lighter on that that maybe some cost checking is needed. Ohh to ensure that the effect was not caused by the impurity contained.

Paul Whaley
So what would be involved in the purity cheques that would typically take place then cuz? Sounds like you know 80 to 9 suffered that. That's sounds. Sort of intuitively quite low. And then where we are we expecting the research labs who are doing? The in vitro work. To be checking the purity themselves, or is this information it's reported by the manufacturer of the test article or?

Participant 6
3.

Participant 2
Yeah, would rather some information to be provided by by the supplier normally at least.

Participant 5
But I agree with what you said, that it's very important to report it in the papers and I think this is something that is quite often not reported.

Paul Whaley
Is there any? Systems in place where, presumably because we have the information I'm guessing have information about the where it was through the supply, was and what the state of purity was. It is there any kind of goatee resources for checking batch purity say later. So if someone's saying okay alright, we've got this study we're trying to assess potential for distortion results. You know, we've got this state of purity. What does one do for go about for like trying to evaluate whether that purity is too too low? In terms of potentially distorting results or like cross tracking that that was actually the purity and it wasn't in fact say lower, is there anything around that that happens or information can be drawn?

Participant 1
Probably there should be proper storage of stocks showing in case needed to get can retrospectively go back and cheque. Then. The concentration of your active substance thing.

Participant 6
But yeah, you cannot unlimited the store stocks, so that's they will maybe a few months. But for the time needed to confirm things, maybe then then often you notice that the stock was not good.
As well anymore.

Paul Whaley
Okay, I'm gonna put PARTICIPANT 3 on this bottle because Participant 3 has not said much for a bit. Just want to make sure the microphone still working nicely.

Participant 3
Yeah, the microphone still working, so I haven't added anything where I didn't think it added any value but. And this test purity is, I think, of these things more in the context of how we apply the data and test purity. We I think it is important to underscore, we really need the information because depending upon how you're using it and what you're looking at. Um, it's up to the those using it as well to interpret that information in terms of the purity of the compound that they that they they're looking for for a particular application. So I know when we're doing mixtures assessments we've actually. You know, rejig the mixtures assessment based on the purity of the compounds tested in the in the in the in the studies. So you know, I think the investigators can only really rely on what's provided by the companies. And but it needs to be reported. It's really, really critical.

Paul Whaley
Okay, thank you. I think I cut was it. Participant 5 or Participant 2, who I cut somebody off bringing Participant 3 in, so I apologise for that. And if you'd like to.

Participant 4
Yeah, I want. I wanted to mention that sometimes also the freeze thaw cycles are welcome deteriorate deteriorate the quality of the compound, but it's never reported actually in the papers. How many times do you thought, yeah.

Paul Whaley
Interesting. OK. That's very interesting. Thank you. Right. So we will keep moving on in the interests of time. Did that's. That's worried about that. The other group, OK. So this is entirely your fault. You brought this up yesterday. We have predictive model research bias. So begins with P, which lights coming up now. So there was yesterday some discussion of the difference between. Research that is done for descriptive purposes versus research that has done for predictive purposes. This is not a domain, which is. I think particularly well understood in our space, so the source that we're using for defining the domains currently defines predictive model research bias as a bias specific to the design conduct. Now, is this all reporting of research about predictive modelling? So it's quite broad. I just wondering, bearing in mind what we talked about yesterday, if anybody had anything additional they wanted to say about in vitro research that has done for predictive. Purposes, I guess. Or modelling.
And how? Distortions can be introduced into the results or interpretation of that kind of work.

Participant 4
So I think that it's. So we are using actually uh predictive models and it's really crucial to describe actually to have a separate publication just describing how the model works. So there are specific parameters that we addressed, but I don't know if I can just send you the paper.

Paul Whaley
That might be useful.

Participant 4
It would just take time.

Paul Whaley
I think we're not sure how or if we include this, but this is kind of the new area. So it's good to get people's thoughts. I mean for the record, is there any like 3 or 4 things off the top of your head that you'd like to mention now that we have it?

Participant 4
Yeah, but I think we already mentioned some already. So when is robustness, so you need to have essay specific. Ohh. Positive and negative controls and their historical coefficients of variants. Then productivity that is as by specificity and sensitivity. Again how well your predictive model evaluates. If it's really positive compound or a negative compound, if any false ones and relevance of the predictive model. Again, it needs to be fit for purpose. So if you forgot to include any specific cell type that should be there, that would then change the outcome or. Ohh signalling pathway knowledge and yeah. But there might be also more.

Participant 2
Another important issue for this process would also be the reproducibility. So within your laboratory and between laboratories and it's, it's also important that you have some blind testing.

Participant 2
When you assess the reproducibility, yeah. To have an independent evaluation of your predictive model. And yeah, this is called altogether. This is called a validation of vitro methods with not only the reproducibility but also the. The relevance and they have altogether thorough validation of your essay, is needed.

Paul Whaley
OK, very good. So just for the record, I think it might be worth somebody just stating what they? Understand predictive model research to be just so we've got like a verbal. Account of people's interpretation of this concept and then we can use that to cross match. That might be the different understandings will have from different groups, so I might put you on the spot here. So you just say just kind of describe in brief terms, not necessarily too brief how you interpret this kind of predictive model concept.

Participant 4
Okay so it's a model that will predict the outcome of an essay. Ohh yeah, based on the actual in vitro experiment.
So we say again, I just got asked stupid questions, but so when you say it's going to predict the result of an essay, do you mean you're using an in vitro method to predict the outcome of an animal assay or something like that or?

Participant 4
Umm, no, I wouldn't touch up on animals at all here so. Well, it depends. If we are doing human risk assessment then it should be, On the contrary, not at all compared with traditional animal essays, but with human Physiology and comparing, as I mentioned, the signalling pathways or biological applicability domain or to show that it corresponds to, I don't know, human disorders, diseases or anything but human relevance unless we are doing ecotoxicology I don't know where. Are you going with this?

Paul Whaley
Well, human. OK. Can I put someone else on the spot and to? Agree. Disagree. Develop that concept. Maybe Participant 2 would be good for this.

Participant 2
But but you you mentioned the comparison um to the animal experiment. So for quite a lot of in vitro.

Paul Whaley
They forget I said that I wish I hadn't.

Participant 2
No, but I would just wanted to give an example. So I I agree that ideally the human relevance that should be taken into account, but for at least for the first sets of in vitro methods becoming OECD test guidelines. Um, the the the goodness of of your predictive model. Ohh, it's quite often compared to to the older data based on on animal studies. For example for skin sensitization or irritation corrosion. Um, yeah. You always have your set of of reference chemicals and um, they are the sensitivity and specificity of your essay is calculated based on the comparison or to animate data. But of course, as it comes to now, to integrate more new approach methodologies in risk assessment, you have to change somehow also this process. Of evaluating the the predictive capacity of addresses or in vitro test batteries mostly. I don't know if this helps.

Paul Whaley
This is just to get on the record for the transcript purposes. I asked stupid questions to elicit responses to them. Yeah. So I take ownership for anything that comes out of my mouth.

Participant 4
And if I may, I think it's my. It's may also actually be a bias if you compare with traditional animal methods and I don't know how it is foreskin sensitization or any other purposes, but brain development is actually in some aspects different in rodents and human. And if you compare it to the traditional in vivo, it will be different and then it will say ohh your essay, your in vitro essay is not working properly because you are not receiving the same outcome but actually the cell type might not be present there or is developing. Differently or or I don't know, or yeah.

Participant 2
Yeah, perfectly. I totally agree. Um, for DNT, but there's also not much in vivo data available, so there's not much to. To compare that and of course you are right if they are in vivo. As data, of course they are from from rats and. Or mouse and thoughts. It's quite hard to compare or not not really appropriate.

Paul Whaley
Very good, okay. Thank you for that. Right. So reporting bias is Next up. 15 items across our 72. Assessment tools. So reporting bias we define as biassed you to distortions in the selection of all representation of information and study results or research findings. So results are kind of the data that's in the table. The findings are how the data and method are interpreted into. Findings. I guess the import of the study. Selection is about choice of data. See what I have. Different. Outcomes might want to emphasise the most favourable, and representation is something to do with spin, or how something is discussed or represented. Your experience of potential reporting bias in vitro studies distorting results. Have you seen it? What makes you think it's present? So how do you detect it? What would you report to make sure that you had covered everything properly and you weren't being selective? Any reporting of results? How do you react to this kind of definition in the invitro context?

Participant 6
I think the reporting bias happens too often that people. Leave out data historical or. And.
Yeah, the contacts of of of Ohh many historical data. So you have your experiment and they don't compare it to to others or they only compare to one other. And. Results. Um, so it may not be necessary. I don't think it's common practise that people live out data by themselves, but they just need, yeah, in the interpretation it's more the the that a lot of biases that yeah to make your case a little bit better by leaving some. Historical data out. So it's not your own data, but the historical data. And it's very difficult to find. Yeah, to to comment on that. So if you see that people make a comparison, but if you go to the original paper to another paper and then you go to the original other paper, then they also need to two graphs to compare it and left the third out. So apparently that was not not fitting enough or. Um, but again, it's not in the data itself where you see reporting bias, mainly in interpretation of the results. And yeah, that's also part of reporting. Of course. That's how do you frame your results and is that, yeah, do you leave out findings of others, did you? Prefer to ignore or something like that.

Participant 3
Yeah, I always think of the reporting bias not so much as being intentional in terms of generating the data, but it's how you report it. It's always what's your spin on it? What do you highlighting? And I I think that it's really difficult to. Completely eliminate this bias because people. As they write the papers as they interpret the data, have a particular understanding of why they've generated the data and and you know where they're going. So I think. I think this probably occurs more often than not and is very difficult to get a handle on I think.

Paul Whaley
Yeah, I think I should emphasise, none of this is intended to view research in the way that is prejudicial. We're trying to come up with a way of objectively understanding the extent to which any given studies results tend towards the true right. So it's not intended to. Yeah, prejudice. Anybody. So for examples of reporting bias that come up. You know, choice of reported exposures like so maybe there were multiple exposures and then the researchers selected amongst the exposure that administered a set that gives them the best results, right choice of reported outcomes, maybe they're multiple outcomes. I think this might be familiar for some people doing work in underground disruptors or something where there's, you know, 25 outcomes in an essay. Three of them are positive. There's a tendency to report the positive assays, maybe choice of reporters analysis comes up. I think maybe one of the ways that people think is a bit sneakier. So you kind of decide. You do several analysis analysis. You decide which one produced the best result, and that's the one you report. There are other ways in which things can go wrong to these ring a bell for people.

Participant 1
I think it's very difficult, as we said before, to identify when someone has left outside, for example, outliers. In an experiment, and normally there should be like a description for exclusion. On how they on what terms and what basis they are, outliers were excluded, but I think you can rarely find it so.
Unless you have the raw data. Then it's very difficult to. To figure out if there were any outlaw outliers excluded. But the best practise is really to describe the procedure for excluding the outliers. And also from um no, I forgot the other one, sorry. That was what I want to say.

Paul Whaley
That's OK, that's.

Participant 6
When you develop an essay down, it's at one point. Well, most of the trials you don't report, of course. So that's, yeah. Makes sense. But that's that you will not. Yeah. Only in the end report the nicest results that you get in the end because you have done hundreds of trials that gone wrong and you optimise and again went wrong and then you showed the optimised results. So I don't think that's a problem. It's just. The way of research works, but I think yeah, it becomes a problem as soon as you have a fixed as a, particularly in OECD terms, than if you would leave out there data points or or not. But in what we discussed just before that part is missing, then it's not only the data itself, but also the interpretation of the data that yeah, people live out. Uh, sometimes on purpose. Not on, sometimes not on purpose and background information.

Participant 4
And I have three thoughts. One, when people select either standard error of mean or standard deviation, whichever fits better, the standard deviation looks smaller. Etcetera. Instead of choosing the correct one that should be used for data, then those who are forgetting to use correction. And multivariate analysis and statistics. I mean the Bonferroni correction if you have. If you are comparing multiple concentrations for example or you have cascade of of decisions and the third I wanted to mention, yeah, it's also our case actually. So we are working with benchmark concentrations and Vmware's and the question is whether the VMS should be based on all your historical data or on the historical data of all the laboratories together that are using the say or only. On for one project. For example, when there is one experimentation, who is pipetting really well and another project there might be working. Some students who have higher variations so than the BMR would be slightly different. So this is something that definitely can in use by us.

Paul Whaley
OK. Just for the record, what can you just stay? What BMR is so we have here?

Participant 4
Benchmark response.

Paul Whaley
Okay Good. That sounds excellent. Thank you. How's um? Very interesting so. The thing that came up that we were most unsure how to interpret is what is referred to as selective emphasis on post hoc analysis. So I think this comes from. Um, the idea that research where the analysis plan is specified before the data has been collected. Um is potentially less prone to bias than research. Where the analysis plan is decided after the data has been collected, and then you've got some emphasis on the post hoc data in favour of the pre specified data. But I'm just wondering if this, I mean it's a theoretical possibility. I'm worrying if you've encountered it or if this kind of concept means anything to you have anything you'd like to say in response. It is a tricky 1 so you don't have to say anything. Ohh, we can move on. Maybe 2? Okay what is happening? Truncated discussion of selection, but it's just to get some ideas down from you. So we've got a reference point for the next focus group. So here we have about 20 criteria coming from the 72 you included. Assessment tools. So we define selection bias as a bias resulting from methods used to select subjects or data. Factors that influence initial study participation. Or differences between the study sample and the population of Pinterest. So it's like complex bias concept. When we're talking about subjects or participants, obviously we're talking about experimental units because these definitions are written in such a way supposed to be reasonably denied agnostic, but was developed with kind of. Human trial research in mind.
So you're selecting subjects. Into your study or data into your study. You've got factors that influence study participation, so in human trials, you know that might be something like the ability to get to the place where the intervention is administered. In. In vitro research it might be to do with some initial cheques on your. Ohh cell cultures and then you eliminate those cell cultures from participation at the study cause there something that looks wrong with them. Or you could have difference between the sample in your study and the population your sampling from. Ohh, so that would also be a type of selection bias. Ohh, this is quite difficult. Do you have any questions or thoughts as to how this might apply in the invitro context?

Participant 4
I think that the initial control of your cell model that you mentioned was already alluded multiple times with the given document, so that you need to. Yeah, really. Cheque. I don't know. And everything concerning yourself line. I know plasm contamination or karyo types. Or the genetic. Ohh I'm yeah origin and if. Yeah, if everything is correct and everyday morphological assessment and. Yeah, there are some other criteria as well.

Participant 6
And maybe what fits in here as well. That selection bias is that know your biology. So if you are want to know a specific effect you the the system should match that and there are errors that the.
People have selected the role model for the effect that they wanted to you. Um show and this was carcinogenic effects for due to metabolism. So if you don't include the metabolic pathway then yeah you will for sure get the negative results and they wanted to have negative results. So in a way you see that maybe you could also on purposefully necessary but often people don't know that they don't capture the biology but sometimes you could also even do it on purpose for example to. To get the wrong. Yeah, model for effect that you want to to see better. Yeah, it happens.
When people know it or and don't know it.

Participant 4
And this applies also to a presence of receptors in your cell model. If the chemical kind can't bind, then it won't do anything.

Participant 1
Another thing that I was thinking is for example when you have some sub populations of different types of cells and for example you when you start your *** you want to have a specific number of cells in your world for example. But then it may be the case that some sub populations grow slower. And the other subpopulation grows faster, and then it overgrows. So in the final, the final estimate in the final measurement, let's say of yourself, you have. The. In the number of sales that he'll you wish her well. For example. But then it's it may be that the population are not. Representative let's say I wrote. And that represented enough the the subpopulation that you want to. Though that you are interested in so maybe I think that was mentioned yesterday also that's maybe we need to to to measure specific markers. Sorry cell type.

Paul Whaley
Yes, that sounds very good. OK, excellent. Thank you. That brings us to a close we're at the half hour, so I wanted to again thank you for your excellent participation. And enthusiastic comments and. Patients with my silly questions as we try to draw out of you your understanding of what's going on without trying to be too leading in your responses. So yeah, that was really good and I really appreciate it. I don't know if screw you won't say anything if you're. Not a. Is that OK? Great. Well, anyway, I just want to say thank you.

### Focus group 2, meeting 1

Paul Whaley
Our first domain today is detection bias. We found 80 approximately unique instances of. Internal validity criteria for in vitro studies that in some ways seem to pertain to detection bias. Ohh so it was quite a busy domain. We define detection bias as a bias due to distortions in any process involved in the determination of the recorded values for a variable. Say variables and research can be obviously dependent or independent variables. Um and detection bias is concerned with. How? Um, systematic error can be made through any data collection or determination process for those specific recorded valuables. For each of those variables. So does anybody have any thoughts or questions or comments about how detection bias may apply in the OR what it means to you in the in vitro design space? There are no wrong answers. If you have a question, just ask a question. Please put your hand up if you want to go or just speak. There's not lots of us on the call, so you can just say things if you want to.

Participant 1
So just to be sure then I got the definition and so in other words, is the ability for the methods and the experiment to either detect a signal or somehow alter the signal in a way that is artificial?

Paul Whaley
Yes, it could be either of those things or more of them. So this is very much about your interpretation of it. Don't worry about the true interpretation of it. So yeah, so anything really the. Yeah. If you've got a signal. Anyway, in which the recording of that can go wrong, or it might be you have a. Independent variable and the matter of that could be incorrect as well, right? So you could be talking about incorrect matches of the dose as well as incorrect matches of the response, for example, or temperature, or really any data that's being recorded for the purposes of. Interpretation and results. Can't analysis.

Participant 1
Okay, thank you.

Paul Whaley
Getting a few nods.

Participant 2
Yeah, just checking if I get it right. What comes to mind here is is visual scoring of which is done a lot in vitro studies which. Probably is subjective by nature, and that might open also. Open door for bias. A lot of genotoxicity testing where I do a lot of counting of. Cells..

Paul Whaley
Counting themselves. Excellent. There are other ways in which visual methods or approaches. Ohh sure. Excellent.

Participant 3
I'm also thinking here about, for instance, not having calibrated equipment, not having validated equipment internally, even simple things like diabetes. If you're not calibrating them properly, implementing, for instance, good in vitro method practise in terms of equipment, you could introduce a bias, even if voluntary by bias. I'm not sure if that fits with this particular topic. But that's something that came into my mind.

Paul Whaley
If you think it fits, it fits. Don't worry about if it fits or if it doesn't fit. That comes to your mind. Say it. It's all we're trying to do here is record people's ideas, so there are no wrong answers, just if it's there, just say it.

Participant 4
In the same line, I was thinking about like the it's not. It's the calibrating of the equipment, but it could also be different. Yeah, they're they're different brands of equipment. If you have HPLC with different columns, I mean, while they're all filled with the same thing, but there could be variation on on some of these things as well. So a little bit equipment related changes could also, but that's what I was thinking as one thought.

Participant 5
Are you considered in this area in this couple? Also the you know the. Thing. And interference that you may have with some reactant in these daysdays I am dealing with the a very brown substance that is interfering with the entity in cytotoxicity. I don't know if it's in this scope or not.

Paul Whaley
Like I said, there's no right or wrong answers, so any idea that you say, even if it doesn't fit here, we'll code it as fitting somewhere else, so that is no problem at all. Thank you very much. That's question. I couldn't even tell you if that was correct, but that's not the purpose here anyway. Fortunately, and Participant 6?

Participant 6
Uh, sort of building off of the train of calibrated equipment if things aren't defined up front, or then also reported in the methods and materials you might not even have information during assessment to figure out if there are any potentials for distortion. So I don't. I don't know if again if that goes here, but it's a thing.

Paul Whaley
It is a thing. Excellent. Thank you. Yeah. What I'll do is I'll just flash up a few examples that we came across so. I think we just kind of maybe touched have a slightly but the common or or? Issues that like a number of criteria where at least related to in the tools that we abstracted criteria from, included issues relating to detection of exposure, so mismeasurement of exposure. Miss measurement of outcome. The use of appropriate comparison groups that controls and those comparisons so would be nice, maybe just to talk about that bit more detail. And then the masking of investigators, we've got outcome assessors who are potentially aware of which the exposure group is and which the comparative groups are. That may lead to them interpreting. Sadness made maybe baking subjective judgments differently, right? So maybe comparison group, since we haven't talked about those yet, this could include obviously positive and negative controls as well. Does anyone have any say anything about how? Comparison groups could be involved in systematic error in in vitro studies.

Participant 6
If they're not run concurrently with every trial, that could be problematic and they would not be useful.

Paul Whaley
Okay. So there any issues that may occur where? Like it might. Maybe there's ways in which comparison groups and exposure detection are maybe even interact with each other to some extent. So are there scenarios in which? You have a perhaps in the multi world plate or something you have a comparative group which is supposed to be receiving a certain dose. And then there's some way in which. The dice that is actually received might be different, or is mismeasured in some way. Um such that your comparators aren't quite what you think they are as a scope for that within in vitro study designs.

Participant 3
Yes, I suppose I'm. I'm not sure. So sure. Well, sorry I I just started speaking without raising the hand. But. You know. I suppose for in vitro studies binding to plastics and having proper kinetics of what the those that is actually exposed to the cells is important when it comes to comparing comparison groups. Well, if they're using the same material and the same conditions, I suppose they are still comparable even if you're not estimating that those correctly. Um but, but yeah, kinetics are quite important. Understanding what your cells are seeing.

Participant 2
Yeah, yeah. What also might happen that you have not a spillover but you have volatile substances and if if you're high concentration is next to your control in the play, that might affect the control level for example.

Participant 3
Right, I I fully agree with that. Plate design is really important in these studies and you know different plate designs may give different different results.

Paul Whaley
Interesting. So maybe that's worth pursuing them because this is not a term that's come up previously in the focus groups. Or do you want to expand a little on how plate design can affect? Natural error.

Participant 3
Yeah. Like what? What Participant 2 said, you know, evaporation is not the same in all wells of 96 or even bigger plates. So you have to consider that you also have to consider how in your plate design decrease contamination between different substances, right? So you can have designs where you try to minimise mixing of different compounds in the same plate to avoid avoid cross contamination. Also, the way you place your concentrations across different wells is also important, but I think there is information out there. About proper plate design for for a study. To minimise these effects and then you have to test in your own system. Because that also may depend on the system you're using.

Okay, thank you Participant 1.

Participant 1
I'm just thinking about him, like looking at a study and and applying this. I'm and I wonder. About reporting, if this is also a reporting question, I mean there's the interpretation that they did it correctly incorrectly, but. A few studies report this level of detail when they discuss methods so. I don't know wondering what happens in that scenario. I'm looking at a paper. And weighing how much confidence I have in it, they don't report. That on comparison groups and how it was performed in the study. So we punish it then, or do we assume make an assumption? OK.

Paul Whaley
So right now all we're doing is trying to harvest as many ideas about what we could think about when we developed the tool. We're not worried at this point about. How we wear your assess or which ideas are important or which ones are like what we're trying to do, is get a what isn't discussed necessarily in the tools that we've abstracted criteria from and plague design was not something that was front and centre in those tools. So if it's important, then we would include it potentially. And then if it's important but under reported. Than we would develop guidance for the tool for the invites in accordingly, and it may be that there is a need for if you know through invites in or some other development process. We are identifiers in which that systematically under reporting which should be addressed by development of reporting guidance, then maybe we would undertake that as well. But these are all separate issues and what we're really trying to get a handle on now is what you think is important when it comes to doing and in vitro study in such a way that you don't get error in your results. And then worry about the other things a bit later through separate processes.

Participant 1
Okay, thank you.

Paul Whaley
All right, welcome. Um, so don't also have any comments on this idea of comparison groups.
Potential for our there as it relates to. Kind of missed detecting if you like your exposures outcomes rather she's. So something else that maybe is worth discussing then is the reason that masking investigators comes up as important in some of these studies is that people are making subjective judgments about. Um, what kind of happening in some data, and if they're aware of whether or not the data they're looking at belongs to the comparison group or one of the exposure groups, then that might lead to them interpreting the data one way rather than other, which would then lead to errors in the recorded valleys, right? So I think, Participant 2, you talked a bit about counting. I mean, are there any analogies from say, we're not talking about his pathology here? But obviously histopathological like people like analysing a slide for histopath kind of purposes. There are some concerns there that not masking. Which expansion group the slide is from there and anything at all comparable in the in vitro space potentially.

Participant 2
Yeah. Yeah, what I mentioned earlier, I think. Yeah. And the genotoxicity test you you produce slides and and then they they count certain misformed cells and in some exercises you get you can easily or not easily you can you can. Code those lights so that the the actual scoring personal doesn't know what they're looking at, whether it's a treated control, what, whatever. So in order to avoid the bias, if you would know that's the positive control, probably you would count more than than you would if you assumed it was a negative control. So that that's very comparable to Histopath practises.

Participant 7
And you can ask for a second opinion or a peer review of your slidesslides. If you don't. Another expert if you want to that.

Paul Whaley
They're only how much sort of computational machine based approaches are the for in vitro designs that? Are they coming becoming mainline or is it still primarily a manual process for a lot of the stuff? That's true.

Participant 4
I think. That depends a bit on where you look at. I guess there it's increasingly becoming more automated, but you know, I think it's, I think that's the whole range still there. I would guess.

Participant 2
Yeah, it's again the the same example, but I'm, but I'm not up to date. I know from some test methods they were struggling to get as good as their trains score was, so I, but I'm not not sure where those stories are. It's again, the gene talks cosmogenic. Custom tenacity field.

Paul Whaley
Alright, so I will move it.

Participant 7
Even though you have alternated scoring, let's say from imaging or something like that, and quantification based on that, you, you and may need to set up a perfect protocol to capture the traits and the morphology in the cells or the cell cultures to use that protocol. So that's kind of an expert judgement or yeah, depends on the investigator.

Participant 3
What what I've seen from image analysis when it's not automatic. At least in guideline methods, there has always been a need to develop an Atlas. Both in Geneva talks, irritation analysis of histopathology. Without an Atlas, there was a lot of variability and bias in in the evaluation of the images. So in both cases Atlas had to be, you know, developed to indicate how to score different types of images. If you do this automatically, you're obviously decreasing the bias. Um in principle.

Paul Whaley
Yeah. OK. Thank you. So things that we found difficult to interpret so. There was a little of discussion of detection bias relates to timing of exposure. So also like a lot of varied language around this as well. So there was there was talk of you know specific time points at which a measurement is taken and given in vitro. To essay the window of exposure, then measurements in relation to that.
Ohh, the duration of the exposure measurements in relation to that and then latency parents as well. Sorry I've got someone at the door. Just be right back. Sorry about that. They had nothing to contribute to this call. Yeah. So we're talking about time points when his rental agency periods. I'm just wondering what your take would be on how timing of exposure can potentially distort. The recording of values for a variable basically.

Participant 8
Yeah, this is this is really an item that is very important in, in, in developmental toxicology, in vitro tests. We've been doing a lot of studies with stem cells, for instance, where you see that for instance, gene expression after exposure is is usually very high in the 1st, 8 to 24 hours. Where is the morphological read out in your cell system may take 10 days to appear, so you have a very early response and have a very late response. And so it it is really very important to to to get the right time point dependent on the question that you're asking. That's so that is, that is an important thing. The the other thing that that came to mind all already earlier on is. When it comes to detection of certain, you know, biological activities such as the beating heart muscle cells, that is, that's one of the prominent essays in in developmental toxicology. It is very important that you use a microscope with a heated plate underneath because if you cool down your your culture. More and more and more progressively, the the beating will stop it, simply because the temperature and and the same thing is true for you know, for incubators that are being open to every 5 minutes the the internal atmosphere will be different from from an incubator that has been closed for a long time. So what we what we what we use is not only these heated plates but also incubators that are only used for certain test system and that are only opened at really well controlled moments. In order to make sure that there we always have the same conditions, so those those things are very important for for yeah. And and and biassed unbiased measurement of endpoints.

Paul Whaley
Very good. Thank you. It's just something that did come up on the last focus group was when you got essays that last quite a long time. So you said 10 days. Are there any issues that overlap like you know towards the end of that 10 day periods where measurement issues can become a problem simply because of the age of the system and things like that?

Participant 8
Well, when it comes to the stem cell system that we use. It 10 days, it's kind of the optimum time point for. Counting beating muscle foci. So that is really been been, you know, adjusted to to the optimum. But if you, I mean it's true if you leave culture for longer periods of time in terms of gene expression, we've seen that the longer you keep your cultures, the larger the variation becomes between parallel cultures. So again, there is an optimum there too. The longer you wait, the the the bigger your effect will be, but also the variation will be will be increasing. So there is somewhere an optimum time point where you see enough of a response that is not so variable that you can't that you can still differentiate between between different exposures.

Paul Whaley
OK. Thank you very much.

Participant 7
Yeah, I can understand a little to what all it says. We said we we have a culture of which lasts for 28 days actually. And what we see is exactly the same what was described now that the gene expression changes comes very early and the more functional parts come relate let's say after three weeks. And they also know that the processes we are looking for, which is neurodevelopmental processes, they are pair of different time points during the development. Some may appear at one week, some others are two or three weeks. So depends on what time once you select your emails to get some false negatives or false positives. If you do the wrong time point selection.

Paul Whaley
OK. Thank you very much. So then noticed a couple of other things to discuss. So. I just wondering what role kind of being outside levels of detection equipment might play in terms of systematic error cause it sounds like a little bit like a type of censoring bias. And then there was this concept of marker cut off points that came up in our literature review, which we weren't quite sure what that meant at all. Whether or not that relates potentially to levels of detection issue or if it's something to do with deciding whether or not a given outcome has manifest or not, potentially so something to do with conversing continuous data. So categorical data or something like that. So either of these two, if anyone has any thoughts or comments. You very much appreciate.

Participant 8
Yeah, I mean this, this, this speaks to a discussion that we're having all over and over again about, you know, are you going to score in vitro, SS positive versus negative, whatever that means. But that would mean that you need a cut off somewhere or whether you want your read out to be in a concentration response fashion. So you so you could also look at potency of your compound and I think the latter is of course superior to the to the, to the former and there are many in vitro essays. Especially in the old days that that have defined a cut offs for positive versus negative based on a training set of a limited number of compounds and then it's such an essay is used for a totally different set of compounds. The the predictive value, whatever it means, is not as good as you might have expected, but that's just simply because of the yeah, in my mind, relatively naive way of of scoring plus versus minus. I I think you should never do that. But but look at concentration response always.

Paul Whaley
Okay. And now, thoughts or comments on this?

Participant 3
Yeah, yeah, no, just a comment to what others said. I I fully agree with that. I've seen, however, several sizes giving constraints, concentration or those response curves and then those being converted into category called decisions. Anyway based on EC50 or something like that. But you know when you introduce cutoffs, obviously. The uncertainty around the cut off is usually quite high, so you know if a lot of your results and. Our fall falling within that, that region around the cut off the uncertainty whether you really have a positive or negative result is, is. . Much higher. I've seen some guidelines where people establish a, you know, the cut off plus, minus is a certain value and you just call that uncertain or you repeat the test just to have more certainty certainty on on your final call. If you're far from the cut off, then there is less problem. You know in the categorization.

Paul Whaley
That's interesting. So that's. So that's that's just doing repeat measures. Based on Observed data that sounds like that, yeah.

Participant 3
Right. Repeat measures when you're falling. Quite close to the cut off that has been established just to have more certainty on your final call.

Paul Whaley
So Participant 6, we're just going to come back to just just to chase this particular rabbit. So sometimes draw like in one of our other domains, there's this idea of repeating measures until you've got data that you like. Make some people quite nervous about potential for arrow, cause you don't really have a stop point which you say OK we have the data now and this is how uncertain we are. Is there a potential that? When you're near a cut off point and then you decide to take more measures because of that. That that might introduce maybe unwarranted certainty because you're repeating a measure until you've got a result that you feel comfortable with.

Participant 3
No. Yeah, yeah, I fully, I I think there is a well we've done has always been limiting the number of repetitions, right. You cannot just continue continue to repeat. I mean even in validation studies at least those that I've coordinated, for instance, when you had to obtain 3 valid tests in terms of repetitions for assessing reproducibility. You have. Up to five to obtain those 3 valid tests, because sometimes you you may actually. Have a non valid test within that series, but if you permit continuous testing, the lab is actually improving as it tests more and more, so you're introducing A bias because further on labs will not constantly repeat 10 times to obtain a result, so we always limited to a let's say a number of five to obtain 3 and if you wouldn't be obtaining those three within the five opportunities you had then you would have an incomplete data set and that would be reported as such. Because that's also valuable information. But you know, I don't think repeating a certain number of accepted number of times is a problem is the problem is if you don't impose a limit and you allow you know for the lab to decide how many times they're going to test. And then of course reporting all the data, not choosing data. That's very important. Including those that were not valid, that justify the repetition.

Paul Whaley
Very good. OK, thank you for that. That was that was very helpful. So it was a slight digression. Participant 6, back to you.

Participant 6
Yeah. I just wanted to circle back to that equipment issues, additional to levels of detection. One of the things that I've noticed is we have increasingly more sophisticated tools and equipment that we can use for data gathering. And along with that comes the ability to save the metadata of the equipment that has all the parameters and the functionality so that it can be verified to be what you thought it was, but that often. Is not done or not available so. I think ohh along with saving the actual data that comes off the machine a lot of times that's put into an analysis sheet and that's what you move forward with. So having the ability to. Look back to the raw data. I think is a potential way to help against protect against bias.

Paul Whaley
Right. So I think we should get on to our next domain. So we're actually going to be from reporting bias. So Participant 3, showing some psychic abilities there. It was not the most populous of our biassed domains had 15 approximately unique criteria relating to. Potential how reporting choices can distort the results of a study. So our definition of reporting bias is a bias due to distortions in the selection of all representation of information in study results all research findings. So this is about the choices that researchers make. And either choosing the data that they report. Or how they choose to represent it so you could call it something like spin or interpretation or something. In either of the results. So these are the bits of the data that they report, or the findings of the study. So this is where more about how it's interpreted and discussed. So just off the top of your heads. Um. Any thoughts or ideas about how this applies or may apply in in vitro design study space?

Participant 2
Yeah, in, in my experience there I I run into a. A steady appetite to exclude values and and often the phrase outlier is used and then they want to come up with some way to justify their their removal of outliers. I think that is critical and things people still just exclude outliers based on.And visual inspection or so and and that might definitely introduce a bias. If they're not real outliers..

Paul Whaley
Very good. Thank you.

Participant 3
I've also seen cases of um, you know when you're transferring data and you're doing it automatically. I've seen cases where people use an input file to, you know, with the names of substances, for instance, that is used to, to then pick up automatic light and transfer it to another system where the light is analysed and if the input data has an error, even a spelling error, you may actually miss data. And I've seen that happen and introducing a bias in the results that was unintentional, but it's there. And if you don't verify it. So, you know, quality assurance, quality control in all the processes of of data acquisition transferring and and processing is quite important..

Paul Whaley
Alright, good. Thank you. So that's the. So that sounds like a a detection issue because you've gone a recorded variable that is incorrect. That then leads to loss of data from the analysis, right? That's a very good point. Thank you.

Participant 1
Kind of a question, so maybe an example. Also something like studies that report results, I'll say incompletely. For instance, a western blot, and they just give you an image of the results and then they tell you ohh, we repeated this several times. I'm just giving you an example of what, how the results come up, but you don't. By doing that you don't get an idea of variability and how that responds. May change a little bit from 1 replica to another. Would that fit in in here as well?

Paul Whaley
That does sound like. Where even well intended, it does sound like a selective representation of the actual data, because there's the authors, it sounds like all censoring if you like.

Participant 1
Yeah.

Paul Whaley
Some things they might be considered to be, you know, duplicate and boring and unnecessary, but would actually give you a sense of range, right? So that would fit it.

Participant 1
Yeah, it's maybe just aesthetics, you know, choosing the prettier image before the publication, that sort of thing, but. Not very useful. OK, just wanted to clarify, yeah.

Paul Whaley
Thank you for that. So I'm gonna just pop up a couple of examples that we found a little bit easier. So very high level. There's choice of reported exposures. Um, so this might be a missing. I guess in some way exposures might be considered outliers or something like that. There's the reported outcomes which I think speaks quite directly to the point Participant 2 already raised. This choice of reported analysis, which comes up a lot in the literature, so that's where people, you know, calculate results in multiple different ways and then only present the a subset of those calculations if you like. And then there's also as kind of, I guess maybe a type of choice of reports analysis you can have. What multiple analysis that are done and then? Sorry this so this is a result so multiple analysis that are done. And the author's choose the most. Favourable results, right? All the sub group that gives the most favourable results and then sorry, the report analysis is they have multiple analysis and they only tell you about certain ones they did rather than choosing favourable results from a given analysis. Think, who would? I don't like to comment on this. Ohh give anyone any ideas. We've seen this happening. Is it difficult to see happening? Usually reporting bias is quite difficult to see for obvious reasons. And the thing that you.

Participant 4
I guess I'm I'm I'm struggling with this. Sorry for jumping in a little bit. It's like is there's like what you're supposed to report versus what is done. . I mean, I don't know what you are. I mean, what you're looking for. Like what? What? What we've seen happening, what could be even though you shouldn't or is it about like, what is already? In the first, I guess is. Yeah, what can happen?

Paul Whaley
I think it's just anything that you are aware of or have seen or you would be concerned about that would lead to a systematic error. In the results were findings of a in vitro study and in this particular case we're talking about how the reporting practises. Of researchers. Can introduce those types of error, so there might be rare. They might be common. It doesn't really matter at this stage, it's just things are aware of and would be of concern to.

Participant 4
I guess maybe one thing I would think more looking thinking for what comes to my mind is units missing or Don confusion with units and. Yeah.

Paul Whaley
So it's unit conversion reasonably common. I'm very naive, so I think that I know my telegrams from my Oz but.

Participant 4
I would think so. I've seen a few, but I mean the the methods are. If you're looking individual endpoints maybe. Various methods in the same field have different units and that could kind of feel. I know whether that's all. Yeah. Comes more in the reporting or not? Maybe. Yeah. My dear.

Paul Whaley
It might be a detection issue, I guess because, but it doesn't really matter. It's like we capture this stuff anyway, it's fine.

Participant 2
Yeah. I just wanted to confirm that that every now and then you run into. Of figures and units that just don't make sense. And so it's it's a thing that you have. Wrong. Concentration reported by a factor of 10 or you have then the deviation reported, but they do standard error of mean or something that happens. Ever known that?

Paul Whaley
Good. So the things that we found tricky in reporting bias to kind of wrap our heads around which you may be able to help with is in particular the selective emphasis on post hoc analysis. So just to translate this, it's the idea that. You have like per protocol some analysis that are planned and then you have some kind of like a X protocol analysis that are kind of exploratory or you know where you're feeling your way through the data, but maybe not where the prespecified plan. And then there's sometimes, you know, because that feeling the way through looks more exciting or interesting or better, supports the author's ideas of what is going on. But then there's selective emphasis on those exploratory analyses over the prespecified ones. And this leads to an impression of maybe greater significance in the fight in the study than with otherwise. There is this something that is familiar to you that you seen. That you have any thoughts or comments about? So what was it called? Pick up nodding on the audio. But I did see Participant 6 nodding quite vigorously, so I don't know. Participant 6, do you want? I'm gonna put you on the spot. There are common.

Participant 6
Now, while I was just going to say that anytime that I have seen that you know, well controlled study, there's additional narrative around what that post hoc after the fact analysis is that helps to explain it so. My my. Exposure to it is always with a good. Beefy paragraph explanation on what this post hoc analysis does and why it was done, and sometimes even additional thoughts on controls around that too.

Paul Whaley
So that's kind of a circumstance where it feels less uncomfortable, I guess. Yeah, very good.

Participant 6
Right, right.

Paul Whaley
So I don't know have any thoughts or comments on this. Okay we shall move on then. Selection bias is our next domain. Kind of. It feels like it's important. It comes up a lot like 20s, you know, somewhere in the middle in terms of number of criteria. There are a given domain, but it is quite difficult to interpret, so we're probably gonna spend a bit of time on this. If you have any questions, please ask. So selection bias we have defined as a bias resulting from. Methods used to select subjects or data. Factors that influence initial study participation. Or differences between the study sample and the population of interest. So it is very multifaceted domain. And obviously because a lot of the language comes from health research and controlled trials and either humans or animals, there's language like participants where you wouldn't necessarily consider a multi well plate to be a participant per se. But it is kind of a study subject that is put into the experimental environment. So I hope you can run with the metaphor. So here the methods you select subjects or data. Obviously you've got study subjects. So that's the physical entities that are being experimented on. And then you can also be selective in data in the data that kind of you either. Admit to your analysis basically.
So it's not reporting bias, it's about excluding data much earlier in the process. Um, there are factors that influence initial study participation so classically for, say, selecting rats out of cages. Like if you just going to fish rats out with your hand, you end up fishing out systematically the most inquisitive and energetic rats. And you systematically ignore the rats. That timid and frighten. So you get some selection bias, for example, in your study, if there is an analogy to in vitro design, then I'd be really interested to hear about it. And then there's potential for differences between the study sample and the population of interest itself. So this is about a systematic difference between the things that are the physical objects that you're in your study. Versus the things that they are supposed to be representative of. So the population they were simple frog. So I mean obviously any questions or thoughts as to how this applies in in vitro context that please do? Ohh ask or suggest or anything.
And it is a complicated idea. So Participant 2.

Participant 2
Yeah, the first thought that came to my mind is during my PhD we used for my colleagues did. Use freshly drawn blood from coast students and smokers were preferred because they had to hire a response, usually for example and and I think that that can be brought into a lot of or in vitro assess where primary cells are used. Human primary cells at least, and that would probably also talk to the last point a little bit.

Paul Whaley
So that would actually be help for them, because this is one of the things that was never really crystal clear from the. Tools that we were looking at was that when they apply to. Primary cell cultures I guess. And where and when they apply to cell lines and how these considerations might be different.
So when we're talking about primary cultures then? Um, these experiments that are done with the intent of learning something about the populations that are being sampled from, or they convenience samples because we need like primary culture from somewhere or I mean.
What? How would this affect and a selection as much as say, inference or something about? The meaningfulness of the results.

Participant 2
And. If I may, I I think you material is is hard to come by. So it's it's not that you really have access to to bigger population for any type of of tissue you wanna sample. Um the the, so I think it's simply the limitation of having that material available on that that might result in biases. The thing you mentioned at the end that it's used to study properly. Population variability, that is only.
Coming up, at least in my experience over the last five years, that experiments are designed to do that. Ready to model population variability by using a planned sample from a from a broader population or even across ethnic groups or whatsoever.

Paul Whaley
Ohh so it's fast and won't come back to the couple of questions about Participant 1 you have your hand up.

Participant 1
Yeah, I have a question for Participant 2 and the example that that you would just giving about smoking, do you see that as a as a bias or? Informing the results from such a study, informing a particular population so the those type of results apply to smokers.

Participant 2
At this specific example, that information wasn't really recorded. Everybody knew or or the people in the lab knew that smokers used to be better responders. So you got in a higher item, interleukin response or something, but that was never really then reported or recorded in any systematic way.

Participant 1
Ohh okay alright.

Paul Whaley
So I guess then, Participant 2, that means there are potential systematic differences between, say same study performed by two different universities, you'd have different prevalence of smokers at the two universities, you could end up with different results in performance that that essay between two different institutions. Am I right?

Participant 2
Yeah, yeah. And smoking is just. One example of a confounding variable. That might be so many others.

Participant 6
Yeah.

Paul Whaley
OK. Ohh, Participant 6.

Participant 6
Ohh, I was just gonna say along the same lines just for primary cells. The genetic diversity of the population also not just are you smoker or not like another example could be just who's available in the population depending on what university you are and the diversity of the subjects that are. Now come coming to give samples or the source of the cells. So that that could also. Skew your results.

Participant 3
And when it comes to sell lines, genetic drift is also a problem, because if you're using the cells the same cell type. You know, but they've been started differently or different sources, or you've had it stored in your lab for a long time and you're not doing a full characterization, genotype, characterization. You may actually have genetic drift and not have actually the same cell line as other people are using for the same essay, which could also create differences and and that also, you know, you were mentioning a while ago. Long. All cultures that could also lead to some changes. Um, you know which could also introduce bias in the results in a way..

Paul Whaley
I guess thank you.

Participant 3
OK, carrier type characterization is something that is not usually done. But it's quite important.

Paul Whaley
Good. So this might become so. Up then, in some of the things that we're gonna just highlight because we've seen them. So these are things like cell line authentication, cell line contamination. The conditions of cultivation or maintenance and how these can affect population characteristics.
Something that we haven't maybe talked about is exclusion of units from analysis due to missing data. So there are situations in which. Ohh, maybe for whatever reason like a a plate hasn't produced the data that you wanted or something went wrong in how things are being measured and so you just kind of got. Um, either just sort of meaningless garble or just blanks where there should be data.
And then. The source of that ice are excluded. Is this something that can potentially happen in in vitro set up? Who would the with the fact that there has been no data, would just be recorded? Just thinking about selection of data into the analysis at this point. So you're saying, well, this date does not meaningful or there's something that was date is broken. So we're not going to include it in analysis. As this I mean, is this categorically different to exclusion of outliers or?

Participant 7
If I may comment on this, sometimes you do expect results from a study you're planning and may have an opinion that this may lead to some sort of adverse effect. But if you experience the negative findings from your study. And then you may think maybe something is wrong here and you do not then report the negative findings so you get on by us about. In negative yeah, results. I'm not sure whether that's more to the publication bias or the selection bias. But I think the the reporting, at least all negative findings are. You know can be a problem.

Paul Whaley
So I think the reason that. I think so. The one of the reasons that this is distinguished from reporting, I think at this stage is that some people who develop study assessment tools. A concerned about differentiating between when. Data goes missing because it's not introduced into the analysis at all.
Versus data going missing because it's been gone through the analysis but then hasn't been reported. So I'm just wondering if that's like an important distinction that we may want to be aware of because it would make me trigger different questions. In the assessment tool for making sure that we perhaps know the difference what's happening here, all we've covered, both like eventualities perhaps. Wait, this is not very clear. Anyway, it's possible it's just not clear.

Participant 3
I I think the the the basic principle is that all data should be reported.

Participant 7
Yes.

Participant 3
Everything should be reported, then. Reasons for exclusion should be explained. Then you might agree or not agree with that, but at least there's transparency. If you decide to exclude data. Ohh, you know because you think it's an outlier because you think you know the positive control didn't work and therefore the data according to your you know idea are not valid or for any other reason but you don't report that, then you're introducing A bias obviously.

Participant 7
Agree.

Paul Whaley
Good. OK. So the things that we found difficult so. There's some discussion of cell density cause like we're really kind of one of the things that we're not concentrating about that in this focus group specifically. But the tool is being kind of designed in the first instance for. Cell cultures, then cell density comes up as an issue in in, I guess probably more than just our culture studies, but like it wasn't clear how we should make sense of these concerns about somehow or cell density distorting the results of a study, whether or that even fits under selection bias or. Ohh how it sells. You basically can impact. I started on the potentially cause systematic error and its results. Still fix results.
I'll go with Participant 2. You just beat you out of the punch there. So we'll do Participant 2 first year or second.

Participant 2
Yeah, not. Not sure where it belongs, but I've been recently involved in a project where Celsius to have to become confluent and then they were treated, but that was a very subjective decision when they were confluent like 95% or 98 and that tremendously affected the results. So that was. Was a big thing and that that might also you had earlier like time, timing of exposure and so it's so the onset of exposure is is also very crucial that the. Um test systems are always exposed. At time .0 at at the same level of. The same state, in this case confluency of cells, yeah.

Paul Whaley
So just for the transcription, could you just define confluency as a concept for its very quickly?

Participant 2
Yeah, I I think it's it's how dense the cells are in wherever you grow them in. But others have more experience than I. Here. Yeah, it's. It's a kind of cell density measure.

Participant 3
It's how how, how, how, the extension in which you recovering the dish. Basically 100% confidence here. Basically the cells are all touching each other and you're you have covered the whole surface. And that's how you measure confluency I and I. I think Confluency has a big impact on results, but it's difficult to say which confidence you want to use because that's context base it dependent, sorry. So in some methods like Participant 2 said, you want to have. You know, near near near full conference you want to reduce maybe cell proliferation, other assays you want to to be far from confluency and you still want to have self preparation it it depends. You may also have morphological changes of the cells once they reach confluency. So you know, the important thing is that you define. The THE conditions of your essay. You justify why you want to use those conditions, and then you use always those conditions, because if you change them, you're going to introduce potentially introduce changes in the results.

Paul Whaley
Very good. Thank you.

Participant 8
Yeah, there's there's two examples that come to mind here. First is, we have some of these essays that are measuring, you know, transwell resistance in a monolayer and. That's so those are two two components or two to what is it, 22 window cultures and what you want there is first to have a confident layer of cells with with disjunctions and you simply measure the transferral resistance, which is then a a measure for a confluence. So you you have kind of a basic. A minimum resistance that you need before you say, OK, this culture is good enough to go into the test and that that is a very nice measure that's better than than just observing confluence of cells. Of course the the other point that comes up is for instance again for for embryonic stem cell differentiation the the the number of cells in the cell density is very important. If you if you go too low, you will never have this differentiation. If you go too high, you get proliferation at the expense of differentiation. Do you need a very, very clear number of cells or a clear cell density and that that can of course easily be achieved by by seeding the the appropriate numbers of cells. But behind that of course is the culture from which you take those cells. You have a basic stem cell culture, that is, that is kept and that is maintained for longer periods of time for us for a set amount of of passages. And I remember that for some of those cultures where the where the culture conditions. This work kind of kind of intrinsically variable. We usually set up to three or four different densities, and then two days later we looked at what they looked like. We took, took the best one by observation and and took that down to the next passage, again with with a number of different densities and. And. We then by experience took the best one. What what we thought was the best one by morphology to to put that into the actual test system. So there's actually 22 layers there first. That you know the basic culture from which you take the cells which need to be healthy and the 2nd is the density that you played yourselves with.
So they both can give rise to to to biases. I mean some of it is expert judgement and some of it can actually be measured, such as the transworld resistance.

Paul Whaley
Super. Thank you very much. I think trial which I don't know if you were responding to Participant 2 or if you had a separate point when you put your hand up that you wanted to make. It's like I just cheque that. Yes. Yeah. So when you put your hand up a minute ago at the same time as Participant 2, I think you we we had you respond to Participant 2 but that's one to make sure that you didn't have a. There was this OK.

Participant 3
It was the same. No, it was the same point, yeah.

Paul Whaley
Very good.

Paul Whaley
Does anybody else have any thoughts or comments as that that dance discussion is extremely helpful? We didn't have that in the last focus group, so. OK. So we still have 20 minutes, so we should get through one more domain hopefully. So this is analysis bias. So quite frequent, not often referred to as analysis bias. We had 32 approximately our unique criteria for potentially assessing distortion in results from the study due to. Ohh that was methods. So the definition for analysis bias that we're working with. Is a bias related to the analytic process applied to the data. So in this case we have the data from our experiment and then we analyse it in order to produce results. Those analyses are analytic process. It's obviously lots of ways in which. The analytic process can result in distortions to the findings and results of a study. I'm just wondering off the top of your head. Think of any examples for in vitro studies how that's going to.

Participant 4
Isn't that a bit related to the detection or?

Paul Whaley
So detection produces the data. And then analysis kind of handles the data, right?

Participant 4
That's OK. So I'm done. OK, that's really looking can't, OK.

Paul Whaley
Ohh sure I'll.

Participant 3
Well. I think. For instance, with OMIC data. There is a high potential for bias. Although you don't really know what's correct and what's wrong because of lack of standardisation, but you know.
Um, you may actually normalise the 8IN multiple ways you may define. Cut offs differently when you consider that the gene has been overextended, induced or not. And that that's very much dependent on the on the person analysing the data. And we we we are, we see all different types of of decisions and in the analysis of the similar types of data and that that obviously creates A bias. But it's difficult to know which one is correct and which one is incorrect. I I think that there is a real. You know, appetite from the OECD and they they've been working on this on standardising at the reporting and analysis of of on mixed data exactly because of this. And and of course it also applies to other types of individual data, but especially in I content data, it eventually could become a problem. And then whether you for instance also in olmix data, whether you do pathway analysis or you don't do pathway analysis, you can come to different conclusions depending on how you look at the data and you can see that many times researchers tend to use what fits the conclusions they want to have. So. That obviously, you know creates also. Scepticism sometimes in the way you know, on the conclusions of certain studies.

Paul Whaley
Very good. Thank you. 770 thoughts. Comments they want to add to the trial here. So I can also take us through. Some of the examples of criteria. That have been mentioned in love included tools from the literature review include the masking of the analysis, so you don't tell the investigators who are doing the analysis which groups the data comes from. There's the processes of correcting for or imputing missing data, which I think are come up as quite has been quite important that anyone have any comments on? Processes were correct or inputting data and then the vitro context.
I could foresee being quite complex but. I'm getting some nodding.

Participant 3
Talking about like interpolation or extrapolation to for data points you don't have or.

Paul Whaley
Potentially, yeah.

Participant 3
What exactly are you?

Paul Whaley
This is very open, so this just said all this says is correcting for imputing, so um, I'm kind of in your hands. So you tell me what that means to you.

Participant 4
And if reading across from one to the other of filling date, the gaps where you have them using whatever method to fill the. The missing data or not or leave them open, so I guess there's a big variation.

Participant 3
And why would that introduce bias if it's transparently reported and you know? I. Not sure exactly where the link to bias is in this point.

Paul Whaley
That's what I'm.

Participant 4
Maybe the missing of reporting? No, I just said maybe the missing and reporting exactly what you said draw, but I don't know that people just. Cracked in May, missing data or impute missing data, but I don't know. Ohh. That's a different story.

Participant 3
I I've seen people correcting or imputing missing data, but through fraud, inventing data that didn't exist, that obviously is a bias but that, but that's that's fraud, right? So I'm I'm not sure. Exactly what this is. If this is, you know, a process that is acceptable. You know, doing something that is acceptable, but depending on how you do it, you introduce a bias or if it's the the process itself that is not acceptable. And but it's not reported and you're doing it and and through that you're introducing biases in the results. But maybe Participant 6 has an idea.

Participant 6
Well, yeah, I was just going to suggest that maybe if it's correcting for or doing something and it's not uniformly applied if it's just selectively. Ohh well, this one we'll we'll correct. And you know if it's not transparent or universally applied across all of the data, then it could introduce bias..

Paul Whaley
Yep. When it's a tricky one, I think some of it comes from. I'm not sure what comes from, to be honest. I think some of it may come from health research where, you know, like like say, a null value for a participant can't be handled by the statistical model. So you end up like you know, you impute 0 or one instead. So you don't have a gap in the data you like you have, you have to have a value, right, so.

Participant 6
Ohh sorry I I have another example um, so maybe if there's an upper limit of where the data are reported and it's an instance where it's, you know greater than 1000, but then all of those data report are reported at 1000, it could throw off. Prediction models and further analysis.

Paul Whaley
Yeah. So that's what I say. Fraudulent imputation. That's just like that's just that's a Max value that's been imputed. Get okay. Thank you for that. So then there's, I think possibly might be kind of a slightly related thing, might not be, is the reduction normalizations of standardisation or kind of removal of noise from data? Um, I think because in vitro started doesn't quite data heavy I guess. You can have. And in the end its normalise the data sometimes and things like that. Can you think of any ways in which these processes can introduce systematic error or distort the results of the vitro study? And getting some nods.

Participant 2
Yeah. Normalisation definitely can introduce bias, especially if you you, you transfer transform the data in fancy ways to. To yeah, to try make them look good so that there will be non linear transformations that. That may happen not too often. It's more transformation data transformation issue than a normalisation issue..

Paul Whaley
Okay. And it.

Participant 2
Yeah, and and to to the normalisation. Yeah, your if you apply statistics that assume normal distribution of data. If if that is that assumption is, let me say. Clearly violated that might also introduce biases, but that that's then related to the statistical analysis.

Paul Whaley
Okay. Sort about data reduction. So this is again I'm facilitating so I don't have any understanding of this. What sort of data reduction process is a used in in vitro research and why might those be of concern such that criteria in our assessment tools? In relation to this.

Participant 3
Well, if if you have for instance multiple data points and then you just apply a an average or a median and you just reported that and you don't actually report. The underlying values. Um, you may be introducing. Well, I'm not sure if it's a biassed, but it at least. You may be selling a picture that is not apps. Yeah, the complete truth, let's say because you may have a level of uncertainty around that value that is not captured when you reduce dieta to you know. I mean or a an average or whatever, or a median or something like that.

Paul Whaley
OK. And that's what probably data reduction means in this context and it's it's just the simplification to means or medium parties and things like that?

Participant 3
Well, that's the impression.

Paul Whaley
That wasn't a question I've been correct, this is just like speculating as to what the tools mean so.

Participant 3
That's the interpretation I gave it, not necessarily what it means, I suppose.

Paul Whaley
Yeah. No, no, of course. Yeah. No, we just trying to get from people there interpretations. There's no sense of correct or incorrect it Participant 1.

Participant 1
Yeah, I was just wondering about the example they just gave, but also could also that be interpreted as a reporting bias? You know if if the researcher chose to report the median as opposed to all of the the data? OK.

Paul Whaley
I think it potentially could be. I think it's an analysis by us here because we're talking about the analytic process, not the reporting of the analytic process, right. So yeah, these distinctions are they fairly arbitrary, and they're only made in order for us to structure the focus groups discussions as much as you're not really intended to represent the truth of what's going on with bias and research at this stage. So.

Participant 2
Yeah, just thought I I think data reduction for me is that you.

Participant 2
Simplify the data by reducing dimensions or or variables in that and. If you do that in a in a in a way that it produces bias that that would probably be the case and and again thinking here about primary cells. So if you pool data across. Several donors which? Then then you would probably exclude the the donor variable, which might be justified or or might not. And if you if if not, that could introduce a bias in the data analysis. So that's the pool pooling of data topic.

Paul Whaley
Good. Excellent. So we have 5 minutes left, so I'm going to just take us through the analysis bias criteria that we didn't find particularly well value, even less self-explanatory maybe. So something that comes up is absence from analysis of predictors of missing data. Would this be meaningful to anybody? It's the community. They obviously meaningful some. Curled lips and shaking heads. What about the analyses relating to controlling for baseline differences between? Your I guess you'll you'll study populations being understood as your. In vitro cultures or something like that. Is that something that ever happens? So you you you know, you've got baseline differences between your groups for whatever reason, and then you control for those. I'm just wondering if there. We we do have a confounding covariance domain coming up tomorrow, which it may be better to discuss this in, but. Ohh that situations where you would expect baseline differences between. Maybe this applies more to primary cell cultures, but you got baseline differences between your cultures and then you have to adjust for that in order to make proper sense of the data. Participant 1 is nodding. So maybe shaving on the spot.

Participant 1
Yeah, I was gonna say that. My based on my experience from my post doc, that probably applies some more when you're when working with primary cell lines and that you get more that variability, that baseline viability is between one individual and another. Um, maybe not so much. Would sell with a cell line that is well established.

Participant 3
I I've seen it also as important even in cell lines. And I I come again to omix diet, for instance, including I've seen several methods that have included for instance housekeeping genes. To establish that by by his line and depending on on the expression of those housekeeping genes, you establish a cut off for induction that may be different from experiment to experiment, but it's justifiable based on that baseline expression of the housekeeping genes, which may differ from time to time. It's slightly different, so in order to reach a conclusion for your set of you know gene set, you normalise that to housekeeping. Means to establish that that baseline, so and I think that's that's quite important otherwise you're going to have differences that are just steady state differences. Not, you know, exposure related differences.

Paul Whaley
Okay, thank you. So then the final thing is the control for time varying factors through the. Experiments. So I think this this probably derives from. Again, human health is may even be observational, but may have application to in vitro context because you could have. In a cell culture study. Something about. The way the study is set up that there are factors that vary across time.
That then needs to be controlled or adjusted for. With this, could this potentially be the case and in vitro study? Think I'm seeing some tentative nods, but I'm just wondering if anyone has any specific examples that might help us as we try to develop this tool. Where are you going?

Participant 3
Well. Well, just to give you an example of a method I've been working with, for instance on.
Violation of a metal under a certain pH. And you can have actually a changing the pH throughout the time of the exposure and so you have to control for time variance because if the pitch changes, that's introducing a bias in the result because you want the exposure to be at that pitch for a certain amount of time. So you have to control for time variance of specific conditions of the assay and if they're not maintained, you either find the strategy to maintain them or you just say that it's not testable. For instance. But yeah, you, you you have to. To know what are the critical aspects of your essay, and you have to make sure that those critical aspects are not changing, or if they are changing, you have that under control and you know how that influences the results..

Paul Whaley
OK. That's really helpful. Thank you. So we've come to the end of the year and a half. I certainly hugely appreciate discussion, lots of energy, lots of ideas, like an awful lot for us to process and think about, which is exactly what we wanted. Lots and lots of ideas that haven't come up in the included tools. Lots of elucidation of what is in those tools, so this has been really, really, really useful to us. And I very much look forward to seeing you all again next week. I think it's at the same time going. Can you just confirm it's the same time?

Participant 3
Paul, can I just make a comment a quick comment I I I thought this was extremely useful at least for me also to understand, understand better the scope of the of the work you know, cause I've been always a bit puzzled with the words internal validity, external validity and and I think what sets this aside at least for me at the moment is that we're looking at validity of data. It's a framework to assess the validity of data, not the validity of methods. And the two things are, you know very they go hand in hand and they are complementary, but they're not the same. And and and this is extremely useful in the use of non standard data in in safety assessment you know and especially because you're using data from non methods that have not gone a process of validation. But you are able to assess how valid the data are and how confident you are on the data to to to inform a certain decision. So I think that's for me. It was really important and I only actually only appreciated today that the real difference and understanding what you're doing was important for me. So thanks.

Paul Whaley
So I think. You make an interesting quote. This is something that I haven't really been.
Fully appreciated. I think in terms of us trying to communicate what we're doing our work here because coming to somebody at this from a systematic review background like it's always about validity of results and nothing at all really to do with the validity of methods and we wouldn't consider I think in our space methods per se to be valid and it's always been one of the slightly puzzling things for us is the, some, some methods are elevated for being validated in some way.
And you have the clearest criteria which talk about the use of the validated method or OCD, kind of GLP type thing. And we're like what? What is happening here that people are more concerned about method than results? So maybe there's like a a linkage here where two separate worlds are kind of coming together suddenly through doing this work. So if that's the case, that would be great in the way that was already be a success. So thank you for making that. That's extremely helpful comment and we will certainly about that in mind.

### Focus group 2, meeting 2

Paul Whaley
So we're going to start this week with all this focus group with attrition bias. So it's not a hugely covered domain, only 16 criteria roughly from the tools that we've abstracted before. I made these slides. We define attrition bias as a biassed user absence of expected participation or data collection after selection for study inclusion. So what is happening here is that. We have a bias that is introduced because. After an experimental unit, if you like, has entered the study. So in the controlled trial that would be a person in an in vitro environment that would be something like maybe a a. Multi World Place or something like that for whatever reason. That participant either drops out so the whole plate kind of just disappears or is removed or something from the study, or any data where expecting to see collected from that study unit is also perhaps not collected. So we call attrition bias because it's like after the study's commenced, we're kind of seeing data or experimental units. Ohh start to disappear from the study right? Which should be common in RCT because you might find you know common reason for attrition. Particularly saying late stage cancer trials might be death of participant. We want to account for all of this for what we're interested in. Here is how we can have attrition in in vitro studies. So I don't know if anybody wants to. I'll have any questions or has any comments about what that means for them in the context of in vitro research. Perhaps. Anyways, in which this issue can perhaps manifest in a study.

Participant 7
Illness. I'm not sure if this is a struggle, a little bit, the applicability for the in vitro on this one could be something. Does you have a contamination? Let's say from bacteria or or from other unexpected happenings? Or is it more? That's all the data has by some reason or the testing has some reason not.
You've gone through.

Paul Whaley
So it could be that if there's contamination of a place I I could foresee that that played might then be excluded by the researchers after the study has started. Right. So you've got a stack of plates there included. There may be some of those plates you have, like a myth. Study quality control cheque or something, and then you exclude some of them. That sounds like it could be attrition, you know.

Participant 7
And if that's the case, that happens sometimes that you notice that there's some contamination or certain parts of a plate or similar, so that you need to exclude that. Alternatively, you exclude their whole plate. But then you need the some kind of proof that it's um. Uh contamination compared to a normal control.

Paul Whaley
Okay. So what I'll do is I'll pop up some examples then so. Common things that we've seen raised in the. Tools that we've abstracted criteria from include exclusion of samples from the analysis, so you've got the data from, say, the plate. And then you don't include that data analysis for some reason. So that's attrition of data. Um, sometimes there's issues around they're being incomplete. Data on some aspects of what's going on in the experimental units. There might be missing information about the dose or exposure there might be missing information about some outcome measures that were being made. There might be just missing data periods, there might be missing data on confounders if that's relevant to the study design, because I think we're talking about primary samples a bit last week, so that. That could be relevant though. And then there's also just straight up lots of samples which were just talking a little bit about just now. So in the specific cheques, one of the things that had come up that we weren't sure how to classify or quite figure out where it fits in with our understanding of distorted study results is the cytotoxicity of the test compound. Um and how this might be interpreted as in some way causing attrition, so we thought. But what would as we understood it or thought we understood it, but not so sure about from the included tools is that if you've got your test talk on, that is slightly toxic, that can cause obviously cells in your study to die and that could result in attrition. But we weren't quite sure. How that works or how to interpret that?

Participant 7
Ohh, most often you do on on. Ohh sorry to talks is test before you do the the real mechanistic, more expensive time consuming studies so you do like concentration response at least from our side. What I think is more common as a kind of problem is that you you experience lack of results. Without seeing cytotoxicity, so then you do not really know whether you have tested at the right concentration range. Sorry to talk cities. From what I have experienced, quite easy to to find the lack of effects or cytotoxicity could be a more problematic than you may need to repeat the experiment to make sure that you have covered high enough concentrations in your study compared to the real life exposure in let's say human situation.

Paul Whaley
Hmmm. OK, so there's a. Interesting. OK. Just participant 4, do you have any comments on this? Thoughts.

Participant 4
No, I guess they were just all covered. I mean decided. Yeah, that's what what? What was just said? What Participant 7 said. So what decided toxicity? Not no, no, no, that covered it.

Paul Whaley
OK, so that case, what we'll do is we'll move on to the next domain. So this is kind of an interesting one that came up so. Choice of question by us is something that's in the bias classification system that we're using, but we had no. Assessment tool that we had were evaluating, so none of the 72 assessment tools that we looked at actually had a bias criterion that fitted on the choice of question. So we have an empty domain. So when we want to do is talk about this to see if there are any areas in which in vitro studies could be vulnerable to choice of question bias. So we can start populating this stuff from expert opinion rather than from the literature that we've been evaluating. So choice of question bias is defined as a bias and research design in which the research question that the studies designed to answer is inappropriate for the context. So this would be a distortion in perhaps the interpretation of data rather than the data itself. Uh, whereby the? The Yeah, the the the findings are distorted because. The wrong question, I guess, or inappropriate question has been asked. So what would this mean to you and and in vitro research context? They might start with Participant 4. I was going to try to start with all the time because I can see face and I can't see patra's face, so I apologise for that. But, do you have any thoughts on this?

Participant 4
Sorry, I'm doing multitasking here, just got interrupted on cause I'm in the office today and not at home so it's a bit more complicated today to do the focus group. A bias in the reason, so I kind of didn't listen for the last two minutes. Sorry, I'm back now. Ohh yeah, yeah yeah, no, no.

Paul Whaley
It's okay, so choice a question by asking. Yeah, you gotta.

Participant 4
No, no, I got it. Yeah, I'm just thinking.

Paul Whaley
There may be out whilst participant 4 is thinking.

Participant 7
I'm also thinking quite hard here to come up with something. Obviously you do have some sort of hypothesis before you start to study or supposed to have that. And. Well, again, if you.
Comes up with his own answers in your study, which is really not expected on. The lack of that or.
Well. You. Hmm.

Paul Whaley
Wondering if it might be a master of say fit between the cause, like in systematically review we talk about pico questions, right, your population exposure compared to an outcome. That Picot framework also works for primary studies. You just describing the population explosion crash on the outcome that are used in the study right, rather than in the objective of the systematic review.

Participant 7
Right.

Paul Whaley
Circumstances in which may be the, you know is this is this. Could this be about an inappropriate population or exposure that relates to the research question? And then the how the results and then interpreted?

Participant 7
What we have experienced actually is that. And there's some misinterpretation. Or. Am I misunderstanding of the inclusion of studies? For let's say, brain development, because you need a system which really covers. You know from a type of stem cell or a star to development into something in the other end which is a complete network or, you know, functional cell system or functional brain. And sometimes the studies. That claim to have a development of study they have not covered the whole development, the period or just part of it. And not sure whether that's. Within this, because then you if you should include that in a systematic review, you should. You should expect it or you should not include it at all, but I have seen instead it at studies that include such as. I'm not sure whether that's because they do not really understand it or. If they if it's just fit with your own hypothesis. But do you know from my own?
Standpoint to you certainly have a defined system which is where you have some questions where and you do a study to answer that. So it's not applicable really to my own studies sort of. To do things you compared to other studies, there may be chances.

Paul Whaley
So could the. So I guess if we're talking about cause that there is this idea of eligibility, it speaks a little bit to external policy, I guess. So you've got to study design which isn't appropriate for answering the question.

Participant 7
Yeah.

Paul Whaley
But you you could foresee a situation where somebody lives, say, we're talking about developmental neurotoxicity would do a study and maybe it's. Not quite. Maybe it's not an appropriate design, but they conclude that maybe there's either evidence of, you know, developmental toxicity or not, or the other from this study that they've done and the study's not appropriate for drawing that conclusion. Then within the confines of that study, just on its own, does that feel like a distortion in? Or bias in interpretation because the. The data itself makes sense, but the findings. Ohh, the proper interpretation of the data because they haven't got the question lined up properly with the methods in some way.

Participant 4
I guess it does absolutely, but to always point, I mean, I don't know. Isn't that something you kind of you start off with a certain design, I guess I'm kind of struggling off a. Practical um and that to me the whole thing is totally, totally off. So it's I guess that's what I'm struggling with because if you start off of the wrong. Question already then anything following below all the things we talked before was more about the design of the study and the. These things, this is you start off with the wrong question, then anything is. Yeah. Any you have bias in that is. Yeah, in the out in the dis. In the yeah. If you look at the results then you kind of interpreting them in a totally wrong way but. That to me is kind of a level higher than anything we talked so far. Maybe. I don't know if that makes sense and of what. What advice? I mean, anything we had before was about like design inside of toxicity or things. Somethings go wrong within the study, but if we have here. Already starting off with the wrong one to me. That's like tear higher tier and then everything comes below. I don't know if that makes sense.

Participant 7
Or you can ask quite, you know, interesting or decent question without having the test mode to cover it.

Participant 4
Yeah.

Participant 7
So to that you used the wrong species to do risk assessment for human situation for instance.

Participant 4
That's true, yeah.

Paul Whaley
Good. Thank you. Yes. So we don't actually know how best to interpret this. We just know that it's a bias issue that's come up that we didn't see covered in the existing tools and we didn't know that because it doesn't really apply in this situation or if it's kind of, yeah, as you say, another level up or if it's just that it was kind of the new thing that hadn't really been considered in the tools that we're looking at. So this is just very helpful discussion to have and really kind of helps us get a sense of. How much or if, if at all, we need to develop this idea, if we can just push it to one side, say thank you. Um, next one to come up is obviously a controversial one, but it comes up in lots of tools, so we've got conflicted interests bias. We had two criteria for this only, but quite a lot of tools mentioned the same to criteria. So what we're hoping to do here is maybe just to develop it a more nuanced understanding of what the issues might be. The we're currently able to from the literature. So conflicted interests bias. Is defined as a bias in which decision makers influencing research design, conduct analysis and reporting have goals and motivations that conflict with scientific research objectives. So what this means is that when you've got. A decision maker in the research process, so decision maker is someone who has to either interpret information or decide how something is going to be done or when to stop or whatever when they have split loyalties. If you like. So they're being pulled in One Direction by the goals of the research but pulled in another direction by other interests they have. Then that could potentially distort the results of the study. So you could imagine that maybe. Wanting positive results because it makes you famous and the research exciting could pull a researcher in One Direction and the actual study is going in another direction of like negative results and you could see potentially that ending up distorting the study. I just want to get a sense of how you feel this might be in play and maybe things that you could see happening that might identify this as being an issue or something like this. So maybe if you want to go first in the whole turn back to Participant 4.

Participant 7
Well, it is going going from the neutral to the animal studies that I can think of quite a lot of examples from the for instance from the regulatory studies. You do see that all quite often that they designs in the studies with two low concentrations.concentrations. So because the industry do lobbyinglobbing and search and they do not want to have any financing studies because then it will. And will not be good for the chemical or the pharmaceutical compound. And then you will perhaps in the posterior with no adverse findings and then you have the discussion afterwards whether you went up to a concentration which sort of those for an animal which was high enough to catch the true exposure in human or at the level you're supposed to test according to guidelines. I guess that also applies to in vitro studies, which is within the regulatory world. From our own model or side, I would guess that. It's quite a lot more popular to publish in positive findings. You know, that's something exciting, something new discovery and search. While the more if you have a study which you know it's well designed and everything is OK and then you do not find anything at all. Then this could also be quite hard to get it published in a good in general. And then maybe you put it in yourself and start a new study where you find something. Into so-called interesting. I'm very favour in favour of publishing everything you find, even though there are negative, of course. Although I see it will be a. By us in whether it's on conflict of interest, I'm not sure. Actually it's more like. Reporting by us, perhaps.

Paul Whaley
What that's so. Do you have anything you want to add before I say anything?

Participant 4
No, because that to me is like it's similar to what we had before. I mean, I don't think, I mean there's no other example or anything. You just don't want to have that, but there's no. Right now.

Paul Whaley
Yes, I was. I think there's something interesting, they said there where, you know, we've got potentially studies being done at too low a doseAnd it sounds like it might also be another one of these kind of higher level kind of contextual issues. Rather than necessarily but bias itself, just just for us to explore the idea.

Participant 7
Yeah.

Paul Whaley
Yeah. So the only two actual criteria that have come up, sources of funding and conflicts of interest, I think you were alluding to obviously with the conflict of interest where you're looking for potentially positive results because the more interesting, more publishable, that doesn't sound like a financial conflict of interest. That sounds like it might be a non financial other type of interest that's driving so.
Probably we need to have a think about how to. Wait, if we want to think about the non financial conflicts of interest. When it comes to understanding how this type of bias might be in play. So you want to specific cheque on how you feel about understanding? Conflict of interest, not necessarily as something that is in and of itself about us, but how it's a mismanagement of interests potentially. That could be what introduce. Distortions into the results and interpretation of a study. So that would be where you've got somebody with a with split oil ties if you like, in a decision making role where they maybe shouldn't be in a decision making role, but perhaps an advisory role or something instead. And maybe that's what leads to. The. Distortion is being introduced. Maybe that mismanagement is a too low because these are quite low to terms of so conflict of interest is very loaded. Mismanagement is very loaded. If you think if I ask you about this idea of, you know, putting the research back on the shelf because he didn't get the positive results. Then it's not that that's a mismanagement issue, it's more the. If you have a policy of publishing everything, then you have a. Policy for managing that particular decision in a way that would take out the split loyalties and the person who's deciding to put it on a shelf, or deciding to publish it like the decision to publish, doesn't rest with that person. The decision to publish is a structural thing, so you have a managerial approach to preventing that split interest or split loyalty if you'd like from them, resulting in this latest report or. Um in some way.

Participant 7
I have been a few years in the Pharmaceutical industry and there I experienced that we were told by economists to to get rid of some studies with. We think we've found some quite interesting results on scientifically, but they did think that it was not interest for the you know, the economy in later in the pipeline. So they they just picked a selection of this you know this. History search to report that was for internal reporting sort of in written reports. That was kind of selection by others than the scientists. And I could also think ohh well in the projects where we we are involved in now we we want to develop new approach methodologies to you know figure out or study regulatory questions or answer regulatory questions. So if you find some. Date that which is not really going in that direction. We are in which told my the management that you should, you know. Report the research which is applicable to that only. This could also be understandable, but it's also perhaps a kind of conflict. In that sense. It could be a danger or not. Publishing or reporting all interesting data. I'm just trying to invent some questions, Sir.

Paul Whaley
No. It's what you have for me. Thank you. you have and he wanted to add.

Participant 4
No, sorry, not very helpful today here, but.

Paul Whaley
That's fine. Don't worry about it. These aren't necessarily easy things to discuss either. There's only two people here, so it is a bit trickier. That's fine. So we also have another empty domain, so this will be fun for us. Then we get more full domains coming up. But so something else that came in as predictive model research bias, we didn't have this domain in our set at all, but the last previous focus group. So Group One that we did week before last. Highlighted this as as a thing that could be happening. So then we want to discuss this with the other focus groups as well. So predictive model research bias is a bias specific to the design, conduct analysis, or reporting of research about predictive modelling. I think this came up for focus Group One. Because. That's an analogy between quite a few in vitro. To study designs and kind of like diagnostic or prognostic research. So using the in vitro study to predict whether or not he will see an apps outcome in a different. Ohh Organism or like a whole Organism or something like that. Um, but we don't really know anything about what predictive model research bias is. Cause it's seen no criteria in the literature, so it would be very much appreciate your thoughts and a discussion as to how. What issues would be specific to predictive modelling? Perhaps when you get sort of distortions and things in the studies, just so we can start understanding this space.

Participant 4
Are these are these things like um, yeah, not having not a full coverage for example of of chemical domains or not. Been having limited coverage of you know how you do your predictive modelling design and then applying it to to other chemistry or. OK.

Paul Whaley
It certainly could be. Would you like to expand on why you suggested that? Just.

Participant 4
And if you if you develop some type of predictive model. And. You're using only a limited set of training data, but then you're kind of using this to predict. Different type of chemistry or yeah. Something so done you'll have some type of. Yeah, a bias because you're kind of developing the model on a on a limited set chemical universe, a small one, and you're needing to expand that to something else. But then you're distorting your result or the. Yeah, you're biassing your. Or if you have, if you have enough in a training set for predictive model, you have. Maybe. Ffs like. Not an even distribution. If you want to do a model for a prediction of a yes, no answer, you have 90% of yes in your training set and 10% of no, and then you're trying to make a model. I mean some some uneven distribution of data, maybe something like this. I don't know whether that's covering it to be here, but.

Paul Whaley
It is an excellent suggestions appreciated. So you're asking. I couldn't tell you what the intent is. We just looking to get people's ideas of what it could be so we can then analyse myself incredibly helpful those suggestions offer.

Participant 7
Well, if you want to derive a pointed pointed departure or on exposure based on initial studies you didn't want, would like to use a proper PK modelling, right? And you want to to find the the Intracellar concentration of your test component instead of the normal concentration, which is just what you add to the cell culture. And I think often. In proper pbpk models are used because it's almost impossible to to develop or define or define a a perfect model, let's say for a foetal brain because you can obviously not find 200 foetal brains to measure compound, so you need some modelling to simulate that. And sometimes you use the blood level from the model or. In the best case, the foetus to verify what you're finding and. Ohh I often are we using feet or they I'm not an expert on this myself at all, but my impression is that. Better models are developed novel for exposure, like using the foetal model instead of the all those model to arrive exposure or the point of departure for the unborn child, for instance. And so well again, you need to find the the most perfect mode if you want to do export recalculations.

Paul Whaley
That sounds very helpful. Like excellent. This is good so.

Participant 3
Full can I can I add something here?

Paul Whaley
yes hi.

Participant 3
Hi I was driving I just got home so I maybe now I can try to participate a bit now I'm on this particular point. Um, I think maybe one of the things that could be mentioned is related to what Participant 4 said regarding training sets as we see for instance, more complex models coming in based on genomics data where the feature is being used for model development are many like biomarker composed of several hundreds of genes. When you actually use a small training set, you could actually introduce bias because the number of features that you measure is much larger than the name number of of training data. And therefore you can almost fit to whatever you have, because you have, you know, many features being measured. So that that could could introduce bias, another thing is. For instance. Model developers may sometimes use a number of features that is higher than what is actually necessary. Because that's also making their model more expensive. If you have to sell, for instance, could be a reason or another reason could be to mask really how the model is working. So you put nonsense things there that only don't really make. Um, you know, are not really having white to the final prediction, but in the end. The more features you have the hardest ******* to to understand and recapping recapitulate that model somewhere else. Um, which could also introduce somehow a bias. So I I could think of these two points from personal experience. I don't know if that's helpful.

Paul Whaley
That's very helpful. Thank you. Everything is helpful. They're like there are no wrong suggestions here because we're trying to explore a space we simply don't really know very much about. So any thoughts and suggestions? A data and data is what we need. That's.

Participant 3
Yeah. And on on the on the question on the, the one on the question, if I may add something, I I, I I thought that was very much linked to systematic reviewing. I'm not so sure if it's applicable to them by studies and Nam based data. Uh, I mean, if you. Think of a study and you design the study to answer that question. Of course that that study design may be question question by stakeholders and other people. Um, but to judge whether there is a bias introduced in the design to answer the original question, that's going to be hard to judge, right? Because it's it's actually not a straight forward thing today when you are using bespoke methods to answer other questions, how do you judge whether the the design is is biassed, or whether it's just a question of some people not accepting it?

Paul Whaley
It definitely has a strong flavour of this idea of being systematic review specific. But I can speak from.

Participant 3
I think so, I think so.

Paul Whaley
Yeah, but I can speak from personal experience in doing research as a consultant, whether client has very carefully constrained. The the the the question was very carefully constrained the scope of the researcher. The question that is asked. To the point, whereas the. Consulting researchers, we didn't feel like we were doing research which was providing an unbiased answer to the issue that was actually of interest. interest. So I think it's one of these things that's it has a strong feel of accidental nudity and the systematic review context. But I think there's also. It has been raised because it does sometimes feel like it's internal to a particular study as well, so we're not totally sure at the moment. I don't know if you have any reaction to that journal.

Participant 3
Yeah, possibly. I mean, I haven't really come across something like that, but I I think it's feasible, yeah. Sure.

Paul Whaley
Okay. We shall move on to have many domains to get through, so confounding kovarik bias today. So we had 24 criteria abstracted from our. Tools that relates to this. Obviously compounding is complicated. It's a long definition, so there with me. So we have it defined as a situation in which the effect or association between an exposure or outcome is distorted by another variable. The confounding covariate bias to occur the distorting variable must be associated with the exposure and the outcome not in the causal pathway between exposure and outcome, and unequally distributed between the groups being compared. Sorry, there's a typo in that first sentence. It should say between an exposure and an outcome. So on the previous focus group. The. Thought was that it's probably more relevant A consideration for primary cell cultures. So you do you taking of samples from people or from animals or fever and? Using them as kind of like a. Surrogates for the original Organism. But I'm just wondering if how you feel about that or you have any reflections on how confounding can be an issue in an in vitro study design.

Participant 7
For extremely profiling compounds, you may have some challenges without their earnings to plastic sometimes.. So it depends on the place you're seeing. You may. And perhaps get experience differences between experiments due to that and perhaps the protein binding, and not sure whether that's within the coastal path between exposure and I'll come or not, but it's certainly a difference between whether you're using. You know, called serum, which is not supposed to use when you in developing norms, but for the more animal derived primary cell cultures you may still use that at some labs. So there are your component bind to protein and which and then will not lead to an effect compared to nowhere. You do not have then this protein serum.

Participant 4
About volatility of samples. So I mean exposure, if you're we wanna expose certain cells or something, but you're very volatile compounds.

Paul Whaley
Should you have any thoughts as well?

Participant 3
Well, binding to plastic for instance. You know. People quite often estimate wrongly the the exposure. Those because it's binding to the materials.

Paul Whaley
So the things that came up are under the tools that we were abstracting criteria from. Related primarily to. Issues relating to random allocation of study units to exposure groups. And also to baseline differences between exposure groups. So I think the idea being that. If you have systematic differences between. Your populations then that introduces the potential for either measured or even unmeasured confounders to be in play. Is he not gonna just? You haven't got an equal distribution of population characteristics across your exposure groups, right? So could this potentially happen in the in vitro setup in some way? We're wondering if there might be examples of where. Ohh if there could be something where maybe like the the the test compound affects how the cells grow in like a structure or something so they're less dense than the less exposed cells. So that could be a confounder. That you could have a systematic difference between. Ohh I just some other way causes major differences between the more baseline differences between the cell populations and your in vitro study or just just anything like that that just brings to mind. But look there about say something quite sure, yeah.

Participant 7
Well, these are difficult one, but if if you want to design A ohmic study, you for instance there in a sequencing you you need to and then you you could. And do a selection of samples by random, you know selection and by our tool or draw from your hat or whatever it is. Sometimes you may perhaps. Do not include the right control groups in your sample, not for one run compared to in the second and third one. So well. A little unsure whether that comes under here, but do you if you have a sovereign control, you have a cell control and you have a, let's say 5 different concentrations, you should sort of include maybe all those in one run. From the same components versus if you want to test different components. So you need a proper design to, you know, select the the comparison groups and also the the different treatments. I have actually experienced that the the belongs here not. I'm not sure.

Paul Whaley
So I think maybe something that came up. I tried to remember. So there's things like. You know, I've repaired of time if the. Cell culture obviously ages over a time period, so maybe. Like, I think there's been some discussion of this. You've got different companies of different doses and you do the dose, you know, day one, you do this dose and then day seven you start the second dose and then day 14, you start the third level of dose studies that you end up with a difference between the. Cell culture on the you know then that third week, for example, As for the first week and if that might not introduce some. Like at the you know methods that could or even should be used for. Randomising in such a way that you don't see these effects happening because you kind of preventing these systematic differences between, you know, as the population like. You know grows and evolves or ages or you know something about the. You know the exposure levels, so you've got high concentrations and more volatile substances that. Those off gas or whatever it is, collateralised faster so that causes issues. Sometimes the experiments that you've got like I guess you've got. Somewhere where she ends up with. Unintended exposure because of this. I mean, are there ways of randomising? In cell culture studies, it should be aware of or situations which maybe you've got homogeneous self suspension. So it's less of a concern. And I just had anything you can think of that can help us understand how you can have confounds and play. And in future studies.

Participant 7
The thing we mentioned that last meeting that you need proper control for, you know stem cell based model to make sure that there are no genetic treats or differences between let's say passage one which you do have from the first time you sell and you differentiate compared to let's say passage #15 which you have real. We banked 1415 times, so sometimes you may see differences between a passage one and passage 15. Would you? Which is perhaps not unexpected, but you need to control for that to. Perhaps you cannot directly compare, let's say, a certain gene expression from position to position 15.

Paul Whaley
Does have any thoughts or comments?

Participant 3
Not really, no. I have to say that today's points are much more difficult than last week's ones.

Participant 7
I feel this belongs to episode is not cells.

Participant 3
Yeah.

Paul Whaley
We think it does to perhaps to a certain extent, but I think we're also thinking that with like primary cell cultures that. That's some indications that belongs and also that there are confounders that come into play even in experimental studies, particularly if there's a lack of randomization, what you end up with baseline differences between groups and somewhere and other. But yeah, the tools that. We got these concepts from like a lot of them were designed for epidemiological or exposure based studies, but we wanted to include them just to make sure that if there were any insights. That could be useful. We didn't miss them, right? So being casting on that very wide and then eliminating things later. So specific things that are talked about, we did actually just touch on this. So by time varying confounding. So by the cell passages, sounds like a time varying confounded potentially. We'll talk a little bit about baseline difference between groups. So I think if no one has any further comments, you might move on to the next. Domain. So we will do that. So that's another one. Was there right in it? So this is Addis early study termination bias. Uh, we found no criteria relating to this in our included tools and none of the 72 tools mentioned this. Hi this is a biassed due to the decision to end these study earlier than planned. So what's happening is that you are observing a, perhaps a positive result, or you think the correct result. So the feeling is that you don't need to complete the study, you just stop once you've got enough data to make your point. So I think the corollary or inverse of this would be like a late study termination bias, where you do the planned number of replicates or repetitions and then you haven't got the result that you want yet. So you carry on until you do. And I think this has been observed in RCTs where there's been some adjustment of the endpoint of the RCT relative based on knowledge of how effective the intervention.Looks at that point. But I just wondering if this is something that. Kurds happen in in vitro context and if it was happening how you might recognise it for example. This might also be an opportunity to discuss how repetition and replication issues might introduce bias into. In vitro studies as well. Some want to maybe. Sure. Do you want to start us off if you have any thoughts at all.

Participant 4
I mean. No, no. I was just thinking it to me. I mean, I don't know this is not, I don't know whether that's biassed to me, of course, it's biassed, but you're not following the protocol, you just do something different. So you just deviating. So I mean the bias this. Yeah, you just think, OK, you wanna save time or you're gonna have done done. You're totally deviating. And then of course, you're introducing certain bias in the study. So I guess I'm, I'm struggling if you. I mean, unless there is some, unless there is a reason why you have to. I mean, if you make a conscious decision to start to stop something early and then introducing bias, if I'm just trying to think, is there some reason where something could end earlier and then you can have to say, well then you can't use the result? Probably. I mean if you have submarines running or something and. You have to run the certain observation period for X number of hours and you have to stop earlier for various reason. Then you introducing some bias but um. I guess that to me is kind of your such a deviation that you shouldn't really use the data. I mean, that's kind of your first point here, ending it earlier late to generate well. If you make this consciously, then yeah, of course then. That creates bias, but can it happen? I guess yeah. As I said, if you have like a a machine failure or that give observation is not captured over. 24 hours, but only after over 20 hours. Then the question is. How do you introduce by island with these? With these output? Can you use it or not use it? I guess that's the only thing I could think about, like some failure of an equipment which stops. During the analysis or stops capturing data. At a different time frame, on doesn't take every. If you have to take a sample every 5 minutes and you're taking it only every 10 because something happens, I mean. They these type of things but.

Paul Whaley
At least the.

Participant 3
But for me, if if there is a defined protocol you know. These things should be easily detected.
Um, if there is no defined protocol?

Participant 4
Exactly here exactly, yeah.

Participant 3
There is no defined protocol then you probably are introducing bias anyway each time you're doing the the the experiment because you're not following a standardised procedure..

Participant 4
Yeah, well, that's why I mean, so it is a bit difficult to to think about that because of these things happen. You're not, yeah. Full stop and that's not correct.

Paul Whaley
So how often is it likely to be the case that there's going to be a kind of prespecified protocol that people have following? So we're going to be able to. Tell when. A study has been terminated early or late. Is this something is going to be difficult to detect? When we have study documentation or is it something is going to be relatively easy because most people? Will have a pre specified. Pressure. Cold.

Participant 7
I think also this kind of bias is quite. Then I don't think it's really applicable to to all, at least in neutral studies, because you do it online this weeks or maybe months after you do the study and you have the exposure to, let's say, 4 weeks, you do the sturdy, prepare to samples for analysis and then you do the analysis. So the. Them on the exposure side or on the protocol has machinery. If you have the protocol, you have the exposure, then you do the study and how can that be by us?

Paul Whaley
Ohh okay. So you're saying that you it would be difficult for this to happen because you wouldn't have sight of the data in order to make your decision about stopping to make the data look better, right? Yeah.

Participant 7
Yeah, right. So you could see that there are some morphological changes, maybe if you do, if you pick up the microscope and do an investigation and will sales perhaps and then you may have and your study at 2 weeks instead of four because you see some interesting things there. But then you may discover that it's really reversible if you go back or if you investigate it. One year later, up to four week time point and see that it was reversible.

Paul Whaley
Because the probably.

Participant 7
It does not happen to me, but it's. Maybe very hypothetical.

Paul Whaley
Very good. So I think we should always, sorry, sure.

Participant 3
The This is why regulators prefer to use standard data than non standard data. Obviously mean if you have a a standard method that has gone through some sort of validation and then a protocol exists, the protocol was examined and you have to follow that protocol. If you have non standard data and you may probably as you said Paul, you not do not necessarily have a standardised protocol. And a well described protocol, then the uncertainty around what's being published is higher. You know, you don't know if already in the published literature it may be consistent across what's presented in the paper, but it it, it may not present the whole truth. Um, but you know that that that is a more, I think a question of uncertainty than a. Then a question of bias and it's it may not be easily quantifiable. Unless you actually try to. To reproduce those data, I guess.

Paul Whaley
Yes, very good. Thank you. So how do I just wanted to. Make sure we highlight in the the notes this issue of when data collection happens sufficiently after the. Cultures have been. Rather, I guess for one of the better phrase. That you wouldn't think that the researchers could have had sight of the data, such that that would then influence that decisions about when to terminate the study.

Participant 3
Ohh, but even that I mean you could you could actually collect data on. You know, a certain period of time and making measurements across those time points and then later on when you're analysing those data. Um and you see that the picture is more convincing at a certain time point. You disregard the rest and you only publish. Those time points so you could actually do a preselection afterwards. And this is not early early study termination bias. It's probably another type of bias we already discussed, you know, selection of what you report you are reporting. But you know why it's related, right?

Paul Whaley
I think all the biases tend to be heavily interrelated. In fact, he's very rarely see one without another in play as one of the things that makes it so difficult to analyse empirically as to how much they matter. So yeah, he's extremely good points. Thank you. Alright. You have to move on to. The next to my actually got one here that is hopefully a little bit easier on it. So performance bias we had 44 criteria from our. Uh. Precluded tools. A performance by us is defined as a bias resulting from differences between the received exposure and the intended exposure. So does anyone have any questions, thoughts, comments on this, and what it might mean for in vitro context?

Participant 7
If you do a robot to do your exposure, it may lead to less variance than you know. Manual pipetting or exposure. If you compare and comparing different labs or yeah for a defined set of concentrations.

Paul Whaley
The patrol charity.

Participant 3
Well, I think we, we met I we missed this already today in a different point. You may intend to expose cells to certain concentration, but then you have binding to. Medium protein to plastic to and and the actual concentration that the cells see is different from what you actually intended in the first place. . So you know you should do these. And cause it take these considerations and there are models to to predict this even in silico models, you should do it up front. Otherwise, you may actually. Be basing your assumptions or your conclusions on the wrong premises. But I I don't know if that's what's meant here. Um, but yeah, actually having a a good, a good understanding of what the actual exposure is in the cells is important.

Participant 4
To me, I agree with. Was just thinking we we had that discussion. I mean it's all about pipetting errors, I mean some. Not, I mean, not calibrated pipettes, volatilization of chemist chemicals going off. And you don't give to cells or in whatever experiment, not the right exposure. So it's kind of similar to what we had before, I thought.

Participant 3
Recipe.

Participant 4
Yeah, exactly. Yeah, that's yeah.

Participant 3
Jason is also an important thing, especially for lipophilic compounds and other things. When when you're doing exposures in liquid cell models you know. And you quite have quite often have a problem of of. Ohh overpredicted exposure because you actually the sell the the cells never see the concentration you you added because of precipitation or other effects.

Participant 4
Solubility. Yeah, so here.

Participant 7
I also think the normal concentrations depends on the volume or you're adding to the sales sort of because if you do, even though you doing a 96 level plate exposure, you may use from let's say 100 microliter to 200 microliter per well. And then you might have a double the volume of your test component in the 200 microliter experiments compared to the 100. That's why you would like to perhaps to go all the way to the on me, to team extra place and to really know the exposure, the intracellular exposure instead of using the normal one because they can really differ depending on the experimental conditions.

Paul Whaley
Yeah. So we definitely covered quite a few concepts and performance by us last week and our discussions. So I'm just going to flash up five things that came up quite regularly. In the tools that we abstracted criteria from. So there rushes around cell culture conditions, so the medium and maintenance for example. Um investigator knowledge of exposure group and if that affecting, how the? Ohh cultures might be handled. Era in test substance, so applying the wrong substance impurities and test substance. So the test substance not. Results I guess being compared to some extent by impurities and then also issues around the solubility of the test substance resulting in the true exposure not being intended exposure. Just wondering if. We've had much discussion of culture conditions and their potential impacts on distortion results for study. Or. The possibility that an investigation so the reason that investigation knowledge has come up, is that in in animal studies, for example, what can happen is that because the investigator knows that this cage of rats has being exposed to the carcinogen and this other cataracts is not that that results in differences in animal care practises where the. People responsible for animal care. Look after the rats exposed to the carcinogen. Kind of better so they get better care and that ends up distorting themselves. The study because you've got an like, the exposure in the broad sense is not just the exposure that a carcinogen, it's also all the other stuff that happens with around the rats as they're being looked after. And then you end up with a difference between the rats that are given the carcinogen, terms of husbandry versus the rats that are not. So the rats that have on the cousin would do actually slightly better, maybe they would. Otherwise you see fewer harmful effects. Is there anything in analogous to that in the in vitro study design, obviously? Cell cultures aren't particularly cute, so they're not going to necessarily round the same emotions in people as a rat might, but other things that people could end up doing differently because they know that this is the, you know, maybe the more exposed culture versus the less exposed resulting in attentiveness or differences in. How the? The cultures are maintained or maybe where that puts in like incubators and things or anything at all like that. Suppose this silly file fetched.

Participant 3
How how would you detect that actually mean? Even even if there is a bias. I'm not sure how you would be able to detect it.

Paul Whaley
What do you call necessarily detect biases? This is one of the issues with them. Because he might not have data that allows us to be done. But you know, we've heard that what might happen in some labs is that you've got. Like a multiple racks and incubator and they just always put the highest concentration exposure cultures on the top and they just work down. So there's always a tendency high to low in the incubator, top to bottom and then the temperature at the top of the incubator is slightly higher than the temperature at the bottom of the incubator or something like that. And then consequently you get different. Like I guess maintenance conditions for the cultures in the incubator because they've got a pattern of distribution that matches the passion distribution of exposures saying. It might be incredibly hard to detect, but it's been shown to be important in animal studies, so we want to make sure we're aware of any possible issues without necessarily worrying at this stage about that disability.

Participant 7
Who, regarding the bullet point, Emma three-year the hearing test substance. You obviously want to have a test substance which is stable and. Yeah, and do not degrade during your test period, so making a period or you you want to know, then the stability of your test substance. Obviously for GLP study you have to test the stability. And you you you need to prove that during your test period that yours substances you know at the same. And the purity and stability during your study, I guess for the normal kind of university studies lab studies, you do not really have that information. So sometimes you could use tips instances which is not stable throughout your whole test period.

Paul Whaley
Very good. Interesting. Thank you. Does anybody have any other comments that Participant 4 or shall? Some of the things that um. Does come up in the tools that we've been looking at the I wanted to cheque on specifically. Includes um. Issues around allocation concealment, so not being aware of which are experiments are unit, has been exposed to what dose. Is generally seem to be quite important in. Certainly in vivo and clinical trials. I think there's something's been highlighted as a potential for visual differences between groups in vitro that might. Give people information about which. Um of the cultures is more or less exposed perhaps. So they've got prior knowledge of the dose group from that could affect how they handled the plates in terms of maintenance or interpretation of the data and things like that. Um, it could be the so. I don't know if you're going to see differences in methods of exposure administration between groups if that's ever going to happen in vitro. If the. Vehicle choice of vehicle can influence the. The amounts of exposure or the type of exposure received and then I think we just touched on robotic test systems already as being one way in which. Explosion levels can be more precisely controlled. Obviously, robots don't generally have any expectations about what they're doing either, so. Do any of these things like is is masking of investigators common to all in in vitro studies? Could could be influential and potential for bias? Any thoughts on that? This would relate to the handling of the.Of the. Either the way exposures administered or the handling of the cell cultures, not necessarily anything to do with the interpretation of data at this stage.

Participant 7
Yeah. Do you definitely need to assure that your vehicle does not evaporate from your, from your Wiles when you're doing the testing? Or let's say a couple of weeks? That's something I've experienced actually, although they they are, you know, supposed to be closed and capped and everything. You need to cheque whether you need to make up your test solutions everyday, you're doing your experiment or really make sure that this does not evaporate because then you expose your system to different concentration than you did two years in days ago..

Paul Whaley
So this is a change in the vehicle overtime. So this is new for us.

Participant 7
Yeah, yeah.

Paul Whaley
Yep.

Participant 7
Or actually, the concentration of artist component because of the changing vehicle..

Paul Whaley
Sure, I will catch you have any thoughts on this?

Participant 4
No, not much more. And it actually no.

Paul Whaley
Okay. And then we have the other domain. So this is about distortion results due to factors other than those described, but so this is going to be pretty much anything that you've. Can think of that we might not have already discussed. So two things that we weren't able to allocate to any particular bias domain. Were the choice of source of activation system given test article and then the inherent physicochemical properties of the test substance? We didn't really know what this meant, to be honest, so if anyone has any comments that can help us understand these two, that would be really helpful.

Participant 4
I think the letter won the second one. I mean this is related to some of the things we brought up. I mean, if they're not soluble, if they are not, then you don't reach the right exposure, which you intend to have. I mean these type of things. And so it goes by me. You can just flip this around and you have exactly the same examples we we talked about before. Have they don't get resolved, they they participate with drawers saying ohh they evaporate or all of the above so that all relates to their physical properties and then you don't reach the exposure or you don't have. I mean whatever this was called before and remember that. But we had this discussion about not reaching the right exposure to the intended. So if you flip it to me that fits perfectly there.

Paul Whaley
Okay very good.

Participant 7
Exactly. And some people says, for instance, they do buying quite strongly to proteins while others do not. So that will lead to difference in the exposure as one example.

Participant 4
Ohh that discussion we had about the the binding to plates and all the all of this. I mean this is all related to the properties of the subset..

Paul Whaley
So what about the choice of source of activation system given test article? Do you? Do you have any insight into what that might mean?

Participant 7
I guess that could be a metabolic metabolic system like, you know, it's nine fraction or something. Is that what the question is about? Because.

Paul Whaley
We I don't know how this is just a criterion that was abstracted and we are stumped so.

Participant 7
Obviously, some of the metro systems, they do have a quite low metabolic system. By transformation compared to, for instance the livers or. I assume that some have tried to include I. By transmission system based or built from liver fraction to speed up the metabolism in or he needs to system. I think that will also lead to a lot of other. By us is or you know confounders. But then my. Yeah.

Paul Whaley
It's like a jump start for the culture, right to get it, doing things a bit quicker.

Participant 7
If you want to buy a transform your component. That you know, it's by a transformable or you need a by a transformation to give a certain effect to. You would like to test that perhaps.

Paul Whaley
OK. That's not possible.

Participant 3
I think that there's another type of bias that could introduce going the opposite direction of what authorities said, because when you use S9 quite often you're biassing towards activation, but it is regards detoxification because you have your lacking face too, or or the Co factors are missing and therefore in a living system where you could actually have detoxification of the compound you're only introducing. Activation basically. Through S 9, so you're. May actually have. A picture that is not entirely the truth. Um, so that that could also, you know, to circumvent the problem, you may introduce another type of bias by using S9 sometimes..

Paul Whaley
That's seems very plausible. OK. So we have finished with our set of domains. So we've got through those quite quickly. Thank you for your patience as we work through some of the more difficult ones with fewer items from the literature. Appreciate that's quite taxing work and quite confusing. So comments are very, very welcome as always. Do you have any final thoughts or comments before we wrap up for today? So in that case I'm just going to say thank you very much for your time. Again, very much appreciate your energy and input is really difficult task. These focus groups are the hard work, particularly for the participants because you don't really know what it says that. We're not really guessing it anything, but it's not a very natural exchange because we we don't really know. We're trying not to be leading, so yeah. Yeah, we'll be back in touch with the. Results of this stuff as we go forward. And I think and do you have anything you want to add to that?

Gunn Elisabeth Vist
I just wanna thank you very much for coming up and sharing all your experience videos. Thank you.

Paul Whaley
We are guessing a huge list, so we have been doing some preliminary analysis of the transcripts from the last three focus groups and the number of additional bias concepts that. Being added to the ones that were in the literature is I'm not saying it's worrying us from the data management perspective and how we're going to make sense of it as we try to put together the first draught of the assessment tool for the Delphi process. But we're very excited about the prospects for being really much more comprehensive than anyone else. I think it's previously been and your input into that has obviously been invaluable. We really could do without you. So thank you very much.

Participant 3
I'm afraid, Paul, that. By applying your tool, you'll find that all studies are biassed because they're. That so many so.

Participant 7
I didn't know that everything is so many biassed as I learned here.

Participant 3
Yeah, there's so many criteria and some that I personally, I hadn't even think about. Yeah. And especially when analysing non standard data literature data. Um, you know they will not fulfil many of those criteria. So yeah, it will be interesting to see what comes out of this.

Paul Whaley
It's going to be tough. So yes, stepping back from it and thinking about how it's going to work out in practise, it's it's, it wouldn't be the first time that when a study assessment tool looking at internal would have been developed that you see quite a large number of potential issues with a majority of published studies. Then there's a question of course of how you appropriately use that information and then how you use that information only to make sure you're interpreting studies appropriately into systematic reviews, but also how that then gets rolled into. You know, new guidance and recommendations and quality control processes for research to make sure that important issues that are being open, yeah.

Participant 3
Right.

Participant 4
And to draw his point, I guess we have had bias before in the old studies as well. So you have to find a because if you know kind of trying to remove everything that's an yeah, that's pretty impossible. I guess you need to figure out like. What is critical and what not to be able to use the information?

Participant 3
Right. And and actually benchmarking against guideline studies for instance.

Participant 4
Now exactly.

Participant 3
Would be interesting because even I I'm sure that even on those for those studies, guideline studies. Done according to DLP, following a protocol and so on. Some of these biases will still be there. Then you have to think whether those are important or not, right? So benchmarking against those would be important also to assess the value of the tool. And calibrated so that you know you don't have excessive bias all the time. I'm showing up.

Paul Whaley
Absolutely. Yeah. So this isn't an exercise in coming up with 600 ways of telling people. They did research badly or saying if you got one of these 600 things that people think might be important, that this study somehow no good intent is absolutely not that. It is to come up with a. A useful way of structuring. The appraisal of the study so you can figure out which ones you're less worried about from a biassed perspective, and ones that you're so worried about that you don't really want to put much weight on them when you're doing your overall, like systematic review or. You're assessing your body of evidence. So it's definitely definitely not going to be a situation where everybody looks bad and we've got silly criteria that don't really matter ending up in the final study. Yes, supposed to. Hopefully going to be a very manageable set of questions. Which can be answered by people which provide useful information about limitations and study design that. Would be of concern from the perspective of introducing systematic error and then that information about the potential systematic error can be properly accounted for. In the final systematic reviews and the final risk assessments. So it's not intended to just smash up every study and make everybody look bad at all.

Gunn Elisabeth Vist
Absolutely. And it might even. Might even end up being a good, good set of a list of what to avoid. When you do your studies.

Paul Whaley
Indeed, so it can be helpful for guidance. It can be helpful for understanding you know. Where we're at and how well studies are being conducted and just these sorts of things. So anyway that is very much for the next phases of the work. We're still in the discovery phase now. So we've got two more focus groups this week. Uh, and then we've got the job of trying to build the first version of the tool, which we can then put to the Delphi process, so that will have a quite a long list of criteria in it that we take from the literature and from the focus groups. And then that Delphi process will refine that list of criteria in terms of which seem more important and how they should be phrased. So we can then start getting close to an actual usable tool. Right. So the initial that dress that will have like 9100 criteria in it, and we need to eliminate. 40 to 60% of that and must have something manageable so. And useful, right?

### Focus group 3, meeting 1

Paul Whaley
So we're going to start with performance bias. So we found 44 criteria in the literature that could be kind of associated with this particular bias domain. We define performance bias as a bias resulting from differences between the received exposure and the intended exposure. So when it comes to in vitro research contexts. Why is do you think and what would this mean to you in terms of how the exposure you intend to apply and you're in vitro study environment could be different from the one that you were intended to supply?

Participant 1
So for example, if you have a chemical that may be easily dissolves in DMS O, but overtime crashes out of that DMS because it's absorbing water. Naphthalene is one for example that does that or could also be it's kind of Can sublimate, so overtime you don't have the same concentration of this particular chemical in your in vitro system as you thought you did, and so maybe you don't get an, you don't see any results and you think ohh Napoleon's not doing anything, but it's actually. Doing something might be doing something, but we don't know because there's no napping left in the sample at the end of the treatment window.

Paul Whaley
Thank you very much. I if you want to speak, you don't necessarily have to raise your hand. You can just jump in. There's not so many people on this call that would be unmanageable. So yeah, please do just chip in whenever you feel like it. I didn't see a couple of knobs. I might put someone on the spot. Say Participant 2, for example.

Participant 2
We actually tackle this type of issue in a in vitro study. There were some follow up studies on this. It was on development of New York City and the effect of your period was just to give you an example completely so. Basically the taken message was that rather than considering the nominal concentration, the one that you think you have created, you know with your solvent, whatever it is, the Ms in our case, it's always important to really measure the intracellular extracellular concentration on the chemicals. So of course this is not something that easily doable everywhere in every lab. But generally speaking, this is the way forward. We found some differences sometimes, and yes, it's the solvent..

Participant 2
Maybe the bind into the plastic and maybe the you know how much chemical enter the cells, how much remains outside. If it's get some turbulence, there's these additional considerations for quantitative English translations, but generally speaking, the nominal concentration is 1, so I think it's very important even in publications. For example, peer review publication to always specify if the concentration was nominal or concretely measured in the in the test system.

Paul Whaley
Participant 2. Thank you very much, Participant 3. If I saw you nodding quite vigorously as well, is there anything you want to add?

Participant 2
Yeah, exactly.

Participant 3
Yeah, I know. I think everything is said, but yes. But also it depends on how often you actually exposed the cells and how you expose them..

Paul Whaley
Do you want to expand on that a little just to kind of give some example?

Participant 3
Ohh yeah, for if it depends if you want to have like 24 hours or 12 hours depends on how long you want to expose your cells. If for example we are in many cases you're running 48 hour exposure and then we will actually exchange the medium after 24 hours just to make sure at least that the concentration is more but you want..

Paul Whaley
Thank you very much. So have anything to add?

Participant 4
Yeah. Ohh, one of our concerns also we've had and research is what we're dealing with complex mixture. So I have like a tobacco research background and not everything was actually soluble, right. So when we're doing these exposures and trying to bubble the tobacco smoke into media only what can go into that aqueous phase actually gets exposed to ourselves. So that's something we've always like worried about is when we're saying that tobacco smoke has this effect, well really it's only what's going in the aqueous. These were losing a lot of that. The volatile component and you know pH and stuff. So that's also one of those concerns that we always have in our research. We're trying to think about how relevant is it and then when we compare it to and in vivo model, they're getting something you know different as well.

Paul Whaley
Thank you. Thank you very much. OK, so things that came up kind of quite regularly in the tools that we had included in our literature review. Alright, things like cell culture conditions, so condition of the medium and maintenance. So these are what you might think of as sort of. Not they're not the exposure in so far as the intended exposure of the the say test article or the chemical the are exposed and say but they are part of the exposure conditions. So the cells are exposed to everything in the test system, right. They're exposed to medium and in a way they're exposed to maintenance conditions. So part of performance bias concerns. Like how well the cells have looked after, right? The cell cultures it looked after. There's some concern that investigates and knowledge of exposure group could be important. I'm just saying maybe that we want to focus in a bit on because obviously we know from like a human trials and animal studies that if the investigators are aware of which.
Arm of the study, the either the subjects have been animal or a person as in can affect say the care that is provided by the investigators to that participants you've got rats and you know that the rats in this cage are exposed to the carcinogen. That might result in people paying more attention to the husbandry of those rats because they're worried about them. They never gonna get sick. They're gonna have a pretty rough time. So they treat them a bit differently to the rats in the control groups. Is there anyway in which in in vitro studies that investigate and knowledge of exposure group could impact upon? But how the cells are looked after cell cultures looked after, so to speak.

Participant 3
Ideally you should randomise all your groups and then you would kind of eliminate this. But of course this is also causing a problem if depends on where you want to put the chemical afterwards. But. No.

Paul Whaley
Hmm.

Participant 1
And thinking back at my to my days on the lab and I. Don't think so. I don't know if I'm just being naive or I'm remembering just being a tired graduate student post doc when you just kind of you know you you have your plates, you you expose them and then you just kind of put them back in the same incubator. So I mean, I don't. Think so?

Participant 3
Yes.

Paul Whaley
Could there be issues when it comes to the incubator itself whereby? Yes.

Participant 3
It depends on how big think better and where you put it in the in in Coimbatore and also where you yourselves are or what group are in on the plate..

Paul Whaley
Hmm.

Participant 5
Yeah.

Participant 3
That will affect you having the edge effect.

Paul Whaley
Effect.

Participant 5
Exactly. I was just going to say that the maybe you every time put on the exposure in the same order on your plate. So that could lead to bias actually with the plate effect. So yeah.

Participant 2
But generally speaking, just about to this, the volatility of the compound should be considered not all compounds and chemicals are volatile. Therefore, this may be a clear issue if you have volatile compounds, but if they're not, it's probably less of an issue. So even Edge effect or having cross contaminations with control? Wells untreated or solvent? Solvent control wealth could be definitely an issue when you're dealing with volatile compounds.

Paul Whaley
Could that be issue?

Participant 3
Boss evaporation. From the wall.

Participant 2
And operation. Yeah, sure. Especially for the outer wells. Right. So that's really a problem. Normally to overcome these issues, we use this kind of semi permeable membranes. On top of the whatever plate format and those were allowing clearly exchanges of gas. See you 2 you need to etcetera, but preventing the cross contamination or volatility issue.

Paul Whaley
Hmm.

Paul Whaley
Participant 6, do you have anything to say? Been quite quiet so far, so I'm just gonna put you on the spot, but just want to make sure you're getting to contribute.

Participant 6
Yes, thank you. No, at the since I don't work experimentally in the lab, I I don't maybe don't have as much to contribute from practical experience as the others, but I was just thinking about impurities. Um. Which could of course if you don't keep track of them, could cause effects that are not compound related, which is the opposite of, you know, not not getting the expected effects from the compound that you're added you're adding.

Paul Whaley
Yes, thank you much. So some things that came up under performance by us that we wanted to cheque with you. Ohh included issues such as. Uh, the breaking of masking or blinding procedures in an experimental study, so I don't know how. Frequent or necessary, this is in in vitro studies. But. And certainly, and other experimental contexts, it's important to blind the investigators to the exposure status of the. We'll call them participants or test subjects the the the thing to which the exposure is being done, right? Sometimes you can get visual differences between groups. That can also mean that the. The allocation of the OR the exposure status of the. Participant is is known to the investigators. Is this sort of random allocation thing something that happens in in vitro designs? Is it? Does it not happen because it's not practical or does it not happen because it doesn't matter? I'm just wondering what your thoughts are on this.

Participant 2
OK, this is based on my own experience. To be honest, we were, especially when testing things in House for just. I don't know small experiments and stuff like this. We were not really blinding the experiments, so we knew exactly what we were treating the cells with, the how concentrations were distributed, etcetera. But clearly this is an issue when it comes the time to perform like a validation study. . So especially in trial interlaboratory reproducibility testing of a certain set of chemicals, in those cases, everything was wine coded. And whatever was getting to the laboratory in another laboratory was totally blinded, so they did not know what they were dealing with, except for a kind of molecular weight, which was not exactly the molecular weight of the compound, as you know, with common everything with clear details that could have been. And they could have allowed the identification of the compound itself. But it was like a rounding up the molecular weight and. So this is what was done and clearly for validation. This is something that has to be taken into account.
For internal studies, it should, but practically speaking it's not done, at least from my side. Based on my experience, it's something that we don't do regularly, but we should probably, I agree.

Paul Whaley
Thank you. I see a little bit of nodding. Does anyone want to? Follow up that any thoughts of their own? I could make somebody follow up. Do you have any additional thoughts?

Participant 3
Um. Yeah, usually it's a post doc or a researcher just following up their own studies. So we don't have any blinding yet. With sending off samples for RNS sequencing, sometimes then, but they are labelled, there are limbs, numbers, so maybe that make it more difficult for the analyst to figure out, but. Yeah, no, it depends on the resources as well, so.

Paul Whaley
Hmm. Participant 5, you wanna you have any thoughts as well so you nodding very vigorously. So I had to put you on the spot too. So it's not just.

Participant 5
Yeah. No, I I agree. I think probably should do it, but for practical reasons, it's not really done. I think it's the same researchers that that put up the experiment and that analyse it often. But if we send for external analysis, then it's easier to do this type of randomization or yeah, binding. Um, yeah.

Paul Whaley
So what makes it?

Participant 5
So it's a good idea, but maybe we are not doing it, yeah.

Paul Whaley
So what makes it not practical? Ohh. In your opinion?

Participant 5
I think it's. I mean, if you really should do it blindly, then you need to be one person that puts it up and another person that you know do the the end analysis and that's not maybe feasible for projects where you have just the post doc that is working and doing all the job themselves so. . Um, but I guess it depends a bit on what you are doing. If we are doing immunolabelling then we often have more possibility to to do the blinding because maybe it's not the same person that do for instance the microscopic or analysis afterwards.

Participant 2
Who?

Participant 5
But if you do. Yeah, some other more simple like viability assets or something. Usually it's the same person that does everything..

Participant 2
Yeah, that's a good.

Participant 6
Maybe. Yeah, I'm sorry.

Paul Whaley
No.

Participant 2
No, not tonight.

Participant 6
I yeah. Yeah, I know. Maybe I can just. And one thought, and I guess this is maybe more about the exposure and making sure that the OR are are confident that the exposure is what we. What? What blinding for the exposure, but in terms of then looking at the outcome, I guess it depends a little bit on the how critical it is that the people are or that the person is blind and depends a little bit on how you measure what types of effect you're looking at and how you measure that if if it's completely automated? Then of course. And the. That interpretation of the results is, you know, less. Left, there's left less risk for bias from from the evaluator. If you know everything is automated and. In terms of reading the output compared to, if you're looking at if you're, if you have the visually evaluate or counsels or whatever, and then if you maybe know what the exposure was, you might be biassed towards, but if it's like automatically measured, then you have less of a chance to influence the outcome anyway.

Participant 2
Yeah. And if I may add to this. The analysis I mean the assessment, the quantification process, if it's automated exactly as you said, if it's done in automatic way, a play treader, whatever it is, high content imaging platform. Clearly the possibility of bias, whatever you get is the number. So that's it. But with regards to plate, plate layout design Um and distribution of chemicals. One thing is if you do it manually, another thing if you do it with the robot, sometimes even programming a robot for dispensing chemicals, even in a completely blind fashion, can be complicated in terms of programming. Um, rather than having, you know, distribution of chemicals dilution steps performed using the robot, etcetera. So I think it's not just the personal. So the number of person dealing with the experiments or or the other aspect we have touched upon, but it's also the programming skills we need people with good programming skills, especially when you're testing a lot of compounds in kind of a higher throughputs or medium high throughput testing project or study. And therefore you have to test many chemicals at different concentrations on different 96 or 384 well played. So there are different aspects, even technological programming aspect to consider..

Paul Whaley
Alright, good. Thank you. OK, So what we'll do now and move on to the next domain, so attrition bias, so this came up less often in our. Included tools in our lecture review, so we only had 16 approximately 16. You need criteria relating to this. So attrition bias is defined as a bias due to absence of expected participation or data collection after selection for study inclusion. Obviously this is definitions that are written primarily around clinical trials. But I've got language of participant and inclusion. So just to interpret it for us here a participant would be something like a plate or like a multi well plate I guess. Then selection for study inclusion would just be the process by which you have. Maybe you're prepared. Plates and then some of those go into the study because they've been prepared properly. Some don't, and we're talking about things happening after you have your participant. Resulting in them dropping out for. Whatever reason so and it could be that the data isn't being collected from the plate, the plate is dropped out of the study. Whatever. Happens in that space. Does that make any sense to you to have any thoughts or questions on that?

Participant 6
Is this, like, love apples that maybe get lost? Sort of in the? Analysis process where could it also be afterwards, like removing outliers and? Things like that.

Paul Whaley
So.

Participant 6
OK. Ohh.

Paul Whaley
I think yes, we're not really sure, which is why we're talking about it and it wasn't. It was much harder to understand how concepts of attrition apply and in vitro setup. So if we've got, we'll talk about apples, then you've put them through your like they've gone into your experimental apparatus, whatever that looks like. And then for whatever reason, either you're not. They they become dropouts like so in a in a clinical trial, people might drop out of a trial because they don't take very well to the treat intervention. It makes them feel poorly, so they stop taking it all, though if it's an advanced, say stage cancer trial, then there might be dying before the end of the trial's complete. So these will count as attrition. And if they're not accounted for properly, then you get a difference in the study population for which data is collected. Then the study population.

Participant 6
Ohh.

Paul Whaley
That actually was entered into the study in the 1st place, so it can cause problems, but we're not really sure how this how this happens in the in vitro test environment. So would there be situations which you have maybe plate contaminations you're getting? You realise halfway through or plates kind of looking a bit weird so you just get rid of it and then then you don't collect data from it and then you have a systematic difference between the plates that have kind of gone through and ones that haven't this making any sense.

Participant 2
Hmm.

Participant 1
Yeah.

Participant 4
Yeah.

Participant 6
Yeah. Yeah. And I guess it's it it, I mean, I I I, I guess it could maybe try to make the the parallel there with lost temples and things, but I guess also and and people working can correct me but that you can also I mean in this setting you can also really do and replace to a certain extent. So it's not and of course the plates or the cells themselves don't they don't choose to drop out.

Participant 1
I think you also start running into this idea of the difference between a biological replicate and a technical replicate.. And so, and sometimes I've noticed in studies where they don't make a difference, they don't differentiate between a biological replicate and a technical replicate. And so if you have a situation where for whatever reason, one plate out of X money got contaminated or, you know, something happened, cause sometimes you know the stars and the moon don't align quite the way you've thought they should and a plate dies. And all those cells you know don't do it like what everybody else is supposed to be doing. I don't think it's unusual for researchers to just drop that plate. And as was mentioned, somebody just replaces it, but then you and they don't think anything of it, but it's not the same as the others that didn't get contaminated, perhap perhapss. Because it might not be the same like so. Like I can't think of the word, but like uh. The same generation, right? It's it's not like a. The same generation as the others that went through. So it's cause you've divided the cells and you've, you know, played them So.

Paul Whaley
Umm.

Participant 5
Hmm yeah.

Paul Whaley
Ohh, you're having your little thing.

Participant 4
Correct. You know, I think of user error.

Participant 1
Yeah.

Paul Whaley
Sorry, Participant 4, go ahead.

Participant 4
Ohh I was saying. Yeah, when I hear this it's you know, handler error whether someone's blowing out the well put. You know, thinking of a grad student rushing through and, you know, feeding too fast and they hit the bottom. And I've seen that happen in my lab and you lose your cells that way or someone forgetting to feed their cells over the weekend and you lose, you know, one of your experimental replicates due to that too.

Participant 5
Yeah. So we are working with Ollie lifted cells, so air interface. Epitelous sauce and in that case we we want them to reach a certain resistance over the epithelia. So in some vals you don't the cells don't reach that they they don't get the tie junction and you don't get that. And in that case maybe you lose some wells. So this is I think this type of bias. And if that is the case for the wells.

Paul Whaley
Hmm.

Participant 5
And you end up with too few technical replicates. For instance on the control. So and you need to add controls in another experiment. That would be a different passage and it could of course lead to bias, but this is not really something that I have thought about honestly, because this is something that happens in this type of experiments sometimes. And it's not something we can really control. So yeah, it's very interesting. Hmm.

Paul Whaley
I mean, these are just ideas and concepts that have kind of flowed into our system from the tools we reviewed and we don't know if they're in there for good reasons or bad or if they're. Really apply or not because the tools that we've been abstracting criteria from the very mixed and come from all sorts of different sources and modified and different ways. So we just have our bag of things and we're just trying to discuss them with you. So this is really interesting discussion. Thank you. Did anyone have anything else they wanted to just like pull up some? Examples. So I think we've talked about exclusion selves from now on. This little no, no, maybe we haven't actually talked about that very much. So when we talked about lots of samples for sure. But what about the situation where? You. Get some data from your plate and then you like. I don't like the look of their so you don't put it into the analysis in some way. So I think this could have something to do with outliers. But. I'm just wondering how this might introduce distortion or. Bus into a study.

Participant 2
I mean, this is really common practise, right? Excluding outliers which really do not make sense. But based on my experience. Um. Talking about apart from, you know, internal replicates, biological technical replicates, biological replicates, etcetera. As as much as possible, we were trying to retain all the data including the outliers and see how the general trend of the graph is looking like. If one data point in one specific internal technical replicate was completely, you know out of the blue, then it was discarded. Also for statistical purposes, right? Because maybe it just that one was screwing up the statistics. Etcetera. So this is generally speaking the approach trying to keep the outliers as much as possible in because anyway they may be relevant. Why shall we discard them up front? But if just one or a couple of them do not make sense. Um and screw up everything then? Yeah. Then normally we were excluding them. This was the general approach based on my experience.

Paul Whaley
Umm. Will there be any grounds for exclusion from analysis? For reasons other than. Looking like outlier data, potentially.

Participant 3
If you know that you have a mixed up there said exposure, then you're.

Participant 2
Ohh.

Participant 3
After exclude them.

Participant 2
Yeah, I fully agree. I mean, if you're up front that something went wrong, then don't. Don't count. Don't count them. But if you if you don't know, and apparently everything went OK. And how can we decide? Then it's really the data speaking selves and so. In the end.

Paul Whaley
OK. So in terms of specific things, one of the things we weren't quite clear about is the role of the cytotoxicity of the test compound. How that? You know you you end up with cells, cultures dying and then how you handle. Situations where you see. Yeah. What? Just just what taught me through you, if you like what goes on with this and and if it looks like size toxicity could relate to attrition bias in anyway. So you've got a more. Issues with diaphragm. More than more than just outliers, but like some kind of something else kind of going on, some pattern of. Is she there?

Participant 1
Ideally, people should be performing a cytotoxicity assay right to find out what the dose is that they isn't going to kill all of their cells. So I mean that should. That should be stuck on I think. So in theory this shouldn't be happening.

Participant 2
Yeah, cytotoxicity assays are. This is normally what we do first. So those response, Curt, you want to figure out, you may have a these kind of range Finder approach where your test, the chemical with the quite large dilution stuff like 1:00 to 10:00 and then you may refine it one to three and this is common approach common practise. But yeah definitely you have to the idea is always to avoid working with cytotoxic concentration. Most of the time we were selecting like I see 20, but no more than I see 20, but this was this is what I was doing in the past. So ideally we should yeah definitely avoid cytotoxicity and working with cytotoxic compounds.

Paul Whaley
Umm.

Participant 4
Correct.

Participant 2
And then another.

Participant 3
I have been using the. Usually I've been using the 70% uh. If it's more than that. Reduced than the. I mean, if you're reduced more than 30% from 10, you should. At least for when we are doing the screening, we usually stay in the Nonsuch toxic area. If you are around 70% then then you should be careful because it can. You can up in the apoptosis area there. And that's at least we don't want to be there.

Paul Whaley
Could there be issues relating to inappropriate exclusion of data because of? Like would there be? More or less appropriate ways of dealing with like a baby around that area where it's kind of it is such this levels of. You know more social maybe you would like, would there be way? Could there be issues with researchers excluding that data from? Their study, or from the analysis in a way that was inappropriate rather than appropriate in some way, I'm not really sure how this works, but.

Participant 2
I mean I my reply may sound really naive, but if you're dealing with a side to toxicity and excitotoxicity okay, it's 50 percent, 70% whatever we may think about potential interest in threshold to acceptance criteria where experience criteria. But then the data should be discarded because whatever biological endpoint you're measuring will be definitely impacted by the side of toxicity effect level. Jesus, you know, necrosis effect. So an important element that we were always looking is the time of exposure here, because oftentimes you see publications where they test hyperflex CCC after. I don't know, maybe an acute exposure of 2448 hours and then actually the biological endpoints are all measured after a week of exposure or even maybe just 3-4 days, five days or even two weeks of exposure, which is nonsense. So it's always important to understand. What is the concentration you're testing after the very last time point? And this is something that even as an associate editor, have been asking quite frequently in in vitro studies, why did you test your side effects CT only the beginning. And I don't know, after 2448 hours, if in the end you you have done everything those exposure for two weeks for example. So this may sound like a naive aspect, but it's not. Actually many people tend to tend to do cytotoxicity quite I mean. Early in time, you, my, my perspective and this may lead to this type of situation where in the end you're dealing with a cycle cytotoxic effect and you are mistaking it and you're thinking that you're actually having a biological effect on your compound, while in the end is just like the dogs. You get that.

Paul Whaley
Interesting. It's kind of like the opposite of attrition. You retaining samples when you should be eliminating them.

Participant 2
Exactly.

Paul Whaley
Yeah, okay.

Participant 1
No, this kind of reminded me of a study that I. Um that I read when I was a graduate student and this particular researcher was using was doing diabetes like arsenic. As a diabetic, gin research in molecular mechanisms of that. And I remember his lab dosed these cells with like. Parts per million of arsenic, which is very toxic. When I was using arsenic in the lab, it was in the parts per billion range. So it was a much smaller amount. And I remember he was dosing the sales to this massive amount of arsenic for four hours and then running his. His molecular experience on those in in, in, you know, trying to show how glucose uptake was affected and all of these things, but I just thought your cells are dying because but he's like ohh at 4 hours they're not dying, but that doesn't, they're not dead, but they're not doing well, you know. And I remember my my graduate mentor saying, you know, if I, you know, bopped you upside the head with the sledgehammer, maybe you're not dead, but you're not doing well. And that's kind of what these cells were going through. And so the results that were. Being put out, we're not. There weren't accurate because even though the cells weren't dying, they were not doing well, and so it was compromising the entire results of of what he was trying to prove. And so I think. The time of when cytotoxicity I guess circling back the the time when cytotoxicity is measured after exposure I think is also important.

Paul Whaley
Good sleep

. Well, thanks very much. So Next up we have detection bias. This is one of the most common I think, if not the most common domain that came out said 80 approximately. You need Christ area relating to the potential for bias relates to detection. We define detection bias as a bias due to distortions in any process involved in the determination of the recorded values for a variable. So the variables can be dependent or independent variables. Um. Any process whereby the value of. A variable is recorded right? Silent. Meaningful to you. Have any thoughts or comments as might apply in the in vitro context? How it can get systematic error in detection? Either exposure or outcome or anything.

Participant 6
Well, maybe this is where we it comes back a little bit to what type of. Variable outcome. It is that you're looking at and whether or not you have to. It's something that you have to judge. Or if it's something that automatic that's automatically. Detected oror. I guess it could be both with an. In that case. If it's automatic, of course. If it's, it's about the precision of your instruments, and of course one important thing in many in vitro setting is that everything is continuously checked up and calibrated so that you know that you're measuring your measurements are correct, I guess. But I'm sure that others here have more practical experience with those needs.

Paul Whaley
Getting a couple of nods here. I've put some of the sport. I can put up the things that came up frequently, so it did were things like detection of the show exposure level. Detection of outcome. East of the park, for comparison groups to make sure the outcomes detectors. I think this is where the controls come in. And then the masking of investigators said the outcome assessors specifically, particularly when they're making subjective judgments so. Knowledge of the exposure level or group could influence their judgement as to how many cells they counting or you know the colour of something, or the arrangement of things depending on whatever it is they're looking at. Maybe so talking a bit about detection of outcome then, because I think certain limits of detection came up quite a lot in our. Included assessment tools. For example, if you got. The presence.

Participant 5
I don't know if it is. If it is within this detection bias, but for instance working with nanomaterials, there is a really big issue with interference with recorded values. So none of materials can interfere with the absorbance and fluorescence values. I don't know if that is something that would lie underneath this because then you could get biassed throughout your exposures. Ohh outcome.

Paul Whaley
So it does sound like detection because it would be distorting your processes for determining recorded values, because if you've recording fluorescence and the. That material is impeding that.

Participant 5
Yeah.

Paul Whaley
Then you could be under under recording, right?

Participant 5
So in the Nano Nano field this has become, you know, focus area really during the later years because a lot of, for instance, viability stains are affected by nanomaterials and also allies us non materials combined. And the protein so, so this can give really a bias? But yeah, it was a suggestion, for example.

Paul Whaley
Yep. Thank you.

Participant 2
Is it? Is it definitely a good suggestion? And if I may add? When you're doing. Immunocytochemistry, for example, and you want to use multiple primary antibodies or even an antibody or a couple to three antibodies bind with that PPI or apps or whatever. It's always very important to look at the overlapping of the fluorophores. So to avoid this kind of cross signal among the different channels among the different protocols like you're measuring, it's very easy there to. Mess up with data. Both. If you're expecting expecting the southern counting the cells manually, or if you're counting them with an high content imaging platform. So in both cases this is something really concerning and. Richard read. Definitely pay attention there. You may count ostracised for neurons and just because the photos are very close to each other or have double, double stained cells then look like double stains. But definitely they're not the same type of cells. So these are all things we consider there in essence in general, whatever technique, chemistry, history, chemistry, whatever it is.

Paul Whaley
Maybe. Maybe the team's volunteered here.

Participant 3
Ohh yeah. No, but then we are working with the primary selves and that can affect at least the cell counting of the cells because you have some, yeah. To all tissue remnants that there will affect the accounting..

Paul Whaley
Participant 4, did you want to add anything scares you? Not on camera. So I don't want to forget you.

Participant 4
Yeah. No, I was just thinking really it's the standardisation of protocols. I think about my own lab and like as we were talking about C and all these things like I'm always like paranoid of that happening like someone biasedly skewing towards this compound being, yeah inhibiting of differentiation. So I tend to look over the IC first like OK, I see this general pattern before they bring me images and I'm like alright, I want three to four images per well, but it's really this, it's the standard. Position of trying to like implement in your lab of how to prevent bias sometimes I also do two people working on the same project and you know they have different arms of it and then they end up with the same outcome in the end. But I mean yeah.

Paul Whaley
Hmm. Ohh good. Thank you. So tricky ones are lots of tricky ones there for us so. Detection of confounders came up. This may have more to do with primary cultures perhaps than like kind of. Kind of experimental and betray ones we have issues around timing of exposure. Ohh so time point window exposed, duration of exposure, latency periods. These were concepts that came from a lot but weren't obviously very consistently used. Maybe it's worth talking a little about about timing of exposure at this point, in fact. So how much it can matter when the exposures applied and how long for I suppose in relation to maybe sell? Maturity or the how long you expect the culture to kind of be giving valuable data? Or do you have any sort of thoughts or comments on these sorts of things?

Participant 2
If I may start what time you love exposure depends really on the study design. What's your intended goals and what what? What is your research question? So and also the biological model, does it has to be differentiated for long period of time or only one week is enough. So there are many, many aspects here to consider. But generally speaking, um, I think it's important to set up some clear quality controls. So for instance. If if you if you're expecting to depreciate the stem cell into a newer ones or whatever and apathy sites etcetera, you need to have some clear QC to define how the cells should be at the end of the cell of the experiment. If the cell should be completely differentiated by the end, or if you're going to treat the cells during the differentiation process. And if you're planning to refresh the treatment and the medium. Again, probably understanding the viability, the, say, the Emmy life of the compound, does it get graded fast? Does it get metabolised by the cells? If you then refresh twice a week or three times a week? Um, how does this? How do the cell behave? Many aspects to consider, I think. I don't know if I'm replying to this question, but that's pretty much what I would think about.

Paul Whaley
Complicated. Don't have any thoughts they'd like to add to that. So it could also talk about timing of measurement of outcome as well and how that can distort. The. Results of the study. Because it's might be, yeah, the role moment for the outcome measure that's been chosen. Participant 4 Have any thoughts on that?

Participant 4
Yeah. So, I mean, it's kind of agreeing back what was just said with this. I work with embryonic stem cells and I'm always trying to assess the developmental toxic effect of a chemical and so. I wanna see how it's the chemicals affecting the whole differentiation process. So I start exposure on you know day zero of differentiation and we don't actually assess until day 20 of osteoblast differentiation. Um, but we've done studies looking at different time point Windows, so we've seen that. Yes, we have an effect on differentiation on day 20. But if I was just too exposed from day zero to day seven, I could see the same phenotypic effect at the end. Whereas if I said well, I just wanna see what the effect of the chemical from Day 7 to day 20. You really have to have quality controls set up and ask the questions well every time. Affect the whole process are requesting if there's a time point and differentiation that is more susceptible to the chemical. So that is a really big one and it needs to be like laid out early on in the process and understand what your implants are going to be to be able to measure and interpret that data.

Paul Whaley
OK, thank you OKI need some nods. Something probably if.

Participant 3
Yeah, I'm running a primary school assessment and it also can depends on how many people you have helping harvesting the cells. That would of course affect depends on how fast they are collecting the cells and if you manage to hit the right time to harvest themselves.

Paul Whaley
So that's an additional point. I don't think we've heard that one before. That's very good. Something else that's come up was quite challenging I think. So we had a long discussion about this. One of the other focus groups is this idea of marker cut off points. Which I think her thresholds for. Determining whether or not the outcome is present or not. And that there's a related issue here. By converting continuous data into categorical data or something. Do you have any thoughts on what Markov points kind of actually means to you? Because it kind of is very contracted phrase and we didn't really understand what it meant in the tools that were abstracting from. Would you speculate as to what the? People who propose this criteria might have been thinking. Maybe. Participant 1, I'll put you on the spot first this time.

Participant 1
I was thinking about like thyroid hormone type of things and that like you know, in the clinic there's a biological. Threshold right where at at this threshold you have, you know, hypo or hyperthyroidism or whatever I'm trying to think of that though in the context of cells. And all I can think of is like. If there's like a. You have a plate reader. At some point. You know your positive control will. It'll be close to that. I. I mean it's it's kind of an odd question. It seems a little subjective. Baby.

Participant 2
Yeah, I I I fully agree. And if I may add to this, it it really depends on how well you know your test system, so. Um, for instance. One of the thing we were looking at when doing this development in your talk studies was multi electrode array analysis and therefore electrical activity. If the culture at that specific time point time of differentiation was not. You know producing certain number of spikes per second and. These cut off right? So ideally it should have been more than a certain threshold or equal to that threshold, but if it was too low it was simply discarded from the analysis because whatever we were going to measure was not probably very reliable. So it's very important to know what you're working with and set up very good quality control criteria to accept a given test system for study to understand if that biological replicate makes sense or something went wrong during the process and the cells are screwed up. So. These are things to look at, I think before, especially in control wells, I mean those are supposed to respond normally and you should know what you're dealing with.

Paul Whaley
Hmm. But is there anyway in which the the the choice of what you're kind of marker cut off is? Could distort the results were studied like if you said it higher or lower. They're saying you're expecting a certain number of like peaks or something in a given period of time. Like that decision that you know, it's gonna be more than five or it's gonna be more than three or it's gonna be more than 10. I know it's not more than 10 say then it's a negative and if it's more than 10 then it's a positive like you said at 9:00. Instead, that could change the. To that, distort the results of the study in some way.

Participant 2
If it is very, in my opinion, if it is very close to what you would expect to see a bit lower than a given threshold, then it's probably acceptable. But if it's definitely and then again how to decide how big the difference should be, right? How? What is really something that can be accepted or discarded? This is something that is based on experience and. While looking for example at percentage of neurons, we were really counting the cells in the control and if they were, I don't know, 70% neurons and 30% ostracises, then the culture was pretty much normal. But if we were observing something like 20% neurons or even 45% urines, then it was a bit questionable. Something went wrong in the process and talking about, you know staining and for the characterization of the cells before the experiment, before the study or thinking about the electrical activities. Said before. So these are all things that comes with.

Paul Whaley
Uh huh. So I've just had a slight interruption Internet there, so we'll return to Participant 2 in a moment. Participant 3, I saw you nodding quite.

Participant 2
You rinse with experience of the model with the.

Paul Whaley
Ohh. Sorry, Participant 2, I think you're into that. Just just stuttered a bit there so we missed the last 30 seconds or so.

Participant 2
Yeah. I'm sorry. Can you hear me now?

Paul Whaley
We can hear you now.

Participant 2
Yeah. Yeah. So, uh, basically in a nutshell, it's very important to fully characterise what you're working with. That's pretty much it. And based on this experience, this historical data, then you can understand if a marker. Uh, I don't know. Whatever marker, whatever endpoint you're going to measure is reliably measured, and whatever detection you're going to do is reliable or should be discarded. I think it's really dependent. It really depends on your experience with that model. With that test system.

Paul Whaley
Umm. Thank you.

0:53:55.240 --> 0:53:56.990
Participant 6
No, I I I just it just it just came to my mind that you also. Usually to some extent include you know reference materials, so positive controls and that help you understand. What you're, I mean, it could be historical data as well as X just said, but did sometimes you can. I mean often you include reference materials as well. That tells you what kind or that helps you determine what you're expecting to see.

Paul Whaley
Hmm.

Participant 2
No.

Participant 6
I'm not sure if that relates to this, but just came to my mind that.

Participant 2
I agree, I agree. Ideally, ideally we should use a reference material. This is not done every time, that's the problem, but it should be done. For instance, we were using a very the you know when you work with with development on your toxicant. These are so-called dirty compounds. Sometimes they have multiple effects on your cells, so they may trigger different. They may perturb different endpoints at the same time. So, but if you're free instance, measuring your right outgrowth and stuff like this, um and possible effects on that specific and point, it would be really good too to use a compound clean compound that you know may preserve that specific endpoint, and therefore you may then compare whatever data you you're analysing with your reference compound. This is done in validation studies to be honest, but it's not regularly done.

Paul Whaley
Interesting. Thank you very much. Participant 3. Do you have anything you want to add to this discussion? So you're nodding a bit like you might want to say something.

Participant 3
Ohh, I think everything has been discussed so.

Paul Whaley
Okay. So you both thank you. The only other thing I wanted to just raise quickly was the possibility of equipment issues additional to levels of detection that we might want to be aware of as we think about potential sources of bias in relation to detection of recorded variables. So issues around. Like training sets and things and. In vitro size has come up a few times in other focus groups, so just wondering what you might think about that, for example, so you've got equipment calibration issues. How the equipment is. I guess maybe this might relate more to image recognition tools with maybe that need training data to function properly. Again, ontology sure about this thing.

Participant 2
This was kind of commended earlier on, I think maybe by Participant 6, I'm not sure. But yeah, having basically performing calibration of instruments on a regular basis. It's clear here. Then it depends on the instrument of the, you know, the schedule for the, for the calibration on frequently should be done etcetera. But definitely that's something to control. From incubator to I don't know, laminar flow hood to play 3 doors to balances to whatever you can think about. Or even microscope and everything.

Participant 3
And pipetts.

Participant 2
And pipettes, of course, absolutely. Maybe one of the most important thing.

Paul Whaley
There are many ways in which so if if there are calibration issues, there are things that you would be looking out for in a study report that would allow you to identify where those might be the case. But it might have happened that that perhaps weren't correctly calibrated because it was Thursday and they get on Friday.

Participant 3
Yeah, you. If you making your your exposure chemicals or solution then of course it can affect your Sultan..

Paul Whaley
Hmm. But in terms of equipment calibration cause I know this is kind of a big deal and the the GLP stuff or side of things that it can. The guidance on these. How to make sure that you? Getting good data out of your system, but I'm just wondering when we see a kind of reported in like the conventional scientific literature. What sort of things we might look for to make sure that. We're confident that, say, calibration issues have been. Taken care of properly by the researchers in some way? Maybe.

Participant 6
Honestly, I think that this is kind of difficult to detect. I mean, unless the data looks completely bonkers, but then it probably would. Didn't get published anyway. Or hopefully we'll get a course in the review process, but I mean, it's rarely if ever reported. You know, in the in a in a academic research study that.

Participant 2
It's not true.

Participant 6
To my knowledge at least, you know that they report their calibration procedures. Yeah.

Participant 2
It's not reported because generally is not requested. That's the problem. So people just don't write things that are not supposed to indicate unless it's compulsory. That's good thing, but yeah. We presume that people take care of this properly, but clearly they this represented bias. I agree.

Participant 1
Only way of getting that information would be to request it to say you know, you know, even if it's just a brief description of what you're qcq for your equipment is.

Paul Whaley
Hmm. Okay, so thank you. Right. So next domain was an empty domain, so predictive model research bias was actually introduced. To discussion by the 1st Focus Group. Ohh so we have no correlating. Christ area and our system at all. Our definition of predictive model research bias is a bias specific to the design, conduct analysis, or reporting of research about predictive modelling. No examples at all, I'm afraid. For you to think about, but it does have something. I think analogist to diagnostic tests prognostic tests whereby you were using your in vitro system to make a prediction about something that would happen like in another system, right? So it may be an influential bias. Um, it might relate to maybe qsar type analysis. But we're not exactly sure how to think about this. So do you have any immediate thoughts or intuition what this might mean and how it might understand it for invitro contexts? There really are no wrong suggestions here at all, because we just want to kind of get a sense of what people's intuitions are, and that will help us make sense of how to. Construct our tool and think about what bus might be relevant here.

Participant 1
I understand what you're asking. You mean like a bias if from. And extrapolating like. In vitro results to like. A human or a mouse or something like that. Opening.

Paul Whaley
We're not sure what was meant here to be honest, so your guess is as good as mine. This is just like a sentence and like this is the sentence we have. So your reactions to the sentence are going to be very helpful to us. Whatever happens. So.

Participant 4
Yeah, I'm also thinking.

Participant 6
To me it to me it sounds like, yeah me. It sounds like it's a non testing. I mean an an on test method so like queues are like you said, like in silico and but then I guess it wouldn't be relevant to include in your tool. It's about if it's not about the cell based type testing.

Participant 3
Isn't it the in vitro in vivo extrapolation? That you have data that you produce in metro and then you ohh. But yeah. Extrapolate the data cannot to concentration in vivo.

Participant 6
But that would be a specific. Yeah. No, I can't really wrap my head around it. I'll be quiet.

Participant 2
Well.

Participant 4
Thank you.

Paul Whaley
No, no, this is my thing. Had around as important. So Participant 4, I think you are up next.

Participant 4
Yeah, I was just thinking maybe the wrong cell type. You know, sometimes we see studies where they're saying, you know, ohh it's a predictive model of a lung tissue or something. But then you're using a transformed cell that could be, you know, cancerous like, is that really a good predictive model? But I'm yeah, I don't know. Like all like if he does an inappropriate cell type for the study, like the setup.

Paul Whaley
Hmm.

Participant 2
Yeah, we have to do.

Participant 1
Yeah, I I think. Sorry, I kinda to go along with what Participant 4 was saying. Like for example I've. Seen in places where people will take like Chinese hamster ovary cells and try to use those as reproductive models and that's the that's like. That's not what you want to use that for.

Participant 4
Alright.

Participant 6
No, no. I just also wanted to get back to what X said that sometimes people think that they're using one cell type, but it's actually not that. The pricing get better. Yeah, yeah.

Participant 2
No, if I if I just can add to this discussion, I think it's important to always consider. Ppk model here. That can allow you to compare your indietro. Those is whatever you have tested the effect it oxycodone mamic toxicokinetics you have observed in your invitro with what can be observed in developed. So we did actually some comparative assessment and it came out for example that our invitro. Concentrations were actually much higher several orders of magnitude higher than whatever exposure was estimated in the epidemiological studies. To use that specific developmental neurotoxic effect. So I think this tool, so pbpk models whatever can be used and there are quite several models nowadays that are available will help to possibly sort out this issue.

Paul Whaley
Hmm.

Participant 3
Also depends on how you run your cells. If if it's two-dimensional, or if it's three dimensionally, they will give different results as well.

Participant 2
Absolutely, yeah.

Paul Whaley
OK. Good. OK. Thank you very much. Well, got close to figuring this one out, so next one slightly easier. Have 15 criteria in this domain of reporting bias. So we defined reporting bias as a biassed due to distortions and the selection of or representation of information and study results or research findings. So this would be about selection. Of the. Data that is presented or it can be about how the data that is presented is. A representative, so we call it something like spin in English. So you might be kind of. In the way that you do it, make it sound a bit fancier than actually is right. So. Any immediate thoughts or reactions and how this can manifest in intro studies?

Participant 2
But simply, I mean, generally speaking, I think oftentimes people tend to select the data that are more convenient to the study so that there are completely contradictory to the general, you know, storytelling or whatever you're trying to demonstrate are discarded or. For example, if you measure multiple genes and within a set of gene of genes, some are like. Completely and nonsense. Then they get simply discarded because they don't support your original hypothesis. This is typically done when we think about selection selection of data.

Paul Whaley
Hmm.

Participant 2
And with regards to representation, uh well, data may be represented. I'm thinking about linear scale logarithmic scales. You may give the impression that differences are much bigger when you're present them in a logarithmic scale, or the other way around. So these are all things that just come to my mind quickly.

Paul Whaley
Hmm.

Participant 5
Yeah, I also ohh sorry. No, I also thought that there is clearly under reporting of negative data or no effect data. So that could really be I I think that is a big bias in the available data sets that are out there. So if you get a lot of data that is not really giving any effect and you get one that have some effect, then you will really play up the one that have the effect and the negative or normal effect data would kind of be lost or not reported because it simply difficult to publish those type of data.

Paul Whaley
Umm.

Participant 1
Kind of along that same line, selecting maybe images that are. Prettier than maybe the rest of your images and I'm. I'm thinking about like. And I grew up in a molecular signalling lab, and so we were very particular about like Western blots and how they looked. And I remember RPI, we could tell when they were publications that were comparing different. Proteins and were measuring against others, you know, even housekeeping genes that we could tell when it wasn't the same experiment because of the shape of, you know, the the image we could tell when it was the same while and when it was. A A different well because of the shape of the blot. And so. And I think that comes from selecting images that are prettier because it's, you know, more convenient or, you know, you wanna have a pretty picture in your publication..

Paul Whaley
Hmm.

Participant 6
I I also maybe think of now with all these. New methodologies coming out and generating lots of data and on mixed data and all these types of analysis. Halfway analysis and yeah, gene expression analysis and you can really. I I I don't have a lot of experience with that myself, but understanding from hearing colleagues for that you can really, I mean you could when you do these types of analysis, you really have to select. You know what to look at and from which which angle to look at all of this to. So it's, it seems to me that it can be quite subjective in a way. Um, how you choose to. Analyse the data. Um with shop. Which software you use? And then you know how you select which genes you you look at them so and you can really generate different pictures. And of course it's it's great because you. And you can really use your expertise to maybe pinpoint the types of effects that you're looking for and but of course. Then. Theoretically you can also. Sort of paint the picture that you want to show as well. I guess what I mean within limits, but to me that seems as an assessor. I mean I I get a little bit. Care for if and when I see these big data sets and deep analysis and you know, it's just like you put big data in one end and then you get like a Sarah answer at the other end and you don't understand that black box. And what goes into it and the assumptions and principles that go into it, then you you're not in a good spot to really assess their question the the outcome.

Paul Whaley
Hmm. OK, alright. Don't have any thoughts in addition to that.

Participant 3
It depends on the person that's analysing as well that data for the big data sets..

Paul Whaley
Hmm.

Participant 2
And another thing to consider is who is actually performing the analysis. The statistical analysis. For example, if it is the same person who did experiment. Or you could someone else. There may be a biostatistician in the end dealing with this big large data sets and. Most likely I have impression that when this is performed, when the analysis performed by biostatistician, it's kind of more robust and reliable. The tendency towards bias is less prominent probably than someone you know performing an analysis and then analysing the data set himself or herself. So yeah, this is for instance, in validation studies. This is mandatory. So you really need to have a biostatisticians dedicated. Who will perform these type of quantifications analysis and statistical assessments?

Paul Whaley
Hmm. Yes. So the issue that came up comes up repeatedly in the included tools relates to having a choice of exposure levels and then picking and choosing between them a choice of outcomes and picking and choosing between them. Choice of analysis and making it choosing between them. I think fundamentally so sounds like we've covered that territory quite well. One of the things that. Is maybe more difficult to. Think about and was kind of harder to pin down out of the included tools that we had in our literature review was selective emphasis on post hoc analysis. So post hoc analysis is you've already had sides of the data and then you figure out how to make sense of it. So the concern is that if you haven't. Pre specified the analysis before you look at the data. You can confit the analysis around the story you're trying to tell and I think we've touched on this a little bit, but I'm just wondering what role. Um, maybe pre specified analysis plans and things have in in vitro studies. Your experience of this how much you think it matters that you know, maybe what, maybe exploratory analysis of presenters, if they're the only analysis that did and these sorts of things.

Participant 2
Generally speaking, in many publications I see nowadays people specifying if they have assessed normality of distribution of data or and with which specific software or programme. So these are things to look at, probably to really when you consider a bunch of data or a group of data, understanding if there is a normal distribution or not. And selecting the most appropriate. Ohh statistical. Statistical methods and post Doc start test to use will will be consequent to this. Nowadays, most of the reviewers of your review publications, many of them at least not all of them, but many of them tend to ask about these aspects, so that's probably something to consider into this discussion.

Participant 6
Yeah. No, I don't. Maybe coming back again to what I rambled on previously, but when you have these mean. I get the impression like a lot of data or a lot of research, they is. It's quite exploratory like you, you expose yourself and you're getting all these gene expressions and you're exploring which types of pathways are are activated or. And. So then it's I guess it can be kind of hard to decide in advance exactly exactly which types of analysis or or. The the the details of your analysis in advance if if your research is kind of exploratory then maybe you adjust then maybe you have to have an idea of the data that you have before you start playing around with it. So I'm just assuming that that those situations also. Except when it can be quite hard to define in advance exactly the types of analysis you're gonna do.

Paul Whaley
See, I think the concern might be here is that it's it's about selectivity of emphasis. So you've got the primary analysis that was done and then that's kind of just sort of pushed to the background a bit because the post hoc analysis is more exciting, right? It may be in some way.

Participant 2
He's exciting. Yeah. Because you can draw some biological conclusions by looking at those, you know, getting really differences among different groups and whatever. Yeah, there is an emphasis in post doc analysis. Generally speaking, most of the data India and you look at our post DOC analysis data.

Paul Whaley
M. So just coming to the end of the 90 minutes, just Participant 4, do you have anything you just want to add here just to give you the opportunity to speak maybe last word, huh?

Participant 4
I think everyone pretty much said it, but I mean we all like to say that's significant or not. But like in my case, we have a stat score. So when it's complex, hopefully others are doing the same and going to the you know the experts to actually analyse their you know provide that analysis in the end. So you're not you know making it happen.

Participant 2
That's the ideal, yeah, migrant situation.

Paul Whaley
Very good. OK, super. Well, thank you so much. We'll be reconvening tomorrow. Same time to start with early study termination bias. Again, another domain. I will save that for tomorrow, so I'll just.
Thank you all very much for very energetic and incredibly helpful discussion, so hugely appreciate it.

### Focus group 3, meeting 2

Paul Whaley
Right. So we know we're recording. Did that already. We can head straight into selection bias today. Tricky one, we had 20 criteria. That were put forward in our 7273. Excuse me. Included. Assessment tools. Selection. Boss. Excuse me. I'm just gonna cough for a bit of. Got a frog in my throat. Rock out. Selection box we have defined as a bass resulting from methods used to select subjects or data factors that influence initial study participation or differences between these study sample and the population of interest, but quite a complex issue in risk of bias assessment. And it's to do with how you can have distortion in your study results from how you choose. Who or what gets into your study in the first place? Factors that affects you know, you might choose a group of people and then or animals or whatever, and then for whatever reason, only a subset of those get into your study and they might have some unique characteristics compared to the ones that didn't. So you don't have like comparisons. Or you can have difference in the study sample. So and the population of interest. So you might have issues with how the. The group thought the the the sample you've got. Isn't reflective of the sample that you're trying to be representative of. That makes sense, so used obviously a lot in. Human trials and animal studies. It may be that if you're doing a primary cell cultures, that selection can be an issue. Um, it might be that there are ways of choosing which they plates or whatever are included in your experiment could also be a factor. Would anyone like to start off with any comments or thoughts? Clarifications around this type of bias and how it might apply in in vitro contacts?

Participant 2
Can I just start with something very simple that comes to my mind? I think all this discussion is about the concepts of fit for purpose, especially within vitro methods. Not every test system is suitable for everything. So, uh, you know, I'll complete characterization of the model test system. Of course is highly recommended at the beginning of study design. And given model, even the same model grown in 2D monolayer or in 3D, maybe suitable for different purposes. For instance, in the context of developments in your toxicity, our neuronal model grown into D was suitable for a given methodology and analytical tool, which is for example the module lecture array, which is basically a they dimensional surface with microelectrode spotted on the surface. On the other hand, the 3D was less suitable for this, even though the 3D model was generally speaking more mature, performing well. Even oligodendrocytes were popping in. So it's all about deciding what is or having really clear in mind what is the fit for purpose, what the model is suitable for, basically. So that's a nutshell my. First thought.

Participant 3
Seconds that comment on what we are doing in overlap. May you sing. Salmon primary hypothesis and may be collecting the cells from clonal fish lines mail line and we do that to reduce the. Or to have to eliminate the females because. Of course, it's also depends on the money. So usually we have, uh, six animals. We isolate cells from and to collect 6 males if course toxicity depends on sex, so we want the same sex and usually by collecting the mails. And the to kind of don't have to kill all these females. We are actually not using this clone line. That's only male and we don't have to think about that. But of course that just only reflects the mails. But then we kinda in in the in vivo trials, kinda, yeah, I just for the female that was ex relation with female male effects.

Paul Whaley
So who else might want to respond to that? His bought might happen. Typically if we're looking at.
Animal or human studies is that. Ohh, let's see. You'd have some eligibility criteria for entry into the study. Um, and it may be that the way that the eligibility criteria applied results in selection pressures on those the entry or not by a classic that has happened in. Let me see this. Be willing. Yes. So I think one of the questions I'm just going to put up some. Examples so I don't have to make things up and then get them. So there are potentially issues with cell line authentication. I think with in vitro studies specifically. So for whatever reason, you've entered sales into your study that aren't the cells you thought they were. Uh, I think so. Line contamination comes in, and that hasn't been heavily discussed. I think in the previous focus groups would be nice to hear what's I like contamination means to you and how you control it and how you get signs. Maybe things not being right there. Um, it's possible that conditions of cultivation or maintenance can introduce selection issues. Uh, and then it's possible that you could exclude units from analysis due to missing data, so you end up with again another way of getting differences between the. But does it that's influencing not there initial study participation? The question that's what that looks to me like. It might actually be in attrition issue. Ignore that one. That may be an error with hindsight. Yeah, I think particularly so on authentication and so on, contamination would be interesting to hear if you have any thoughts or comments about.

Participant 2
By contamination we mean any contamination could be mycoplasma, could be even cross contamination between two different lines, for example. So we're talking about contamination as all in general.

Paul Whaley
Was though the criteria was contamination in general, but we weren't necessarily sure of what the specifics of contamination might be and which ones we should be more worried about, which ones we should be less worried about.

Participant 2
Well, I mean this is obvious, but micro plasma contamination is probably the nasty contamination because it's a really, you know, something that you don't notice even, but you may get different responses in your biological assay or whatever exposure. Bull, etcetera. So you might really. Have completely different results, so generally speaking, in a GLP lab this is actually a nicely tackled you just do a micro plasma test once every two weeks. Generally then, it depends on the rules, but that's what we try to avoid. And for Celina, 10, dictation once a year, we were kind of. Cross checking all the cell models in the lab to really see what we were dealing with if it was. Exactly what you know we were using since the beginning years before or if something happens along the way, some mutations occurred, etcetera. So that's something we were doing on a regular basis, both these two bullet points. Yeah. I don't know what else to add.

Participant 4
Yeah, I would just add with the cell line contamination as well, sometimes it's making sure you're not working with two cell lines at the same time, right? Or like pretty big and the lava about don't take your H nines and your I, PS or we don't mix you know the mouse cell lines and the human cell lines and the same hoods, they're all in separate incubators. So I think about that too is selling contamination. But really the labs should be set up with protocols where you're authenticating yourselves every so often, checking mycoplasma and making sure. You're working properly with yourselves.

Paul Whaley
There any circumstances in which like. If we're thinking about. Things being analogous to the eligibility criteria study participants, whereby you could be doing some sort of inspection or like analysis of the, you know, we've got a culture, you cheque if it's appropriate for the study like is it, has it grown properly, do you have the if it's a 3D cultures that have theoretical says that you're looking for and then you have some criteria for whether or not you say okay. This culture is failed we get rid of this 145. Which is there. Fine. They go into the study like, is this ever like a? Many considerations or thoughts relating to that.

Participant 2
Isn't this just common practise? I mean if you have 4 cell model for example and two or three are. You know. Performing well and one for some reason does not get differentiated properly or it's more tricky to culture than if two or three are enough or even one. Most of the time. Then you just get along with that and you discard the one that is not working. This is clearly potentially biassed, because of course you should include as many samples biological samples as possible, but in real scenarios this is actually what happens. This happened. This is very frequent. This is actually goes back in time when I was still in academia at that time we were collecting biopsies of brain tumours and we were dealing with many, many, many biopsies and clearly some of them were not, you know, for viability reasons, even performing well, some other were really good. Ohh and um, you know, growing cancers themselves was pretty easy from growing from them from others was pretty tricky, so. When we were publishing a paper then we were, you know, selecting the seven most suitable, more interesting, most interesting cell cultures, and discarding the other three or four. So yeah It's a bias, but in the end it's common practise, I would say.

Paul Whaley
So this kind of does touch a little bit on a couple of things that came up in the included tools that we weren't quite sure about, which relate to cell density. So cell density issues and then the representativeness of the sample. So I don't know. If you want to talk about cell density at all like I think there was some. The tools echoed some concern that you know if your cells are too tightly packed, it's in some way not representative of. The system is actually trying to model in some way or not a good model if they're not packed densely, packed enough, that can be an issue as well for other models. To have any thoughts on this?

Participant 2
Can I speak again?

0:17:25.960 --> 0:17:29.530
Paul Whaley
It kinda yet no less jumping forward by will put someone on the spot in a second.

Participant 2
Yeah. Yeah. No, I don't want to take the words all the time, but no, just a quick thought that cell density matters a lot, because even the responsiveness to compounds changes dramatically depending on. How dense are your cells or even grow? You know, passing from a through 2D to a 3D which is definitely more than some packed. The sensitivity to chemicals is completely different, so this is really a big deal in my opinion.

Participant 3
The other. I mean, with our primary source, they really like to be very dense. So yeah, you should try optimise your system.

Paul Whaley
Yeah, you're nodding.

Participant 5
Yeah, the lime agreeing. So for some assets you need cells that are in proliferating phase and then you they can't be too, too much of them or to dance and for other essays, maybe you need them to be really dense. So I'm I'm. I don't have anymore to add them just agreed with the previous speakers.

Paul Whaley
And then maybe. Ohh no, if you have any thoughts yet so I'll have from you yet.

Participant 6
No, and I think the the people with the more practical experience can probably answer this better, but I can just agree with what's been said from my understanding and also with the most important thing is that there is an understanding of the cell system that you're working with. And so that you're working with it properly and that you're you're using the cell density that's. Ohh, that's appropriate for that model, so that should be clear I mean. From the way that the. The study has reported.

Paul Whaley
Okay. Then Participant 1, do you have any final thoughts on this?

Participant 1
Yeah. You think just to that, I guess just to add to that last comment, I think cell density. Well, you know, depending on what your outcome you're trying to measure is is something that should probably be reported depending on your cell type, because they're gonna, it's gonna affect if you're looking at mechanisms, it's gonna affect the different different signalling of the soul. And so reporting that cell density for your specific cell type is probably really important.

Paul Whaley
Whatever sleep, I thank you. Works. Gonna move on to the next one. This is another empty domain which is quite challenging to think through cause it's important in a lot of. They're very different research designs, but it was it. Nothing came up. In the included tools that spoke directly to this issue. So early study termination bias we define as a bias due to the decision to end the study earlier than planned.
The other hair basically is that. Sometimes you see in trials that you observing are maybe if there's a medical intervention, you're observing A beneficial effect, and then people terminate the trial early. To maintain the appearance of beneficial effect because of favourable results, then they might be worried if they carry on collecting more data that that banner should affect becomes diluted. The alternative might be that you continue studying something longer than originally planned, in the hope that you'll start seeing significance where maybe you couldn't see it before. Either way, maybe it's a break of protocol, but we didn't see any specific examples and I was just wondering if you think there might be ways in which in vitro studies could potentially be vulnerable to. Maybe somewhat arbitrary decisions about. Stopping or continuing. To run an experiment. And you get that changes the significance of the results.

Participant 6
Maybe I can start them with my limited experience, but just from what I'm picking up guys and is that I think that in the case of in vitro studies, you usually test these things like you explore the timing, that's at least what I hear from my colleagues. You know, we try it at 2 hours. We try it at six hours. We try it at 48 hours to try to find what where the optimal time point is to. Yet the most sensitive results out of the model...

Paul Whaley
So that's seventy thoughts responses.

Participant 4
Yeah, I'm thinking like ohh we're initially doing like concentration response curves like say if we didn't hit an icy 50, it's like full stop it and we'll just redo it, change the range in the concentrations that we're testing cause we wanna make sure we're at least passing that I C 51 more first trying to determine what concentration so I stay there and also if you're control's not differentiating as you expected to during you know whatever experiments going on or gonna cancel it. Is it the control's not doing what it's supposed to? How could you really? We say, OK, this concentration inhibited differentiation or led to the upregulation. If you're control's not, you know, properly doing what it should so.

Paul Whaley
So maybe this is a totally stupid question, right? But. Is there the possibility that if if like that you could maybe over optimise an in vitro? Experimental or test setup whereby you kind of, you know, practise it 50 times. You know highly optimised the amount of time the exposure is for to generate the most kind of like best looking results, but it ends up being a bit like a like a statistical overfit issue where you've got an over trained model or something like is there. Is there a possibility in in vitro study design that you that even makes any sense that what I just said or would that just be? Figuring out how the test system works, you get confident in it, and then you run it.

Participant 6
What I'm I mean you want to have. A sensitive responsive. Test system. And there are many factors that go into this. I mean, the right concentrations, the right timing, the right cell type, the right conditions. I I'm not. I don't like and you always any results you get. You always have to. Interpret in the light of. You know the the test model and the condition that you have and what it means at the end of the day, we need to interpret. What it mean for human health in this instance, in this case, so I I don't. I don't really see the danger of making the system too responsive. I'm used to always have to take that into consider when you consideration when you interpret the results anyway. What does this mean for for human health? That mean it's worse. To me, it's worth to have an unresponsive system. That tends to miss. Things that you. Want to pick up?

Participant 2
They may add to this. There are a couple of examples that comes to my mind over early study termination bias. Want it's kind of technical, so let's pretend that you have a tool at late reader, or I think about the multilateral rabies was our nightmare, so it kind of. I don't know it it it did not work at a certain point in the machine was not working at all. But the study was basically kind of terminated. I mean, we've collected the M5 and everything was going OK, but for some concentrations and compounds were supposed to test a few times more just to be sure, because we had some dropped out of internal replicates, technical replicates. And so we wanted to add a bit more biological replicates, biological experiments. Um, But the machine was not working and then when it started working again for some reason the sensitivity of the machine was a bit different and we basically stopped the study there because okay we have enough and it's enough, why shall we complicate our life? Let's start with something brand new and don't keep pushing this direct here. This is just a stupid example that comes to my mind. But I think there may be a bias there. Yeah, why not? If you keep repeating the experiments several times. And the instruments get recalibrated. Refixed or whatever happens, you may hand up with different results and therefore you may end up excluding the newest set of data. That's one example. Another that comes to my mind goes back a bit in it's own time as just what I was referring before to the biopsies, so. We were collecting many biases. Biopsies twice or even once twice a week, and we could have continued the study for relatively indefinite period of time and continue gathering data. But at a certain point we decided to terminate the study. The data where enough we were able to publish and the variability of the data was going to increase by just adding more biopsies to the set of data. So this was more like. Biological variability among samples and when comparing 1 sample to the other. So there was more variability in terms of statistical significance, but clearly it can be a bias because if you keep adding you may have a large variety of different biological samples and tumours. In this case and. You might, we may have. We could have ended up with slightly different results and slightly different results interpretation. Does it make any sense?

Paul Whaley
Yes, I it more sense of what I was saying earlier, so. Cause I think one of the things that is of concern is that it's the decision to keep collecting more data or to stop collecting data based on the results that have already been observed, right? So if you look at the date you're like, oh, this is nice, clean data. I'm gonna stop there. Whereas if you had carried on, you'd be like, well, actually this. I'm now guessing quite noisy data and I'm less confident in it. And you you, then your determination of confidence isn't really to do with the experimental setup is do the amount of time you taking readings for and you decided to stop because it started looking like it was getting noisy when actually that might be useful information that would be informative for contextualising the findings or something or it would increase the variance or that could end up reducing, introducing enough noise that you end up deciding. Don't have significance or otherwise he might have done so.

Participant 2
Absolutely yes. And this actually applies even to systematic reviews. Considerations all many studies should be included up front. Where do you decide to stop? Why not included ten years more, five years less? Whatever the same story applies there that think the same concepts. Meta analysis and systematic reviews. That's what I'm referring to.

Paul Whaley. They have any thoughts in?

Participant 3
No, nothing to add.

Paul Whaley
So it does sound like it could be the case that you can have early or late termination. So this is kind of just important for us to understand who probably explore in more detail later. So we'll move next to another empty domain. Which is something called choice of question bias, which again was in our biassed domains, but didn't have any. Criteria in our included tools, which may mean it's not important, might mean that the tools just haven't covered this. So show us a question. Bias concerns a bias in research design in which the research question that the study is designed to answer is inappropriate for the context. Um, we have no examples, so we're just going to invite some open discussion. Largely around how. The. Yeah. The question is being asked is somehow contextually appropriate. So you'd end up in Ferring, something that was. False When maybe was true or something. And your experimental study. I might make another go first, because this is quite a systematic review type concept, right?

Participant 6
Yeah, I'm trying to think. I think so, but wouldn't that just generate like irrelevant results? Or you mean a situation where the you can the results can be used but your then your answering a different type of question really. But I don't really. I don't really understand it.

Paul Whaley
Well, we don't either, so that's everybody in the same boat.

Participant 6

Um, because if you. If you're interested in. Developmental neurotoxicity like Participant 2. But you're doing you're. Doing a test in. Uh, and I don't know, hypothesises looking at something else then the results you're gonna get, they're not gonna be. Relevant answering the question so.

Participant 2
But.

Participant 6
But I mean, I understand that that's that's an extreme example, maybe nonsense, but I guess you could do, you could have a SIM, I mean that you're just missing the target by a little bit and it's the the data still look relevant, but they're actually not. But I don't. Yeah, no, I don't. Maybe someone else has some good examples.

Paul Whaley
That's a tricky 1.

Participant 5
I was. I was just wondering, is it like? So you have your research question and then your research design. Maybe you don't take in all aspects that is relevant to your research question. So you're kind of biassed in what you actually analyse. Could that be?

Paul Whaley
It did pay. Do you want to?

Participant 5
I don't. I don't know if I understand the question, but I mean yeah, because.

Paul Whaley
With the youth say it's a very good thing you ask there if can you be more. Maybe be more specific in how you could anticipate something going wrong right? Cause we're thinking about internal literacy. So it is an error in results or interpretation. So it's just probably interpretational. So we can foresee the. You know, you might be drawing conclusions in the study about. Developments, neurotoxicity or something then do you end up saying ohh this is definitely looks pretty near a toxic in this regard but it may be that it's not as neurotoxic as you thought because of the way that the question in fact was asked or the way that the methods were fitted around the question or something, right?

Participant 5
Yeah. Maybe. Or maybe you like when you do your design for for to answer the study question, maybe your selective and you OK that analysis looks very difficult. So let's let's avoid doing that and then you draw a conclusion based on what you had. But actually maybe you didn't include other things that maybe should have been included to see the whole picture. I don't know. I'm just, yeah.

Paul Whaley
But we we don't know so.

Participant 2
Yeah, I can add to this just a quick comment. An example we were testing star scope to spike. On our 3D neuronal deal model. And some reviewers actually came to us saying well. There is no really, um, there are no proofs so far or reviews, but okay this is a brand new story, right? This is a better recent research field. So we we don't know exactly everything, but several people were questioning that there are no clear indications that source code to and COVID or whatever and despite proteins in particular may affect developmental the developing brain development. I mean the neuro developmental processes and therefore they were questioning the study itself. The question may be interesting. That it's not supported by the evidence so far. It was one possible element, of course. We commented on this in the discussion and we highlighted that this is all exploratory and. In the end, maybe just a silly study that goes nowhere, but actually it seems to be not the case at the moment, because there are some evidence of development neurotoxicity occurring so fine. Another thing that comes to my mind is when you test chemicals in general. And maybe they're not. I mean, either your model is not suitable for a given endpoint and they're therefore we go back to considerations about characterization how well characterised this your model. Is it suitable to pick that specific and point or not? Or or it could be that the chemicals you're testing are just not, um, developmentally you're toxicants or Apache toxicants or whatever. Maybe they target something else, maybe they target the lungs or the lever, I don't know. And you're testing them in a kind of wrong biological. In the wrong biological model. Um, so yeah, it's a very subjective thing and it depends on the context, on the study, etcetera, but these are the things that come to my mind.

Paul Whaley
Okay. Participant 1, do you have any thoughts on this cause? I appreciate it needs to leave fairly soon so.

Participant 1
Yeah, I think when X was speaking, I kind of was brought back to a time when I I was in the lab and you know there there are cells that are more sensitive to different toxicants than others. For example, I was working with both hepg 2 cells, which is a liver set, immortalised liver cell line and a primary muscle cell line and the muscle cell line was much more sensitive. I was also differentiating adipocytes and those were much more sensitive to arsenic than the hepatocytes where we used to joke. That we we could, you know dose these Hendry 2 cells with hydrochloric acid and they would say nothing's happening here. But so yeah, there's that sensitivity of of, of the of the, the model that you're using that should be taken into consideration.

Paul Whaley
I mean, it is a difficult one because it does sound because in French because it's influential is difficult to see in terms of internal body. So in I think in a systematic review you would look at some models and just be like well, this isn't relevant to the question we're asking and leave it to one side, but within the confines of a single study in the conclusions of the being drawn, it might be that.
There's less support for the hypothesis than.

Participant 3
But what?

Paul Whaley
The author's think because of that internal structure of the study, and we're just trying to sort of tease some of the stuff apart. So these comments were incredibly helpful. Thank you, because I don't have any job.

Participant 6
Can I? Can I just make another comment there for you and maybe this is beside the point a little bit, but it just came to mind because this sensitivity of the model and the sensitivity of the test is something that we. Often try to capture and and really evaluate and I'm not sure how if that comes in in any of the other biassed domains. Or maybe this is the domain where that is captured.
Because I think it's a very important aspect to be to be able to capture the sensitivity of of the model. Do I mean will you capture the effect? Are you using the right model to study the effects that you're interested in? Will you capture those effects? So if that is not covered in any of the other domains, maybe this is the domain where it's captured.

Paul Whaley
It does have that feel to it, so there's there's. Yeah, it's on a prejudgment thing, but obviously we've. Been paying calculation to the sensitivity guidance quite a lot of that looks like it fits quite well under aspects of internal internal nudity. Some of it seems to relate to accidental Felicity, but then there are some, there's some overlapping part where this might be as. Get get some of those things.

Participant 3
Can I just?

Participant 5
I also yeah, sorry. you start.

Participant 3
Can I just add that when you? Ohh you things asel model. You should also consider how well the medium covers the nutritional value for the cells. That would of course affect the whole their respond to a toxin. If you kind of use only FBS. If a 10% FPS and the cells OK, but if you using only like 2% then the the maybe the cells are not doing that well and maybe get more sensitive for the toxins.

Paul Whaley
Really.

Participant 5
Yeah, I was just going to say that. Um, yeah, we have been doing a systematic review looking at. Some nano particles and there yeah, the effect of course, as a carcinogen. And in this we used the talks art tool for. For the quality assessment. And in this tool there is a question relating to if the research design is appropriate for the research question. So in this one we actually looked at like we said, the sensitivity of the the model. If it is, it's a good fit of the model. But also if the concentrations are appropriate. Or in the case of the nanomaterials, if they have a good characterization of the the chemical that they are using, and if that how they are testing the chemical and the characterization, is it appropriate. So I think this is very. It's kind of complex thing if you really think about is the research design appropriate for the research question? It I think it's kind of a multilayer type of thing. So yeah, so because it's also can be like is the the time points you are using. Is that appropriate to answer the research question? Because if you are looking at inflammation and chronic inflammation is important for for development of cancer. But many studies in vitro studies have very acute inflammation. So 24 hours is that appropriate then for answering the research question. So I think, yeah, I just it's come to my mind suddenly, yeah.

Participant 2
And to add to this, also the metabolism is a highly important for of course, for instance, you want to assess the effect of a chemical, but in the end we know or you know that the. It's actually the metabolites of the chemical uh. There is the most toxic. Um. You should for instance understand if your modal expressed phase one or phase two enzymes cytochrome P, whatever 450 etcetera or whatever other enzymes that are involved. And for a pathside, this is something kind of more obvious, because this is something that all people working with the patrol sites and doing pathetic city studies course characterise their model for this. But even working with neurons, this is very relevant, of course, because some of your toxicants are just toxicants when they get metabolised. So that's another thing to keep in mind.

Paul Whaley
Okay. Sorry. Good point. Thank you.

0:42:12.40 --> 0:42:16.130
Participant 3
And then you actually need to run more advanced cell mobiles to actually pick up this.

0:42:19.0 --> 0:42:30.690
Participant 2
Yeah. I mean what we were doing in the past was really a detailed characterization of the model. Does it express this enzymes, not just the gene level, but protein level? Uh, and stuff like this. And then we were measuring really in the medium and in the cells, the metabolites. So um. Again, we were basically doing toxicokinetic studies to figure out if the the chemical, the branch will compound and the metabolites were present in medium and intracellularly and the fraction bound to the plastic of course. So that that was the only way to really be sure. That the cells were metabolically active, basically.

Paul Whaley
Or I will move us on text. This is very interesting discussion. You're very helpful for understanding how to deal with this, and we might revisit some of our criteria classifications on based on this as well as we might have. Like some opportunities for clarifying things, that's really good. So I'm going to go to the next one. So analysis bias, this was quite heavily. Populated domain of across the tools were abstracting criteria from. We have sort of roughly 32 unique criteria addressing analysis bias. Definition. A boss related to the analytic process applied to the data. So we view the experimental process obviously, as you've got things that happen that generate data. You've got data and then you analyse the data. So analysis biases any distortion in results due to analytic process. No analytic in the. Unless it chemistry sense. But in the statistical sense. So who would like to start with some comments on this? I think most testical models that potential misuse and in vitro study designs. I mean, that's the issues that came up quite a lot in the included tools. Included potential need to mask the. Person analysing the data to which exposure groups that. Ohh, experimental units were assigned the issues around correcting for or imputing missing data. A lot of stuff around data reduction and normalisation and standardisation and noise reduction. Techniques because I was understand that invite trade data could be quite. Ohh okay all safe and you need to do some stuff to impose some shape on it. And then issues around having prior knowledge of data before developing analysis plan. So you end up developing analysis plan based on what the data looks like which can lead to. Expected results being found more often, shall we say. That I have any thoughts or comments on any of this? But.

Participant 4
Yeah, I'm thinking of like what we did this already seek experiment. We let our grad student do the analysis of the pipeline first, and she did it and are using the decent too method. And then we had paid on at the university also to do it, you know, because the grad student learning. And so we left her do it and then we had the university do it and they used a completely different pipeline. Right. And the results of finding the differentially expressed genes. It was like. How do you choose cause I'm like ohh all the right hits that were coming up within one pipeline versus the other didn't have those same genes in there and it's like obviously you wanna go with what you're what supports you know you think is happening in that system. So I think that's one of the big ones. I think up there. And then we've talked about this before. As you know, when we remove outliers in an experiment, still you know to make the trend to keep that trend going and how that significance...

Participant 2
Yeah, I I I tend to agree with Participant 4. I think we covered some of these aspects yesterday in our discussion. Generally speaking, I think having a as I said yesterday, the same comment having a biostatistician they live with the data. It's better than having the same person doing everything. Well, this would be the most reliable and correct way in my mind to really avoid this type of analysis bias. Even the selection of the statistical. You know, statistical approach, ANOVA, whatever post doc with even deciding to show data is standard deviation and standard error means and stuff with normalising them rather than having them as well data. These are all things that should be decided in a concerted way with a biostatisticians. I think it's the best approach avoiding influencing him or her too much. That would be ideal.

Paul Whaley
Sort about imputed data. How often? Ohh from this necessary. What impact can it have on distorted results?

Participant 2
Computer data means anything. I mean, you may send your samples to an external company performing a full gene expression analysis or whatever. And then you get back to full set of data and partially analysed by them. Something like this, right? This is what you mean or.

Paul Whaley
So I am pizza taster. Usually it's usually when there's like Daisy's missing for whatever reason. So you might have a say I think a common thing is you got a range of detection of five. Let's just imagine a range selection for 5 to 20 for a given instrument. And do you know that? The some of the results that if you could detect wider than that, you'd see detection up to sort of 30 or 35 on different kit, right? So then if if you just take 20 as the maximum. Then you're going to be that. That will be a systematic underestimate of the true value. Now you can't just magic new kits out of thin air, so you have to do something with that kind of what's effectively missing data cause anything above 20 basically hasn't been measured. You know, it's more than 20. That's all you know. So when you do something to imbue data, so you have a truer result, right. But you're compensating for missing information in your in your system.

Participant 4
I guess it depends on what you're putting in. I'm thinking like with qPCR, if it comes back with no information on expression of genes, I would put 50 based on the number of cycles, right? So it gives you a zero. So I'm wondering if actually you reducing bias so so you're doing a cytotoxicity assay and everything is done, you would put zero if there's no reading right now, absorbance value. So I feel like. He would be putting in a number, yes, but hopefully you're putting it in a way where you're saying that. There's no representation there, but I mean I don't know. I feel like it could reduce size in that way. Unless you're falsifying data and then you know making it happen, that's a totally different..

Participant 6
Yeah. And and again, I don't have a lot of practical experience, but I'm, I'm I don't know how often this happens with in vitro tests that you have to. Input data. Because if things would also be below any detection limit, for example. What would you put then? Would you? But there or. Something there? Or would it just say like no? I don't know. Yeah.

Paul Whaley
What would seem to be a circumstance in which data would need to be imputed? It depends. I guess it depends a little bit in your statistical model and things it replying, but obviously there's a difference between the limit of detection and zero right, or the limit limited detection and the true amount of whatever's being expressed or what not. So. You call the lady say yeah.

Participant 6
I mean, so, so generally and yeah, but in another, in another setting, I mean, if you were to in studies where you analyse. And you know the presence of a certain contaminant and it's below the limit of detection. You usually use another number to just like to approximate or not put zero maybe, but I don't know how often that would happen in and would have been vitro talks test that thing. There are any example?

Participant 2
I think it happens quite frequently, right? This is these are all things that should be decided up front. How to handle this like a? A gene that falls beyond the city they sell this number of cycles for seeing, which could be ported. Generally speaking, right this year. So if it's 41, it's simply gets discarded, or shall we indicate A0, meaning no expression. These are all things that should be really, I think, decided up front to avoid possible bias. I didn't even stated in a publication, but it's never happened. Yeah. You.

Participant 5
I think that's the best as well. If you stayed in the publication, if it's of qPCR that you state you put the cut off at city 42 or 4, whatever and it states there so then you can put all the values that falls above that to that level that what you have stayed in the cut off to be. I think that's at least how we solve it because otherwise you don't have anything to count to, especially if you're if you're controls are very low and then you need to count to something otherwise you end up with. No results.

Participant 2
Yeah.

Participant 5
But also if you have essays where you have a standard curve there we see this happening quite a lot. So. In that case, we usually kind of put it to the detection limit, so the lower detection limit, but for those that go above the standard curve, we would not impute those, we would dilute it and do it again. So, but it's for the ones that are underneath, I think the detection limit that is a problem mostly I I assume most people will dilute and redo the analysis if you are above. At least US, yeah. How we do it?

Paul Whaley
So about data reduction, is this anything that you're familiar with or? Make use of. No, it's fine. Like it came up a lot in our tools, but that's not necessarily the case that it's a common thing that people think about. Or other techniques for. Normalising data rule, reducing noise. Things like that. So you kind of. Ohh manageable data set.

Participant 3
Yeah. When we are running the primary cells, it's also depends on. Who is on the ear isolating the cells and what time points during the day you or is isolate them, so that would affect your data, but you can get rid of this noise. And some tools.

Paul Whaley
So is is it would it would you do that as potentially an issue then like if you've got?

Participant 3
Yeah, because we saw it in an hour. Turned this steak data we were two person isolating cells and the data was kind of skewered. Dependent on the person isolating themselves.

Paul Whaley
Okay. So what we'll do is just look at someone else's bias issues that we found even more confusing. So. That was. Some tools talked about absence from analysis of predictors of missing data. Some talked about correcting for things like selection factors. Some were for controlling for baseline differences between groups. I think. So maybe we've touched on this a little bit like you've got not necessarily different like participant groups like you'd have in RCC. But if you've got say, two different investigators, you know in some period of time you've got one student. Ohh the passing out and what not and then add another period of time. We got another one and then you can see a difference because you got a very sensitive assay and you know which student was doing which of betting and then you do something to control for that potentially don't know if that's. Something that happens a lot. Um than the use of control data use of software. If you need these kind of. Inspire any thoughts?

Participant 2
I'm not really sure what we mean by predictors of missing data.

Paul Whaley
We are sorry though so.

Participant 2
OK. I mean, we're not talking about like um. And including a blank or having a. Secondary antibody only. Staying in Immunocytochemistry or even. Reference compounds. Not sure what we're talking about Love you. Longer.

Paul Whaley
So. And experience of particular choices of software introducing bias. Mean. What about control data? So I think I don't think there's a group we've talked much about concurrent or historical controls. Ohh posted negative controls. I think we touched on a bit yesterday, but I'm just wondering if. And the part for use of control data might distort. So the results of study and if you have any thoughts or experience around that? Hello I think you might have thought on that.

Participant 6
Yeah, well, well, yeah. And I'm. I'm not sure about in regards to. From. Control data. Of course. If you're using positive control. That can sometimes be tricky because you want to make sure that you're using that right positive control and not using the right controls could of course introduce. By you. But that's them. Yeah. We discussed that a bit yesterday too.

Paul Whaley
I just wondering maybe it's something cause this. I think this cause this is specifically to do with analysis of. Just wondering if there's a distortion in the analysis that can be introduced through the. The specific ways in which control data is analysed perhaps would be what these tools have been leaning towards.

Participant 6
Yeah, yeah.

Paul Whaley
But exactly, we found this difficult to interpret. Understand. So any ideas? Good ideas?

Participant 6
Yeah, yeah, I I mean, I don't have a lot of experience, but I I do understand from from talking to people that you know, depending on as we discussed yesterday, depending on which types of software you use or which type you, which way you sort of twist and turn your data to look, you can find different patterns. For example, if you're having like big data sets or make data, gene expression data you have. Which amounts of data points and you can, you know, look at it from different perspectives of of course you can twist and turn it to to search for certain patterns, but again comes back to maybe, and I completely agree that to have. Biostatisticians that do this for you so that you're not doing it yourself? So I don't. I'm not sure I can add anything more. Intelligent to it.

Participant 2
Ohh. I think the control data are very very critical. I'm just thinking about my abilities I toxicity also, if for some reason the viability I mean the road data that you would expect to see, don't know where the plate reader when you measure alamar blue. Is a number that do not just. I mean it's a number that does not make sense at all compared to your historical data. And then you start normalising whatever concentrations of chemical XYZ to that control. Clearly you're going to screw up your analysis. At least what that specific logical replicate and therefore. Most likely you should just discard the entire experiment from the data sets. This will be my easy problem to here. So again, appropriate characterization and ensuring that your negative control or solving control is performing as expected for a given endpoint and redout.

Paul Whaley
Okay. That sounds good. Yes. It's a tricky one. We didn't really understand what they were getting at. That sounds very good discussion. So this is a very difficult one. So. Perfect for 6:00 o'clock in the evening. Ohh, confounding kavari bias 24 items in the included tools. Part of this is because we were looking at some tools that had been developed for observational studies, so there are confounding issues that have been introduced by those, but we wanted to make sure that they didn't apply maybe to in vitro studies, but they were confounding things that look like they did. So we want to just go through what combining various is defined as. So it's a situation in which the effect were association between an exposure and an outcome is distorted by another variable. For confounding covariate bias to occur, the distorting variable must be associated with the exposure and the outcome. It must not be in the causal pathway between exposure and the outcome, and it must be unequally distributed between the groups being compared. Ohh yeah.

Participant 6
So just like ohh sorry.

Paul Whaley
That I didn't jump to the next one, that we ignore that.

Participant 6
No, I just had a gut reaction to, you know, unequally distributed with between the groups being compared. That, of course, there should not be any differences between your. Controls and your treated groups other than the concentration of the substance you're testing. But maybe. There could be. Factors that would. Influence so that you would have these differences between the. Pipe.

Participant 2
Like like for instance contaminating. Things in the in the chemical batch that you're testing or whatever. Something like this, yeah.

Participant 6
Yeah, I guess so if you're. If you have something. If you don't have them, yeah. If you have contaminants in the. In the chemical itself, in the test item.

Participant 2
Another thing that comes to my mind is the presence of precipitates. That should be of course cross cheque from the beginning with the nephelometer still be sure that you're chemicals is properly resolved in whatever solvent, but sometimes it may happens that precipitates form and therefore you do. I don't know. Maybe even you perform an analysis with the immunofluorescence and then you pick up signal. There is not really reliable just because you have precipitates that emit some kind of fluorescence and. So these are all things that may happen in the end, it's true. And you can see them in the control because it that's in the control with the solvent, which is pretty, for instance just the MSO and you have no precipitates in that case. That is something that happens to me once.

Participant 6
I was thinking also in terms of non materials that we touched upon yesterday also that Nana, if you're working with nanomaterials, maybe you have on you have all of these. You may have some other issues, but is particularly challenging with the with the material itself. Other aspects than it's. Proximity that affect them. The read up.

Paul Whaley
So the thing that we've just touched on, which is I think I think one of the major things that with the confounding covariates issue is baseline differences between the groups, right? So. One way to get a handle on this is obviously three random allocation of. Exposure to group right? So. We can talk a little bit about how much randomization there is in. In vitro study. Design. Like if it's um, you know you got maybe 10. Racks and an incubator and you take the top rack and you give that a lowest concentration. You take a second rack and give that second lowest concentration. Take the third ranking of the third last conversation. Then you put them back in that order. So then there's always, always the lowest on the top. And if there are different environmental conditions may be on that top shelf as to the bottom shelf, then you've got a baseline difference between the maintenance conditions of the highest exposure group versus the lowest exposure group. This happens with animals. I don't know if there could be something analogous. With. Uh. And they tried designs. It might be time varying issues where maybe the. Higher exposures makes not time having issues, just like the higher exposure. If the exposure itself may be affects cell density. So then there's a systematic difference in cell density between the more exposed and the less exposed cells, and that leads to changes in results. Just things like this potentially. Obviously can't really deal with that with randomization very easily. That's a confounds introduced by the experimental setup, but it's something that need to be taken into account. I'm saying a few nonsense so ohh.

Participant 2
Um. Yeah.

Paul Whaley
Yeah, sorry.

Participant 2
I I was just wondering. Yeah, I mean randomization of compounds distribution in a plate is highly desirable because events a lot of bias also in analysis, that analysis etcetera. The other thing that comes to my mind but but yeah, but it's not done on a regular basis based on my experience. Then again same considerations from yesterday discussion. Ohh, how this randomization is performed if it's manual. If it's done with a robot, so there are many different technical aspects to consider here in programming of the robotic platform, etcetera. Another thing that comes to my mind is the um problem of volatile compounds. You may have a gradient effect. You know if there is like a sort of cross contamination of chemicals flowing in other nearby neighbouring wells and stuff like this. And again, outer wells distribution if so should be put there or those should be discarded. I mean these are. Just things that come to my mind.

Paul Whaley
Participant 6 ohh sorry, might be on later.

Participant 3
I would tell you and if you have. If you have done, it's easier to randomise or if you are doing it yourself because you can introduce errors but pipetting wrong if you are. To random. Ohh yeah, it's just become confusing when you're competing, but I think it's better if you have a robot.

Paul Whaley
So you, participant 5, sorry. We've gotta give you that time. Lucky now. So you're not in very enthusiastically before Participant 2 even started. So that's usually a sign that I would of thought.

Participant 5
Yeah, I I had a thought. When you when you send your yeah, the points before, but I don't lost it but it was you talked about the time and I thought about that because we do some kind of air liquid interface exposure and there to get the higher dose you have to expose multiple time. So those cells are actually longer time in the exposure system than those that get the lower doses. This is how the system generally works. So there of course you can introduce some type of. Bias with how you treat your different exposure groups. Yeah, that was what I thought about. But the other thing you said regarding the. And density of the cells that that can also be affected by exposure. That is something we have seen in some of our experiments and really something that we had to go back and look at after to try to understand. What actually happened? Because it's affected everything downstreams. So if you are not aware and really looking at yourselves and and having yeah, because you have to really control your that your concentrations are equally happy otherwise you can just look at some downstreams marker and you think ohh this is fantastic but actually yeah something else happened with the source that you were not expecting. So it can of course be a bias. Now.

Paul Whaley
So just in relation to that and I kind of we're calling back a little bit here. So the annotators should be aware of this when we're doing this. But if you were. Have concerns that the exposure is having maybe more than the effect that you anticipate, so it maybe it's reducing cell density and therefore that's one of the things that's producing the results maybe confounding the results. Would you? Deal with that through redesigning the essay, potentially. Or would you deal with that through kind of correcting for these issues statistically later? Potentially. Think about you Participant 5. Since you've mentioned this but.

Participant 5
Yeah, I think it's it's a bit difficult because in this case we had we had a long term exposure of cells to non material that is quite known to be dangerous for cells and we had different type of carbon nanotubes, it was and then we saw that we get very different in the long term exposure we had the cells going for 28. Weeks. So in the long term exposure we saw that some of these cells that were with a particular type of nanoparticle actually had lower density after many, many weeks. So we had no chance to go back and redesign it kind of was what it was. So then we had to go back and try to figure out what happened and it was not, yeah, we didn't have a chance to go back and we had to kind of handle it in a different way statistically. So we go to different density. But that was part of unexpected effect. I guess that we had not counted for because it's not so many studies that have done that type of design. Yeah. So probably we could have handled it better, but in this long term this exposures you really can't do a trial before you do the exposure. So you just you you do a short term trial and then you do the long term exposure and. Things happened that we didn't expect. I it was not a good answer, but this is how we did it.

Paul Whaley
It's an excellent answer because we're just trying to get ideas down, you see. So there's a new. Say it's a new thoughts and ideas there. I think that we haven't seen in any of our previous discussions. This is really helpful. And Participant 4 are as I've I've left you out of the discussion a bit recently. So I just want to turn to you to see if you have any thoughts or comments on this.

Participant 4
Yeah, green with mostly everyone. I was thinking about the cell density and a it goes back to how we said before that if you know you're differentiation your model system yourselves and the differentiation, you're going to start at, you know a certain confluency. So like with the stem cells, we always start at a 70% confluency and it takes about four days. So we know that we wouldn't start testing our chemical anytime before that, could that could greatly lead to cell reduction and have the implication on the results. And then I was also thinking. You're working with air pollution samples and we protect. We specifically remove the particular matter because what can't go into that, what can't dissolve into that equally space like we get these little particulates out of left over the chemistry alone of those can like completely vary from well to well and with the cell types and so we removed them before because it can happen to different types of effects.

Paul Whaley
Maybe again. We're gonna call back again here because I think you just touched on something that we were talking about earlier, which was the choice of question bias. But now we're thinking, God, air pollution. There are particulates and air pollution. They don't enter, obviously kind of difficult to dissolve particulars and what not. If they're messing up the experiment because they're very kind of unpredictable units. Do you think that might have bearing then on how the?
You know, if you were making inferences about the toxicity of the. Type of air pollution exposing themselves to you think that could end up distorting the results? And if so, do you want to talk about that a bit more detail?

Participant 4
Oh, oh, yes, definitely. And we actually we just met with collaborators on this. Because of that, it's what we're gonna do is we're actually gonna test both, but independently. So we're gonna do the solid phase and one go, and then we'll do the particulates in another. And it's just because how they settle. But yes, it does. It can skew the, you know, if you're not on seeing the propriete questions and making generalised safe, I decided just to do the soluble feed lead out to the particular matter and then say that all air pollution inhibits bone development. But really. I didn't ask the question correctly. I didn't specify what I was actually testing. What, so yes. Sure.

Paul Whaley
Very good, very interesting. Okay. Um, 770 thoughts or comments at this point?

Participant 2
The last thing that comes to my mind is the maybe naive or stupid, but the pipette in the time. Your take to pipette your chemicals if you're doing it manually and you have to. Have many chemicals at different concentrations over, I don't know, 1096 well, plates or even OK, let's say 96 well plates to be more to be more realistic because otherwise you really need to work with the robot. So that's an issue that can be easily prevented when you're dealing with a robotic platform that just this spans everything altogether over 96 or 384, well played or whatever play format. But if you're doing it manually, clearly by the time you end up. Eating your last plates, unless you keep the plate in the incubator and you put them systematically, you know, with the help of someone else in the lab, then maybe you reduce this bias. But. You know, the cell may suffer a bit longer. They may, you know, the medium may start cooling down and stuff like this. . So these are all things to consider. I think confounding factors, let's say.

Paul Whaley
Okay anyone have so many thoughts? Mean is is there a risk cause like one of the things that makes confounding matter less is if the. If the factor isn't actually associated with the outcome, right? So if if people are, you know if you're passing out but for whatever reason that there's no, there's no pattern to the passing out that is associated with, like the eventual. Outcome, right? So you're not always doing the the, the the plates in the same order or anything like that. So that that's just going to be noise in the system rather than a distortion. Um, I just wondering in terms of the way that people go about. Kind of, you know, just organising themselves in the lab. If there are, if it's, if it's at all likely or probable that you could end up with these kind of systematic distortions. Because of the order in which people do things, and that's always the same, or if it's. That's just going to be noise in the system more likely.

Participant 2
Man 888 SA very subjective thing and people may organise themselves in different ways but. Depends really on the study design. How many chemicals on how many concentrations. So you should really to prevent any possible bias. Here you should define the approach, the experimental approach up front. Or some studies we were not even conceive considering uh manual pipetting things, it was just impossible to handle with. But for things that are in between medium proofs or low, medium, then then yes, you may decide to do everything manually and the robot should be used for more important studies okay let's do it manually. But then you have to take different actions to avoid any possible. You know, variability or stressing out the last plates longer than normal and stuff like this. So these are things to be decided up front where the study director and study personnel.

Paul Whaley
Alright, thank you. So I was having any thoughts or comments on this before we move on to the next domain? Okay. So we'll do the next One South we've got. Ohh, conflicted interests biases. So obviously controversial space cause we're trying to understand how interests of the. Researchers can distort the. Results or interpretation of the findings of the study. Um, it comes up a lot, but in the same way each time, so lots of tools talk about it, but they only talk about it in two ways so. Ohh complexion and transpires, we define as a bias and which decision makers influencing. Boston, which decision makers influencing research design, conduct analysis or reporting, have goals and motivations that conflict with scientific research objectives. So to put it another way, it's basically a split loyalties issue where for whatever reason, the researcher is, you know, supposed to be guided towards the truth when the. You know, conducting the study and interpreting the data, but they've got another interest that's pulling them in another direction, right? So it might be that the studies heading towards null results, that's not very exciting. Everybody wants to be interesting. So that motivation to be interesting leads to the selection of more favourable results or something like that because it's more interesting some. So I'm just wondering if you have any immediate thoughts or responses to this in terms of how people's interests can distort the results of an in vitro starting? There could be financial could be non financial. Ohh.

Participant 3
Probably a funding.

Paul Whaley
In what way specifically?

Participant 3
If it's a company that funded the research, of course they would, hopeful member. Some effects that maybe there is not there, but.

Paul Whaley
Ohh no, it's popped your webcam on so I think you have some say.

Participant 6
Yeah. Yeah, maybe. But it or the other way around, you know that. They don't want to see any results from their compound. They want to show that it states. But I think there's also a few alluded to, Paul. There's also the other. The other aspect that. You know a three searchers we also. You're biassed. Because you need to publish and you can only publish interesting results and then or positive results. Maybe so then you may be biassed towards finding things where. Or yeah.
Finding things where they there might not be anything. It goes both ways.

Paul Whaley
That's the other thing that you would specifically be looking for. In relation to people's interests that you might think. Either directly or indirectly indicate. Bias and results of a study.

Participant 6
You mean in terms of how the results have been or or study has been reported or? Any.

Paul Whaley
Anything you can think of, it's an open-ended questions.

Participant 6
I mean, I mean there should be a conflict of interest statement, the affiliations of the authors should be stated. . And nowadays there they often are. But if you look at older studies, this information might be missing. Yeah. That's obvious, maybe.

Paul Whaley
Yeah. Anyone else, Participant 4, maybe.

Participant 4
Yeah, I was thinking more of like, you know, reporting to your Funding Agency, your grant agency. You know, we have to turn in progress reports to NH every year. And and I'm just thinking like, I'm starting to prepare mine and it's like, you wanna show that this experiment is working there. You know, there are other things going on possibly. But I'm thinking of, like, selection of the selection of the data you choose to put in the report to show that, hey, what's your funding? Me is really for the right reasons, but. I mean. That's really it, but I have grad students. You know, they're the ones generating the data and doing it. So I would hope that. I'm not biassed by pulling somethings that were not there out of what they're handing.

Paul Whaley
So the things that the two that came up were sources of funding and conflicts of interest in general. So we, we do feel a bit light on specific information about how. Potential conflicts of info how conflicts of interest can affect or potentially distort the findings of study. One of the things we did want to cheque with you was this concept of mismanagement of interest. So the thing that's happening. In a conflict of interest situation is you've got a decision maker, someone who could influence directly influence how studies designed how it's actually conducted in physical practise, how the data is analysed and how it's reported, right? So we went on, was pulled into the directions. It can shape the any part of the study process. But um. Presumably it can only shape it if they're actually in a active decision making role, which suggests that it's not the interest per se, it's the management of the interest. So if there is a conflict that measures should be taken to remove somebody from being in a decision making role of that conflict can actually change things. But I'm just wondering if you've got any thoughts on that specifically.

Participant 6
What I have colleagues that not so much anymore, but. To. That's years ago had funding from industry to do certain studies. Uh, but where they claim that. You know, OK, we received this funding to do this in to do the these studies, but the people funding us are not involved in designing this study. They're not involved in collecting any results or analysing any results. They're not involved in writing up the the paper or they're nothing involved in authoring or the publication process in anyway. But you're still you're still wonder if if some at some point then. In the chain of events that some influence might have been made, you don't really know. Of course, that means. But you don't like to think that way, but. Could happen course.

Paul Whaley
So if we just interrogate that a little bit more, just so the, what other influences besides active decision making then do you think could be apply,?

Participant 6
I don't know, but like implicit threats of removing funding as researchers, so your your so extreme, especially in the academic setting, you're so. Vulnerable to, you know, funding. You're so reliant on funding being given to you. So I guess that, you know, implicit threats of funding being withdrawn unless they get to look at the data before you publish or whatever then. But.

Participant 5
This is.

Paul Whaley
You don't have to defend.

Participant 6
But I mean that that's not supposed to happen, but I don't know if it's never happened to me, but I don't know if it could happen. I I have no idea. I'm just throwing things out there now, since you're putting me on the spot. Yeah.

Paul Whaley
You don't like bad ideas. You don't have to defend it or just trying to like pull out of people stuff that we haven't seen in the included tools, right? So we this might not make it through to the final cut. It may do. Like that, yeah.

Participant 6
But you said, yeah, I don't know how you would. I don't know how you would catch that in in, in a in. In a I mean cause that's not gonna be, you know, you're not gonna get that information. You're gonna. You're not gonna see that. So get the things that you can see are the, you know who funded the study, who what are the authors affiliations. And the did they declare any conflict of interest? I mean that's that's all you can really touch when you look at the publication. The rest is just guesswork, and that's dangerous. You cannot start guessing if.

Paul Whaley
Indeed.

Participant 6
They're potentially, yeah.

Participant 5
Yeah, I have a comment to follow up on this from Participant 6, because we are working a lot with industry and a lot of our projects are, you know, in collaboration with industry, we don't, we don't get funding from them, but we are going there and taking samples and working with with the exposure. And and of course industry every time. And have the possibility to to say that they refuse us to to publish some of the data that we received from them. So usually we write an agreement beforehand with them that they cannot, you know, the data we receive are our data and they cannot, you know, say afterwards that we can't publish them. But I guess this is not something you would put in into a publication. It would not say anywhere that we have that agreement, but that's an agreement that we usually every time, right, otherwise we would not go into that industry.

Paul Whaley
Right.

Participant 5
And but also one thing that we have on our industrial related projects is that we every time have a reference group. So in that it would be the partners from the industry and from the workers and employers sector. And so that we have kind of covered the baseline. There are representatives from all the partners in the in the work life that are. And in in the meeting Sunday in in the decision making. So I think but that's also not something we report on in the articles, but this is common practise at least. Yeah, that's awesome. I think many of the people that are working with industry have this type of practise the avoid this type of. Affection of yeah. Yeah. Bias in in the decision making. But I don't think there is any practise for reporting that in, in an article. Maybe it should, maybe it should be, I don't know. Yeah.

Paul Whaley
Worry about that further down. Yeah, there's gonna be a lot of implications to reporting. Probably by the time we've put together this toolkit. So I realised we're at the bottom of the hour, so I don't want to keep you any longer than we agreed. Um, it's been incredibly helpful. We've actually basically got through everything. So I think I just want to thank you all for your just going to turn off my screen sharing hold on a second, there we go. Service. Thank you for your energy and enthusiasm and such like lively and engaged discussion has been I've done what 9 hours of focus groups now and my brain is completely fried. Just having so much stuff shoved into it by such interesting and knowledgeable researchers has been wonderful. So thank you very much and for your patience as well because I'm a methodologist, so I help people design studies like this. I don't actually do any lab work myself. Sure, it's entirely obvious that's the case. Yeah. So just yeah, thank you ever so much and we'll be in touch about the. Uh findings or the the how the studies progressing as it develops, we will be running at Delphi process next once we've got it, will all the data that we've been gathered has been kind of coded up. So probably going to have to code up another 400. Uh ohh its criteria which will give us about 800 in total, which we then need to distil down into. Manageable number of questions and. Prompts for people to answer, so we'll have fun doing that. Good. Do you want to say anything at this point?
