# Supplementary material for: A comprehensive item bank of internal validity issues of relevance to in vitro toxicology studies
Source: Evid Based Toxicol. Author manuscript; Available in PMC 2025 Oct 31. (PMC12180937; doi:10.1080/2833373X.2024.2418045)
Supplement: Supplements [file NIHMS2054894-supplement-Supplements.zip › Supplemental Material 6_Item Bank_Code for Figure 2_R1.docx]

Inventorylist_figure4

2024-01-25

Table of Contents

# Loading packages

library(ggplot2)
library(ggpubr)
library(fitdistrplus)
library(openxlsx)
library(tidyverse)

# Get work directory and organise results

HOME <- "C:/Users/TRHU/Documents/R/PARC_VKM_Focus"
setwd(HOME)

Create a folder with current date in the Result folder

newday <- file.path('C:/Users/TRHU/Documents/R/PARC_VKM_Focus/Results', Sys.Date())
dir.create(newday)

# Read in data

Inventory_domain <- read_delim("C:/Users/TRHU/Documents/R/PARC_VKM_Focus/Data/Trine_Figure4_new.csv")

# Make figures

Make a long table from the summarydata

Inventory_domain_long <- Inventory_domain %>% pivot_longer(cols = !Domain, names_to = "Source", values_to = "Count")


# lock in factor level order
Inventory_domain_long$Source <- factor(Inventory_domain_long$Source, levels = c("Cooper","IRIS", "OHAT", "ROB2", "ROBINS-E", "SciRAP", "SR", "Focus Groups", "All sources" ))

BiasDomain_Source <- Inventory_domain_long %>%
 ggplot(aes(x = fct_rev(Domain), y = Count, fill = fct_rev(Source))) +
 geom_col(position="dodge") +
 facet_grid( ~ Source, labeller = label_wrap_gen(width=20)) +
 coord_flip() +
 geom_text(aes(label = round(Count, 0)), size = 4, hjust = -.2) +
 theme_bw() +
 theme(legend.position = "none") +
 theme(axis.line = element_line(color='black'),
 plot.background = element_blank(),
 panel.grid.major = element_blank(),
 panel.grid.minor = element_blank())+
 #panel.border = element_blank())+
 theme(strip.text.x = element_text(face = 'bold', size = 25),
 strip.background = element_rect(fill="white", colour="black",size=1)) +
 theme(axis.title = element_text(size = 22),
 axis.text.x = element_text(face = 'bold', size = 10),
 axis.text.y = element_text(face = 'bold', size = 11),
 legend.text = element_text(size = 11),
 legend.title = element_text(size = 11),
 legend.key.size = unit(1, 'cm'),
 plot.title = element_text(size = 10),
 strip.text.x = element_text(size = 8), #change the facets font
 plot.caption = element_text(face = 'bold', size = 15),
 plot.subtitle = element_text(size = 15))+
 labs(y = "Source (# items)", x = "Bias Domain")

BiasDomain_Source


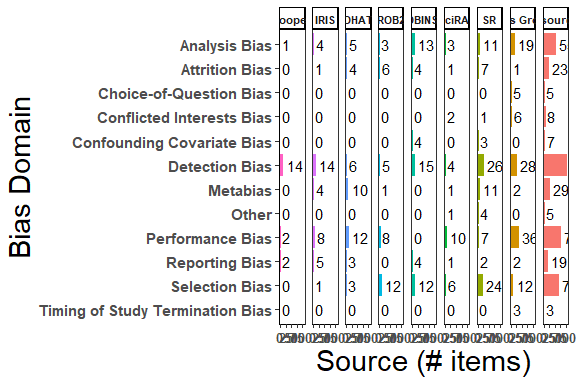


ggsave(filename=file.path(newday, "BiasDomain_Source.jpeg"),
 device = NULL,
 width=NA,
 height=NA,
 units="mm")
